# The Potential Impact of a Single-Dose HPV Vaccination Schedule on Cervical Cancer Outcomes in Kenya: A Mathematical Modelling and Health Economic Analysis

**DOI:** 10.3390/vaccines12111248

**Published:** 2024-11-01

**Authors:** Grace Umutesi, Christine L. Hathaway, Jesse Heitner, Rachel Jackson, Christine W. Miano, Wesley Mugambi, Lydiah Khalayi, Valerian Mwenda, Lynda Oluoch, Mary Nyangasi, Rose Jalang’o, Nelly R. Mugo, Ruanne V. Barnabas

**Affiliations:** 1 Department of Global Health, University of Washington, Seattle, WA 98105, USA; 2 Division of Infectious Diseases, Massachusetts General Hospital, Boston, MA 02114, USA; 3 University of Washington School of Medicine, Seattle, WA 98195, USA; 4 The Norton College of Medicine, Upstate Medical University, Syracuse, NY 13210, USA; 5 National Vaccines and Immunization Program, Ministry of Health, Nairobi P.O. Box 30016-00100, Kenya; 6 National Cancer Control Program, Ministry of Health, Nairobi P.O. Box 30016-00100, Kenya; 7 Kenya Medical Research Institute, Nairobi P.O. Box 19865-00202, Kenya; 8 Department of Medicine, Harvard Medical School, Boston, MA 02115, USA

**Keywords:** HPV vaccination, single-dose, cost-effectiveness analysis, mathematical modelling, low-and-middle income country, Kenya

## Abstract

**Background**: Human Papillomavirus (HPV) is the primary cause of cervical cancer. Single-dose HPV vaccination can effectively prevent high-risk HPV infection that causes cervical cancer and accelerate progress toward achieving cervical cancer elimination goals. We modelled the potential impact of adopting single-dose HPV vaccination strategies on health and economic outcomes in Kenya, where a two-dose schedule is the current standard. **Methods**: Using a validated compartmental transmission model of HPV and HIV in Kenya, we evaluated the costs from the payer’s perspective to vaccinate girls by age 10 with either one or two doses and increasing coverage levels (0%, 70%, 77%, 90%). Additionally, we modelled single-dose strategies supplemented with either catch-up vaccination of adolescent girls and young women or vaccination for all by age 10, funded with the first five-years of cost savings of switching from a two- to one-dose schedule. Costs and outcomes were discounted at 3% annually, and incremental cost-effectiveness ratios (ICERs) were calculated per disability-adjusted-life-year (DALY) averted. **Results**: All one-dose and the two-dose 90% coverage strategies were on the efficiency frontier, dominating the remaining two-dose strategies. The two-dose 90% coverage strategy had a substantially higher ICER (US$6508.80/DALY averted) than the one-dose 90% coverage (US$197.44/DALY averted). Transitioning from a two- to one-dose schedule could result in US$21.4 Million saved over the first five years, which could potentially fund 2.75 million supplemental HPV vaccinations. With this re-investment, all two-dose HPV vaccination scenarios would be dominated. The greatest DALYs were averted with the single-dose HPV vaccination schedule at 90% coverage supplemented with catch-up for 11–24-year-old girls, which had an ICER of US$78.73/DALYs averted. **Conclusions**: Considering the logistical and cost burdens of a two-dose schedule, a one-dose schedule for girls by age 10 would generate savings that could be leveraged for catch-up vaccination for older girls and accelerate cervical cancer elimination in Kenya.

## 1. Introduction

Human papillomavirus (HPV) is the primary cause of cervical cancer, the fourth leading cause of female cancer worldwide [1]. In Kenya, there are over 5236 new cases and 3211 deaths due to cervical cancer annually [2]. High-risk HPV infections are responsible for almost all cervical cancer cases and HPV vaccination is an efficacious prevention strategy [3,4,5]. To achieve Cervical Cancer Elimination by 2030, the World Health Organization (WHO) has set these goals: vaccinate 90% of girls by age 15, screen 70% of women by age 35 and again by age 45, and treat 90% of women diagnosed with pre- or invasive cancer [6]. 

The Kenyan Ministry of Health (MoH) launched the national HPV vaccination program in 2019 through the National Vaccines and Immunization Program (NVIP). The program aimed to deliver two doses of the quadrivalent HPV vaccine in health facilities, six months apart, initially to 10-year-old girls, although provision was expanded in 2021 to a multi-age cohort of girls 10–14 years [7,8]. However, HPV vaccination uptake has remained low; in 2021, only 31% of 10-year-old girls received the recommended two doses of the vaccine, while 77% received one dose [9]. Additionally, Kenya is expected to graduate from Gavi support for the HPV vaccine over the next five years, which could result in costs exceeding US$10 Million per year [9]. 

High-quality evidence supports the efficacy of a single-dose HPV vaccination. Randomized trials [10,11], a meta-analysis [12], observational studies [13,14,15,16], and modelling analyses [17,18,19,20,21] demonstrated the efficacy and population-level effectiveness of one-dose HPV vaccination in different contexts, including low- and middle-income countries. A recent study from Scotland also showed that no cervical cancer cases were detected in vaccinated women following HPV immunization at age 12–13, irrespective of the number of doses [22]. This body of evidence supports the WHO recommendation of a one or two-dose schedule for girls and boys aged 9–20 years to increase coverage and catalyze progress toward cervical cancer elimination goals [23]. The WHO Africa regional office has also recommended that countries transition to single-dose HPV vaccination to reach more adolescent girls and young women [24]. In Kenya, the KEN SHE Study, a randomized trial conducted in 3 counties, has shown high efficacy of a single-dose of the HPV vaccine [10]. However, there is uncertainty over the duration of equivalent protection as the earliest single-dose study has not yet reached 20 years [14]. The Costa Rica HPV Vaccine Trial (CVT) has shown durable protection of a single-dose HPV vaccine for at least 16 years [14,25]. 

As Kenya explores strategies to accelerate cervical cancer elimination, a modelling and health economic analysis would inform the potential implications of switching to a single-dose HPV vaccination schedule, including the long-term outcomes and cost. We leveraged modelling analyses to perform three evaluations: (1) the cost-effectiveness of one-dose delivered in health facilities compared to a two-dose HPV vaccination schedule under various coverage scenarios, (2) the impact of potential waning of one-dose HPV vaccine efficacy on cervical cancer and health economic outcomes, and (3) the added impact of using the five-year cost savings of switching from a two- to one-dose HPV vaccination schedule for either catch-up vaccination for multi-age cohorts (MAC) of girls and young women or vaccination for all by age 10 strategies (i.e., vaccination of both girls and boys).

## 2. Methods

### 2.1. Model Description

We used a validated compartmental, dynamic model of heterosexual HPV and HIV transmission calibrated to the Kenyan setting, which has been described previously [26,27]. The model simulates HIV and HPV transmission, development of cervical intraepithelial neoplasia (CIN), and progression to cervical cancer. Interventions simulated in the model include HIV treatment, cervical cancer screening, and HPV vaccination. Simulating both HIV and HPV is important because Kenyan women have an HIV prevalence of approximately 6%, and women living with HIV have a six-fold higher risk of cervical cancer compared to women without HIV [28,29,30]. 

The model dynamics are based on a system of partial ordinary differential equations solved in MATLAB using a 4th order Runge-Kutta numerical method. The model begins in 1925, HIV infection is introduced in 1980, and it reflects historical HIV prevention strategies (such as voluntary male medical circumcision and condom use), antiretroviral therapy coverage, HPV vaccination, and cervical cancer screening coverage levels. Because the model is dynamic, it can capture population-level effects such as herd immunity. The model output was calibrated and validated independently to Kenyan data. Detailed descriptions of model processes, calibration, and parameters are found in the Supplementary Materials [26]. 

### 2.2. Modelled Scenarios

Beginning in 2019, we modelled two-dose HPV vaccination of girls by age 10 in Kenya with the quadrivalent HPV vaccine, and with coverage increasing linearly to present-day levels of 31% by 2023 [7]. Starting in 2023, we simulated a baseline scenario of no further HPV vaccination and scenarios varying vaccine dose, durability, and coverage. Using methods described in previous publications [31], we simulated uncertainty in vaccine efficacy of one- and two-doses by randomly selecting values from a beta probability distribution representing efficacy results from three-dose and one-dose trials. Two-dose vaccine scenarios reflected the quadrivalent vaccine efficacy results from the FUTURE I and II trial of 100% (95% CI 88.4, 100) [32], and one-dose vaccine scenarios reflected the bivalent vaccine efficacy results from the KEN SHE trial of 97.5% (95% CI 90.0, 99.4), which was similar to the results of the nonavalent vaccine against HPV 16 and 18 of 98.7% (95% CI 90.5, 99.8) [10]. Although the KEN SHE trial did not study the efficacy of the quadrivalent vaccine, we assumed efficacy would be similar to the bivalent and nonavalent vaccines since high-risk types HPV16 and HPV18 are targeted by both vaccines, and the nonavalent vaccine is based on the quadrivalent vaccine technology. In the paired draws, we conservatively assumed that one-dose vaccine efficacy was always equal to or lower than two-dose efficacy for each parameter set. We also ran a scenario of single-dose vaccination of girls by age 10 at 90% coverage without this restriction as a sensitivity analysis (Supplementary Materials Section SIV). 

For the first evaluation objective of the study, we assumed lifelong efficacy of both one- and two-dose vaccination and simulated scenarios of increasing vaccination coverage, including a hypothetical baseline of 0% future coverage. We modelled two-dose scenarios with coverage remaining at current levels of 31%, and scenarios of increasing coverage of 50%, 70%, 77%, and 90% [7]. Similarly, we ran one-dose scenarios with coverage remaining at current levels of 77%, as well as 70% and 90%. These scenarios aimed to inform the health and financial trade-offs of achieving different coverage levels with different vaccination dosing schedules. 

The second objective was to evaluate the potential impact of one-dose waning efficacy possibilities on cervical cancer outcomes. We modelled one-dose scenarios of combinations of an efficacy period between 20–30 years and a waning period of 10 or 20 years. The efficacy period (EP) is the duration of full vaccine efficacy, and the waning period (WP) is the duration of time in which vaccine efficacy declines linearly to 0%. We selected 20 years as the minimum EP because recent evidence suggests that one dose of the HPV vaccine maintains full efficacy up to at least 16 years [25]. We selected 30 years as an alternative EP since it would protect women against HPV acquisition and provide coverage through ages with high observed incidence of cervical cancer [33]. For these scenarios, we maintained vaccination coverage at 90%. 

For the third objective, we calculated the expected five-year cost savings if Kenya switched from a two- to a one-dose HPV vaccination schedule, and simulated investing the savings into additional one-dose HPV vaccinations, including either (i) vaccination for all by age 10 inclusive of boys and girls, (ii) catch-up vaccination of 11 to 19 year-old girls to align with the current WHO recommendation for one-dose vaccination [34], or (iii) catch-up vaccination of 11 to 24 year-old girls and young women to cover a wider age of women. We ran these alternative supplemental vaccination strategies alongside a primary strategy of one-dose HPV vaccination of girls by age 10 at 90% coverage. We modelled each vaccination strategy under three durability scenarios: lifelong vaccine efficacy, pessimistic waning efficacy (20 years EP, 10 years WP), and alternative waning efficacy (30 years EP, 20 years WP). These scenarios aimed to identify whether cost-savings from reduced dosing can fund supplemental strategies capable of overcoming the potential effects of waning one-dose vaccine efficacy. 

For all scenarios, we modelled a constant one-time cervical cancer screening coverage at their current national averages of 14% for women without HIV [2] and 56% for women with HIV [35], both at ages 35–39 years. Screening assumed visual inspection with acetic acid, triage with colposcopic biopsy, and those diagnosed with CIN2 or greater were modelled as treated with ablative therapy. As a sensitivity analysis, we ran a one-dose scenario at 90% coverage with lifelong efficacy, assuming a switch to cervical screening with HPV-DNA at the same coverage and then an additional scenario with HPV-DNA and treatment by ablative therapy (Supplementary Materials Section SIV). To account for uncertainty in sexual behavior, HIV and HPV natural history, and HPV vaccine efficacy, we ran 25 parameter sets for each scenario. 

### 2.3. Epidemiologic Outcomes

To evaluate the impact of vaccination strategies on cervical cancer outcomes, we estimated annual age-adjusted cervical cancer incidence rates from 2023 to 2123, standardized using the WHO 2000–2025 Standard Population distribution [36]. We used the results from the no further vaccination scenario as a reference. We evaluated: (1) cumulative cancer cases averted, (2) percent reduction in incidence, and (3) mortality for each scenario compared to the reference. Finally, we calculated the total number of HPV vaccines administered, number of vaccines required to avert one case of cervical cancer, and the year at which the elimination threshold would be reached (incidence rate of 4 per 100,000 women) [6]. Results are reported as a median of 25 parameter sets, and uncertainty as a 90% confidence interval (CI). 

### 2.4. Costs and Economic Outcomes

Costing and economic analyses were conducted from the Kenyan Ministry of Health perspective, including only direct medical costs incurred and averted. Disability-adjusted life-years (DALYs) were calculated from model outputs, disability weights for cervical cancer health states derived from the 2019 Global Burden of Disease [37], and life expectancy estimates from WHO life tables [38]. Cost estimates were extracted from published studies. HPV vaccination costs are described in Table 1, and account for the quadrivalent HPV vaccine (US$4.50 per dose), commodities including syringes and safety boxes, international handling (3%), delivery fees (10%), and wastage (5%) [39,40]. Additionally, we used an estimated cost to the health system of US$4.97 per dose for vaccine delivery within health facilities [9]. To account for Kenya’s expected graduation from Gavi support for the HPV vaccine, we applied a cost per dose of US$0.50 in 2020, and, starting in 2021, increased the cost gradually from 20% of the vaccine price to 100% by 2027 (20% in 2021, 25% in 2022, 30% in 2023, 46% in 2024, 62% in 2025 and 80% in 2026) [9]. Since our analysis accounted for a starting point after the launch of the HPV vaccination program in Kenya, we did not include a Gavi Vaccine introduction grant for routine immunization that would have been available to the country in the first year (2020) of the HPV vaccination program. We also accounted for aggregated costs of cervical cancer screening, triage, and cervical pre-cancer and cancer treatment. Costs were converted to 2023 US dollars (USD). Both costs and outcomes were projected over a 100-year time horizon from 2023 to 2123 and discounted at an annual rate of 3% following standard practice [41].

Five-year cost-savings of switching from a two- to one-dose schedule were estimated by comparing all possible two-dose coverage levels with a switch to all possible one-dose coverage levels. These pairwise comparisons were made rather than only comparing two with one-dose schedules at equivalent coverage levels since changing the dosing could allow an expansion of HPV vaccination coverage. To be conservative, we used mean cost estimates for all one- and two-dose scenarios at each coverage level. The lowest possible five-year cost-savings of switching to a single-dose schedule and the median discounted cost per vaccination (including costs of the vaccine, supplies, and to the health system for facility-based vaccination) were used to estimate the number of additional HPV vaccine doses that could be administered using a supplemental strategy. Supplemental catch-up strategies targeting girls over age 10 were modelled to happen once over the first five years (2023–2028) while vaccination for all by age 10 strategies were modelled to persist over the full-time horizon (2023–2123). 

The comparative performance of each scenario was evaluated using the incremental cost-effectiveness ratio (ICER), calculated as the difference in cost divided by the difference in health (in DALYs) of one strategy compared with the next less costly strategy. Strategies that were more costly and less effective than an alternative (strongly dominated) or had higher ICERs compared to a more effective alternative (weakly or extended dominated) were considered inefficient and removed from the calculations in the analysis following standard practice [41]. For all non-dominated scenarios, we reported the median ICER between adjacent non-dominated scenarios from simulations using the 25 best-fitting parameter sets, along with the 90% confidence interval. The cost-effectiveness analyses were conducted using R (version 4.2.1) and Stata (version 18). We reported our results according to HPV-FRAME, a consensus statement and quality framework for modelled evaluations of HPV prevention, and Consolidated Health Economic Evaluation Reporting Standards (CHEERS) 2022, the guidance for reporting health economic evaluations [42,43] (Supplementary Materials Section SVI).

### 2.5. Cost Sensitivity Analysis

One-way sensitivity analyses were conducted for each scenario to assess the impact of changes in each economic parameter on ICERs using range estimates extracted from published literature (Table 1). The following cost categories were investigated: Gavi contribution, procurement, health system delivery, supplies, screening, and treatment. Using in-country expert input, we increased Gavi contributions by 10% every year starting from a 30% contribution in 2023 (lower bound) or a 40% contribution in 2023 (upper bound) until the full cost of the vaccine to be covered by the country was reached. Results from one-way sensitivity analyses were presented using tornado diagrams to showcase changes in the ICERs compared to the no vaccination scenario. 

**Table 1:** Key model inputs and costs.

**Key Model Inputs**	**Inputs**		
HPV routine vaccination			
Doses	1–2		
Two-dose vaccine efficacy	Beta distribution (α = 10.76 and β = 0.15) to reflect 100% (95% CI 88.4, 100) [32]		
One-dose vaccine efficacy	Beta distribution (α = 49.7 and β = 1.6) to reflect 97.5% (95% CI 90.0, 99.4) [10]		
Historical two-dose coverage of girls by age 10	Linear scale-up of coverage from 0–16% between 2019–2020, and then 16–31% between 2020–2023 [7]		
Two-dose coverage of girls by age 10	31% (current two-dose coverage), 50%, 70%, 77%, 90% [7]		
One-dose coverage of girls by age 10	70%, 77% (current one-dose coverage), 90% [7]		
One-dose vaccine durability	Lifetime efficacy; combinations of EP 20, 25, 30 years and WP 10, 20 years		
Two-dose vaccine durability	Lifetime efficacy ^a^ [18,31]		
Additional vaccination strategies	Vaccination for all boys and girls by age 10 (90% coverage), age 11–19 catch-up of girls (46% coverage), age 11–24 catch-up of girls (33% coverage)		
Cervical cancer screening	Screening with VIA, triage with colposcopy, and treatment with cryotherapy [35,44,45]		
**Costs ^b^**	**Estimate (Range) [Source]**	**Costs, continued ^b^**	**Estimate (Range) [Source]**
**HPV Vaccine**		**Vaccine delivery**	
Cost of quadrivalent vaccine per dose (USD)	4.5 (4.5–4.5) [9,39]	Incremental health system costs (USD)	4.97 (3.98–5.98) [46]
Wastage (% of price)	5 (4–6) [9,39]	**Screening & Treatment**	
International handling fee (% of price)	3 (2–4) [9,47]	VIA (USD)	5 (5–5) [46]
International delivery fee (% of price)	10 (8–12) [9,48]	Colposcopy (USD)	33.92 (27.00–40.79) [48]
**Syringes**		Ablative therapy	50.56 (50.56–50.56) [46]
Price per dose ^c^ (USD)	0.07 (0.06–0.08) [9,40]	**Cervical Cancer**	
Wastage (% of price)	5 (4–6) [9,39]	Diagnostics (Biopsy and histopathology)	36.19 (36.19–36.19) [46]
International handling fee (% of price)	3 (2–4) [9]	Hysterectomy–radical	493.6 (493.6–493.6) [49]
International delivery fee (% of price)	10 (8–12) [9]	Local cervical cancer	1279.10 (1021.23–1532.69) [9]
**Safety boxes**		Local cervical cancer (without hysterectomy)	391.31 (311.84, 469.44) [46]
Price per box (USD)	1.3 (1.3–1.3) [9,40]	Regional cervical cancer	6447.33 (5174.22–7746.97) [9]
Total number of syringes per safety box	100 [9]	Regional cervical cancer (without hysterectomy)	5086.06 (4087.13–6113.59) [46]
Price per syringe/dose (USD)	0.0103 (0.0103–0.0103) [9]	Distant cervical cancer	5086.05 (4084.71–6096.59) [9]
Wastage (% of price)	5 (4–6) [9]	**Disability Weights**	
International handling fee (% of price)	3 (2–4) [9,47]	Local cervical cancer	0.288 (0.193–0.399) [37,48]
International delivery fee (% of price)	10 (8–12) [9,48]	Regional cervical cancer	0.451 (0.307–0.600) [37,48]
		Distant cervical cancer	0.540 (0.377–0.687) [37,48]

^a^ Two-dose lifetime efficacy is a conservative assumption that was made for this study, although there is not enough longitudinal data yet to confirm the validity of this assumption. ^b^ Costs are shown in U.S. Dollars (USD). ^c^ Full cost after Gavi graduation.

## 3. Results

### 3.1. Assuming Single-Dose Lifelong Efficacy

Maintaining a two-dose schedule and current coverage of 31% for girls by age 10 would result in a cervical cancer incidence of 14.6 per 100,000 women (5.6–23.8) compared to the no vaccination scenario of 26.0 per 100,000 women (12.3–39.2), a percent reduction in incidence of 33.4% (20.0–51.9) (Table 2). Increasing coverage levels will continue to reduce cervical cancer incidence, and similar trends are also observed for cervical cancer mortality rates. For the two-dose 90% scenario, cervical cancer incidence is expected to reach the elimination threshold of 4 per 100,000 women by 2094, and at our 100-year time horizon from 2023, the incidence is expected to fall to 2.9 per 100,000 women (1.6–4.9). 

**Figure 1:**
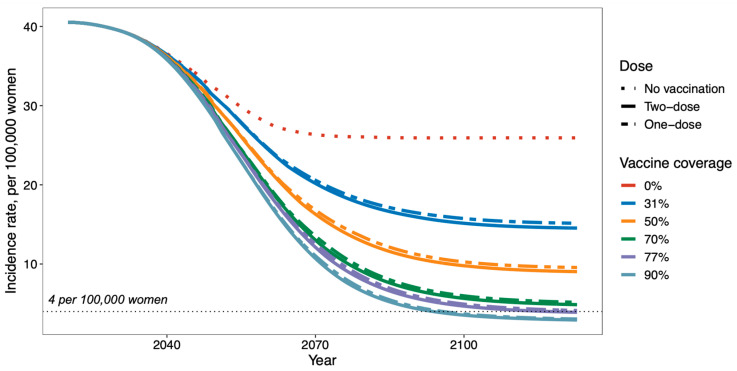
Projected cervical cancer incidence over 100 years under scenarios of no vaccination, two-dose vaccination with increasing coverage, and one-dose vaccination with increasing coverage. The plotted lines represent the median incidence from 25 parameter sets. The horizonal dotted line shows the cervical cancer elimination threshold of 4 per 100,000 women.

If Kenya switches to a one-dose HPV vaccination strategy for girls by age 10, maintaining a one-dose schedule at the current one-dose coverage of 77% is expected to reduce cervical cancer incidence to 4.1 per 100,000 women (1.7–8.0) by 2123. For a two-dose strategy at the same coverage level (77%), the cervical cancer incidence is expected to be slightly lower at 3.9 per 100,000 women (1.7–7.3). For the one-dose 90% scenario, cervical cancer incidence is expected to reach the elimination threshold of 4 per 100,000 women by 2096, and at our 100-year time horizon from 2023, the cervical cancer incidence is expected to fall to 3.1 per 100,000 women (1.6–5.3). Figure 1 illustrates age-adjusted cervical cancer incidence for all one- and two-dose scenarios with varying coverage levels. 

Despite one-dose scenarios having incidence levels slightly higher than two-dose scenarios at the same coverage level, all one-dose scenarios required roughly half the number of vaccines to avert one case of cervical cancer compared to two-dose scenarios. For example, the two-dose scenario at 90% coverage required 21.4 (14.6–45.0) thousand vaccines to avert one cervical cancer case, while the one-dose scenario at the same coverage level required 11.0 (7.4–22.7) thousand vaccines to avert one cervical cancer case (Table 2). 

**Figure 2:**
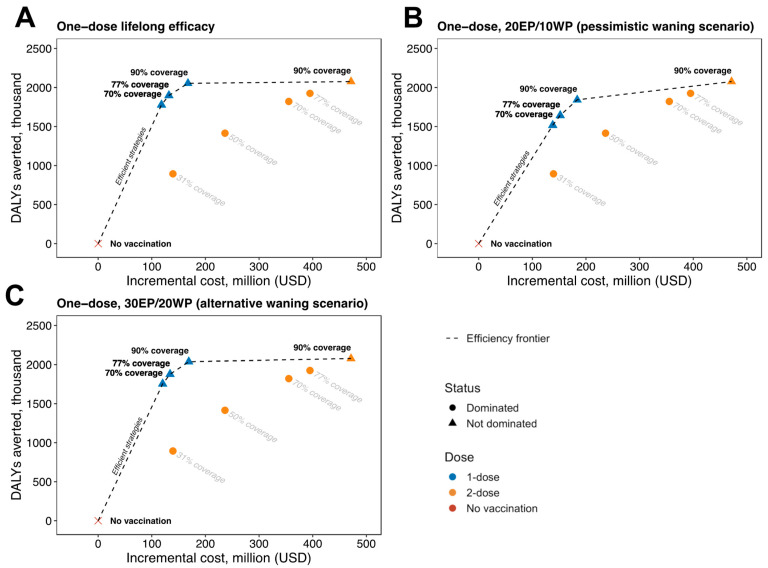
Cost-effectiveness analysis of 10-year-old girl vaccination at different coverage levels and HPV vaccine doses, with different assumptions for one-dose HPV vaccine durability of (**A**) lifelong efficacy, (**B**) pessimistic waning of 20 years of efficacy and 10 years of waning, and (**C**) alternative waning of 30 years of efficacy and 20 years of waning. The graphs display the discounted incremental costs (*x*-axis; in 2023 USD) and DALYs averted (*y*-axis) over a 100-year time horizon compared to no vaccination. The cost-effectiveness of changing from one strategy to a more costly alternative strategy is calculated by taking the difference in cost and dividing it by the difference in DALYs of the two strategies. The dotted line represents the strategies that are efficient because they are more effective and either cost less or have a more attractive cost-effectiveness ratio than less effective options. The ICER is the reciprocal of the slope of the line connecting the two strategies under comparison. All points represent the median of 25 parameter sets. Abbreviations: EP, efficacy period; WP, waning period.

All one-dose scenarios and the two-dose 90% coverage scenario were considered efficient (non-dominated strategy on the efficiency frontier), as shown in Figure 2A. Compared to the next non-dominated strategy, the two-dose 90% coverage scenario had an ICER of US$6508.80 per DALY averted, higher than the one-dose 90% coverage scenario that had an ICER of US$197.44 per DALY averted (Table 3).

### 3.2. Assuming Waning Efficacy for Single-Dose Scenarios

Under various possible scenarios of single-dose HPV vaccine waning efficacy at 90% coverage, cervical cancer incidence after 100 years ranged from 3.1 (1.6–5.6) per 100,000 women for the 30 EP/20 WP scenario to 4.6 (1.7–10.2) per 100,000 women for the 20 EP/10 WP scenario. All waning scenarios of single-dose vaccination are expected to avert fewer cases of cervical cancer compared to a two-dose scenario at 90% coverage. However, the pessimistic waning efficacy scenario for one-dose (20 EP/10 WP) would still reduce the number of vaccines needed to avert one cervical cancer case to 12.6 (9.0–23.9) thousand vaccines, substantially lower than the required number of vaccines (21.4 [14.6–45.0] thousand vaccines) to achieve a similar outcome for the two-dose scenario (Table 2). 

The efficiency frontiers for the pessimistic (Figure 2B) and alternative (Figure 2C) waning scenarios were similar to the lifelong efficacy results; all the one-dose and the two-dose 90% coverage scenarios were on the efficiency frontier. Under the pessimistic and alternative waning assumptions, the ICERs of the two-dose 90% coverage scenario compared to the one-dose 90% coverage scenario were US$1087.49 per DALY averted (pessimistic waning) and US$4736.59 per DALY averted (alternative waning). Comparatively, the one-dose 90% coverage scenario for the pessimistic and alternative waning scenarios compared to the next lower cost and non-dominated strategy (one-dose 77% coverage scenario) had ICERs of US$168.55 per DALY averted (pessimistic waning) and US$191.95 per DALY averted (alternative waning).

### 3.3. Assessing the Impact of Supplemental Vaccination Strategies

**Figure 3:**
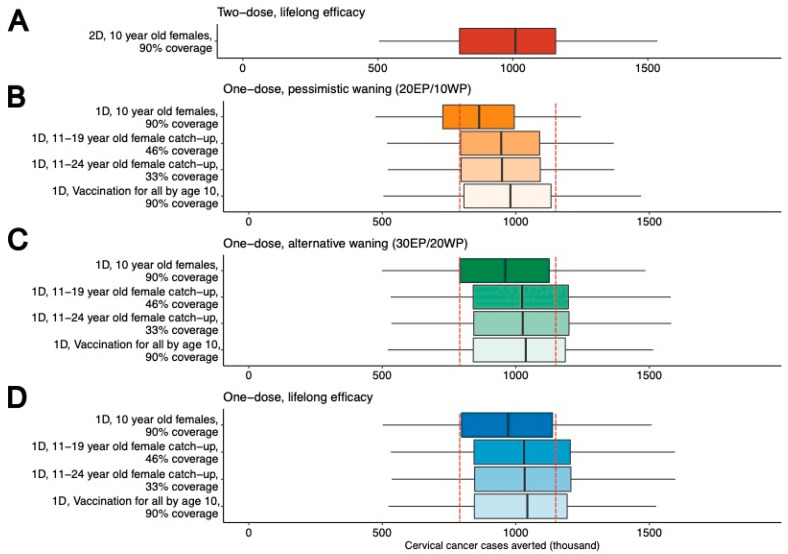
Projected cervical cancer cases averted (in thousands) over 100 years compared to a reference case of no vaccination. (**A**) Cervical cancer cases averted for a two-dose, 10-year-old girl vaccination strategy at 90% coverage. (**B**) Cervical cancer cases averted for one-dose strategies assuming a pessimistic waning scenario of 20 years of efficacy and 10 years of waning. (**C**) Cervical cancer cases averted for one-dose strategies assuming an alternative waning scenario of 30 years of efficacy and 20 years of waning. (**D**) Cervical cancer cases averted for one-dose strategies assuming lifelong efficacy. Dotted red lines represent the upper and lower quartiles of the two-dose 90% coverage results. Error bars are the 5th and 95th percentiles, vertical black horizontal lines are the median, and boxes are the 25th and 75th percentiles of 25 parameter sets. Abbreviations: 2D, two-dose; 1D, one-dose; EP, efficacy period; WP, waning period.

When comparing the five-year cost savings of all potential transitions from a two-dose to one-dose HVP vaccination schedule, the lowest cost saving would result from switching from a two-dose strategy at 70% coverage to one-dose strategy at 90% coverage. This is estimated to result in a cost saving of US$21.4 Million over five years (Supplementary Materials Section SIV). Using the mean estimate of the discounted total cost of vaccination of $7.77 per vaccination, the 5-year cost saving could cover an additional 2.75 million doses of the quadrivalent HPV vaccine. These vaccinations could be leveraged to expand HPV vaccination to other age groups and young boys in addition to vaccinating girls by age 10 at 90% coverage. Supplemental expanded HPV vaccination strategies that could be conducted with the 5-year cost savings include a single-dose catch-up HPV vaccination of girls and young women ages 11–19 at 46% coverage, catch-up HPV vaccination of girls and young women ages 11–24 at 33% coverage, and vaccination for all by age 10 at 90% coverage. Figure 3 and Table 2 show the impact of these additional strategies on cervical cancer incidence over 100 years. 

If we assume pessimistic waning of single-dose vaccine efficacy (20 EP/10 WP), a vaccination program of girls by age 10 at 90% coverage would reduce cervical cancer incidence by 61.5% (44.8–85.9) compared to the reference scenario of no vaccination. In comparison, the current two-dose strategy would reduce incidence by 73.1% (59.7–88.8). However, adding catch-up or vaccination for all by age 10 strategies alongside a single-dose vaccination of girls by age 10 could reduce cervical cancer incidence by up to 72.4% (55.5–88.7). This is similar to the incidence reduction that could be achieved with a two-dose vaccination strategy at a 90% coverage for girls by age 10, but the single-dose strategy paired with a catch-up or vaccination for all by age 10 strategy would require fewer vaccines to reach more individuals. 

Assuming the alternative waning scenario of 30 EP/20 WP and a single-dose, the percent reduction of cervical cancer incidence for vaccination of girls by age 10 would be similar between the one- and two-dose scenarios at 90% coverage. However, investing cost savings into an expanded single-dose vaccination strategy could reduce cervical cancer incidence even further to 75.9% (65.5–90.1), which is a greater reduction than that achieved with the current two-dose strategy at 90% coverage. Similarly, if we assume lifelong single-dose vaccine efficacy and supplemental vaccination, the percent reduction in cervical cancer incidence is predicted to reach 76.3% (66.6–90.2). 

**Figure 4:**
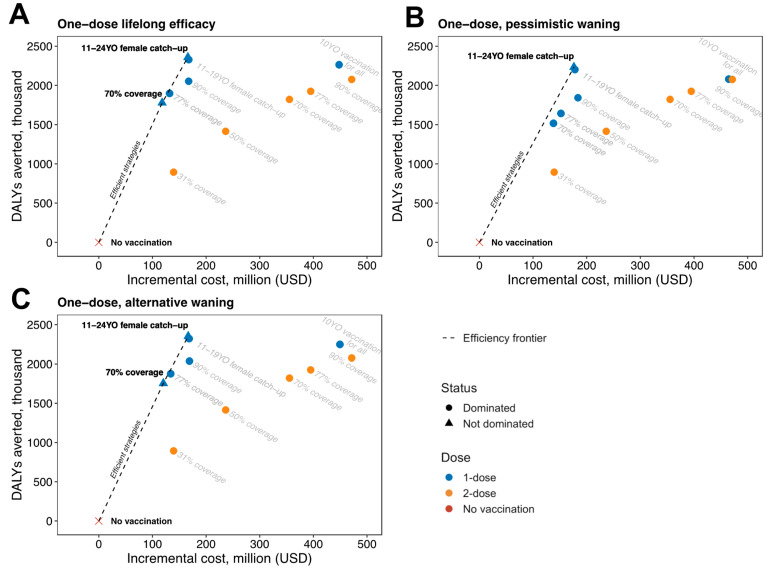
Cost-effectiveness analysis of vaccination of girls by age 10 at different coverage levels, or with additional catch-up or vaccination for all by age 10 strategies. Each panel considers different assumptions of one-dose HPV vaccine durability of (**A**) lifelong efficacy, (**B**) pessimistic waning of 20 years of efficacy and 10 years of waning, and (**C**) alternative waning of 30 years of efficacy and 20 years of waning. The graphs display the discounted incremental costs (*x*-axis; in 2023 USD) and DALYs averted (*y*-axis) over a 100-year time horizon compared to no vaccination. The cost-effectiveness of changing from one strategy to a more costly alternative strategy is calculated by taking the difference in cost and dividing it by the difference in DALYs of the two strategies. The dotted line represents the strategies that are efficient because they are more effective and either cost less or have a more attractive cost-effectiveness ratio than less effective options. The ICER is the reciprocal of the slope of the line connecting the two strategies under comparison. All points represent the median of 25 parameter sets.

The cost-effectiveness analysis of the lifelong efficacy, pessimistic waning, and alternative waning of one-dose HPV vaccination alongside catch-up or vaccination for all by age 10 strategies show that the two-dose strategies are consistently dominated (Figure 4). Comparatively, the single-dose HPV vaccination strategies alongside catch-up of 11–24-year-old girls and young women were consistently on the efficiency frontier, with ICERs ranging from US$75.47 (alternative waning) to US$86.16 (pessimistic waning). The single-dose HPV vaccination strategy at 70% coverage was also on the frontier for the lifelong efficacy and alternative waning assumptions. Vaccination for all by age 10 consistently averted the most cervical cancer cases, but at ICERS far off the efficiency frontier.

### 3.4. Sensitivity Analyses

When assessing ICERs of different scenarios compared to no additional vaccination, changes in health system delivery, treatment, and procurement cost had the greatest effect on the ICER across scenarios, while changes in the cost of supplies and screening had a lower effect on the ICER, followed by variations in Gavi contribution. For example, when comparing the two-dose HPV vaccination at 90% coverage to the no-vaccination scenario, a change in the cost of health system delivery resulted in a 13% change in the ICER while a change in the cost of supplies led to a 0.1% change in the ICER (Supplementary Materials Section SIV—Figure S17, Tables S22 and S23).

## 4. Discussion

HPV vaccination is a foundational strategy for cervical cancer elimination. Pairing robust HPV vaccination coverage with adequate and early cervical cancer screening and treatment services would significantly reduce the burden of cervical cancer in Kenya. While Kenya’s current HPV vaccination strategy focuses on vaccinating 10-year-old girls with two doses delivered in health facilities, leveraging a one-dose HPV strategy could catalyze progress towards cervical cancer elimination given the high efficacy of single-dose vaccination. This could allow the country to reach higher vaccination coverage at lower costs than a two-dose HPV vaccination strategy. Considering logistical and cost-related burdens with a two-dose schedule, switching to a one-dose schedule would accelerate progress towards increasing coverage of girls by age 10 and generate cost-savings that could be invested into catch-up vaccination for girls currently aged 11–24 or to expand HPV vaccination to young boys, which could further accelerate cervical cancer elimination efforts. Additionally, this switch would be strategic considering the anticipated graduation of Kenya from Gavi support in the next few years [11]. However, the impact of switching to a one-dose strategy needs to be evaluated, while also considering incorporating uncertainty around the durability of a single-dose. 

Focusing on vaccination of girls by age 10, we found that single-dose HPV vaccination scenarios at coverage levels from 70–90% or a two-dose scenario at 90% coverage would be the most efficient strategies, and these results are consistent regardless of potential waning durability. While a two-dose vaccination strategy at 90% coverage would lead to the greatest DALYs averted, it is also important to recognize the considerable costs required for a two-dose strategy relative to local cost-effectiveness standards. The incremental benefits of a two-dose strategy are further reduced if we observe an alternative one-dose waning scenario of 30 EP/20 WP or lifetime durability. The cost implications of a two-dose strategy are crucial to optimize health gains for available health resources. 

Although there is uncertainty around HPV vaccine durability, the demonstrated durability for single and multiple doses is comparable. Further, a supplemental vaccination for all by age 10 or catch-up strategy could mitigate the effects of potential one-dose waning efficacy. Our findings show that a single-dose schedule with a supplemental strategy could avert more cases of cervical cancer at a lower cost and more accelerated timeframe compared to a two-dose schedule. In this study, we found that by investing five-year cost savings of switching from a two- to one-dose schedule into a catch-up or vaccination for all by age 10 strategy, all two-dose strategies were dominated meaning that these strategies were both more expensive and resulted in the same or poorer health outcomes than alternative vaccination strategies. For the lifelong efficacy and alternative waning assumption, catch-up strategies resulted in better cervical cancer outcomes and more DALYs averted compared to the two-dose 90% coverage strategy and at significantly lower costs. In the scenario that one-dose durability follows the pessimistic waning assumption, catch-up of 11–24-year-old girls and young women would still result in the best health outcomes, with a 3.6% reduction in total DALYs compared to two-dose vaccination at 90% coverage, and a 33.3% reduction in total cost. The alternative waning and lifelong efficacy scenarios showed even greater reductions in total DALYs by 6.3% and 6.5%, respectively. 

An important context to our results is our assumptions around vaccine efficacy. We imposed a conservative constraint that two-dose efficacy is equal to or greater than one-dose efficacy for all 25 parameter sets run. However, the trial results show significant overlap in confidence intervals in efficacy, and no evidence suggests that two-dose vaccination strategies are superior to one-dose strategies. As a sensitivity analysis, we ran the one-dose 90% coverage of girls by age 10 with a lifelong vaccine efficacy scenario without imposing this bound on vaccine efficacy. The results shown in Supplementary Materials Section SIV, Figure S16 show much closer alignment in cervical cancer outcomes, with a one-dose schedule resulting in roughly 899 thousand cervical cancer cases averted while the two-dose schedule resulted in 900 thousand cases averted. 

As shown in Table 2 and Table 3, modeling potential waning showed that more pessimistic waning assumptions resulted in slightly worse projected health outcomes for single-dose strategies, but that even the most pessimistic plausible assumptions resulted in only modest decrements to health that still clearly defined the cost-effectiveness efficiency frontier. Our findings showed that at 90% coverage, single-dose HPV vaccination with lifelong efficacy resulted in a greater number of cervical cancer cases averted (971 thousand cases) compared to one-dose scenarios with waning efficacy where the single-dose (20 EP/10 WP) scenario had the lowest number of cervical cancer cases averted (862 thousand cases) but Figure 2 displays that this scenario none the less still comprises the efficiency frontier. Previous modelling studies have also explored the potential impact of single-dose HPV vaccination and waning efficacy in Kenya and other low- and middle-income countries (LMICs) [11,21,23]. One study by Bénard and colleagues modelled the impact of single-dose vaccination in four LMICs and found that single-dose strategies could prevent 69% to 94% of cervical cancer cases averted by a two-dose strategy [21]. This finding aligns with our results for Kenya, in which we expect a one-dose strategy to avert 85% (pessimistic waning) to 96% (lifelong efficacy) of the cervical cancer cases averted by an equivalent two-dose strategy. Another modelling study in India by Carvalho and colleagues showed similar estimates for the ICER of two-dose vaccination versus one-dose vaccination of US$3364 to US$15,530 for different vaccine efficacy and waning assumptions [23]. Finally, a recent modelling study in Kenya by Mwenda and colleagues compared the cost-effectiveness of different HPV vaccine products, and also found that switching to a single-dose strategy could save US$50 Million in ten years [11]. While strong evidence from modelling studies and trials supports the durable efficacy of single-dose HPV vaccination, future studies should assess the long-term (>20 years) efficacy of a single-dose, the efficacy of single-dose vaccination among people living with HIV, and economic evaluations of single-dose HPV vaccination accounting for HIV care costs given the association between HIV and HPV.

Our study explores the impact of using the five-year cost savings of switching from a two- to one-dose strategy to invest in an expanded catch-up or vaccination for all by age 10 program. It also expands on previous modelling studies with updated estimates of single-dose efficacy based on the KEN SHE Study, a trial conducted in Kenya. Further, we designed and conducted this study in close collaboration with the National Vaccines and Immunizations Program of Kenya. Model inputs, scenarios, and cost assumptions were decided with colleagues with deep expertise in the HPV vaccination program in Kenya. Our study is also the first single-dose modelling study that accounts for the HIV epidemic in Kenya and the bidirectional interaction between HIV and HPV. Our findings, while specific to Kenya’s context, could inform HPV vaccination in settings with similar vaccination delivery, screening and treatment costs as well as HIV and HPV epidemiologic profiles.

There are limitations of our study that could have impact on the findings. To estimate the impact of vaccination alone on cervical cancer outcomes, we maintained cervical cancer screening using VIA and treatment at the same coverage levels as present day. However, Kenya is advancing capacity for HPV DNA screening and treatment access. We expect expansion of screening to amplify the impact on cervical cancer cases and deaths averted. Future studies should also explore the impact of investing the cost savings of switching from a two- to one-dose vaccination strategy into expanded cervical cancer screening and treatment programs.

While the model accounts for differences in risk of HPV acquisition for women living with HIV, we do not model differences in vaccine doses, durability, or efficacy based on HIV status. To date, there have been no clinical trials that have studied single-dose efficacy for women living with HIV, so we kept vaccine parameters consistent regardless of HIV status. This emphasizes the need for studies that explore the impact of uncertainty of single-dose HPV schedules for women living with HIV. Since our focus was on HPV vaccination related costs, we did not include HIV-associated costs or disability weights in this study; rather, we chose to limit the study to evaluating costs associated with cervical cancer prevention, screening, and treatment. This study focused on a facility-based delivery of HPV vaccination, but future studies should also explore comparisons with school-based and mixed-models. Finally, our study does not account for the benefits of HPV vaccination on preventing other HPV-related cancers for both males and females, such as vaginal, vulvar, anal, oropharyngeal, and penile cancers, or the quality-of-life impacts of preventing genital warts from HPV types 6 and 11. As a result, the health outcomes from vaccination strategies reported in this study are likely underestimations. Additionally, given the simplified logistics for a single-dose HPV vaccination schedule both for providers and the community, we believe that it would result in higher uptake and catalyze progress in achieving high HPV vaccination coverage. Evidence from a recent implementation research study highlighted that most providers surveyed in Kenya thought that a single-dose schedule is acceptable, but future research should assess the acceptability of a single-dose schedule in the community and identify factors that might affect uptake in the community [50].

## 5. Conclusions

In conclusion, a single-dose HPV vaccination schedule, regardless of the uncertainty around waning vaccine efficacy, is more cost-effective than the current two-dose schedule, especially when the cost-savings of switching to one-dose vaccination are re-invested into a catch-up campaign. Catch-up of 11–24-year-old girls and young women alongside vaccination of girls by age 10 can overcome the possible effects of potential one-dose waning efficacy and could avert more cases of cervical cancer at a more accelerated timeframe and at a lower cost compared to a two-dose schedule. This could ultimately accelerate Kenya’s efforts towards cervical cancer elimination.

## Figures and Tables

**Table 2:** Projected cervical cancer incidence rates, cases averted, years to elimination (incidence below 4 per 100,000 women), and number of vaccines relative to a reference scenario of no vaccination from the years 2023 to 2123. Results are presented as a median and 90% confidence interval using 25 parameter sets.

Dose and Durability	Coverage	Additional Strategy ^c^	Cervical Cancer Incidence, per 100,000 ^a^	Percent Reduction in Incidence	Cumulative Cancer Cases Averted	Number of Vaccines (Millions)	Number of Vaccines to Avert 1 Cervical Cancer Case (Thousands)	Year Elimination Threshold Reached ^b^
One-dose, lifelong efficacy scenarios								
No vaccination	0%		26.0 (12.3–39.2)	Reference	Reference	0.0 (0.0–0.0)	Reference	X (X–X)
Two-dose	31%		14.6 (5.6–23.8)	33.4 (20.0–51.9)	460,516 (273,506–637,270)	77.1 (69.7–79.4)	16.0 (11.9–28.0)	X (X–X)
	50%		9.1 (3.0–15.8)	51.5 (36.4–73.7)	705,734 (396,958–1,003,420)	125.7 (113.7–129.6)	17.0 (12.3–31.5)	X (2093–X)
	70%		4.9 (1.8–9.1)	66.4 (50.0–85.1)	900,106 (471,208–1,317,820)	177 (160.0–182.5)	18.7 (13.2–37.4)	X (2080–X)
	77%		3.9 (1.7–7.3)	70.4 (53.8–86.7)	949,784 (485,919–1,406,610)	194.9 (176.2–201.0)	19.6 (13.6–39.9)	2117 (2078–X)
	90%		2.9 (1.6–4.9)	73.1 (59.7–88.8)	1,009,341 (504,392–1,536,007)	228.2 (206.4–235.3)	21.4 (14.6–45.0)	2094 (2076–X)
One-dose, lifelong efficacy	70%		5.2 (1.8–9.9)	62.7 (46.5–83.7)	851,996 (466,220–1,281,876)	88.5 (80.0–91.2)	9.9 (6.8–18.9)	X (2081–X)
	77%		4.1 (1.7–8.0)	67.1 (50.5–86.1)	899,829 (481,815–1,372,771)	97.5 (88.1–100.5)	10.2 (7.0–20.1)	X (2079–X)
	90%		3.1 (1.6–5.3)	72.5 (56.9–88.5)	971,797 (501,399–1,509,509)	114.1 (103.2–117.6)	11.0 (7.4–22.7)	2096 (2076–X)
One-dose, waning efficacy scenarios								
One-dose, 20 EP/20 WP	90%		3.7 (1.6–7.8)	66.7 (49.5–87.2)	908,713 (486,397–1,360,152)	114.1 (103.2–117.6)	11.8 (8.3–23.4)	2112 (2078–X)
One-dose, 25 EP/20 WP	90%		3.3 (1.6–6.2)	70.7 (53.4–87.9)	940,727 (494,742–1,444,153)	114.1 (103.2–117.6)	11.3 (7.8–23.0)	2101 (2077–X)
One-dose, 30 EP/20 WP	90%		3.1 (1.6–5.6)	72.1 (55.7–88.4)	960,471 (498,985–1,485,883)	114.1 (103.2–117.6)	11.1 (7.6–22.8)	2098 (2076–X)
One-dose, 20 EP/10 WP	90%		4.6 (1.7–10.2)	61.5 (44.8–85.9)	862,768 (474,837–1,243,891)	114.1 (103.2–117.6)	12.6 (9.0–23.9)	X (2080–X)
One-dose, 25 EP/10 WP	90%		3.5 (1.6–7.1)	68.3 (51.0–87.4)	922,133 (490,080–1,394,175)	114.1 (103.2–117.6)	11.6 (8.1–23.2)	2106 (2078–X)
One-dose, 30 EP/10 WP	90%		3.2 (1.6–5.8)	71.8 (54.8–88.2)	952,911 (497,402–1,469,637)	114.1 (103.2–117.6)	11.2 (7.6–22.8)	2099 (2076–X)
One-dose, using cost savings to invest in an additional vaccination strategy, lifelong and waning efficacy scenarios								
One-dose, lifelong efficacy	46%	CU girls age 11–19	3.0 (1.6–5.3)	75.7 (63.6–88.7)	1,031,065 (532,160–1,596,335)	114.1 (103.2–117.7)	10.3 (7.0–21.3)	2092 (2072–X)
	33%	CU girls age 11–24	3.0 (1.6–5.3)	75.6 (63.2–88.6)	1,033,237 (535,074–1,598,068)	114.1 (103.2–117.7)	10.3 (7.0–21.2)	2093 (2072–X)
	90%	Vaccination for all by age 10	2.7 (1.6–3.8)	76.3 (66.6–90.2)	1,058,653 (526,325–1,656,407)	221.9 (200.7–228.8)	19.7 (13.2–42.0)	2085 (2072–2105)
One-dose, 30 EP/20 WP	46%	CU girls age 11–19	3.1 (1.6–5.6)	75.5 (63.0–88.5)	1,024,003 (531,228–1,580,671)	114.1 (103.2–117.7)	10.4 (7.1–21.4)	2094 (2072–X)
	33%	CU girls age 11–24	3.1 (1.6–5.6)	75.4 (62.6–88.5)	1,026,159 (534,146–1,582,341)	114.1 (103.2–117.7)	10.4 (7.1–21.3)	2094 (2072–X)
	90%	Vaccination for all by age 10	2.7 (1.6–3.8)	75.9 (65.5–90.1)	1,052,968 (524,145–1,643,371)	221.9 (200.7–228.8)	19.8 (13.3–42.1)	2086 (2073–2107)
One-dose, 20 EP/10 WP	46%	CU girls age 11–19	4.5 (1.7–10.1)	66.3 (54.0–86.3)	945,419 (517,625–1,366,675)	114.1 (103.2–117.7)	11.5 (8.2–21.9)	X (2075–X)
	33%	CU girls age 11–24	4.5 (1.7–10.1)	66.2 (53.8–86.3)	948,401 (520,579–1,369,248)	114.1 (103.2–117.7)	11.5 (8.2–21.8)	X (2075–X)
	90%	Vaccination for all by age 10	3.0 (1.6–5.6)	72.4 (55.5–88.7)	979,871 (504,294–1,469,412)	221.9 (200.7–228.8)	21.2 (14.9–43.8)	2098 (2076–X)

Abbreviations: EP, efficacy period; WP, waning period; CU, catch-up. ^a^ Standardized to the 2000–2025 Standard Population distribution. ^b^ X is used to show that the elimination threshold was not reached during the 100-year time horizon. ^c^ All scenarios of additional strategies are HPV vaccination strategies that are pursued in addition to one-dose vaccination of girls by age 10 at a 90% coverage.

**Table 3:** Health and cost impact of vaccination strategies in Kenya. The analysis considers two possible decision scenarios—if the decision-maker considers only vaccination of girls by age 10, or if they would consider vaccination of girls by age 10 with additional strategies such as catch-up or vaccination for all by age 10.

Possible Scenario	One-Dose Durability	Strategy	Total Cost (Million 2023 USD); Median (90% CI) ^a^	Total DALYs (Thousand); Median (90% CI) ^a^	Incremental Cost (Million 2023 USD); Median (90% CI) ^a^	DALYs Averted (Thousand); Median (90% CI) ^a^	ICER ($ per DALY Averted); Median (90% CI) ^b^
Vaccination of girls by age 10 only	Lifelong	No vaccination	414.46 (230.62–606.00)	6467.38 (3526.16–9180.60)	-	-	-
		1-dose, 70% coverage	532.50 (411.14–676.31)	4689.55 (2634.16–6757.53)	122.38 (70.8–181.01)	1625.58 (892–2423.09)	74.60 (29.41–203.58)
		1-dose, 77% coverage	546.33 (433.32–687.94)	4569.15 (2592.00–6568.43)	16.84 (11.63–22.18)	111.46 (42.16–189.1)	142.78 (61.72–527.97)
		2-dose, 31% coverage ^c^	553.82 (403.54–730.33)	5573.07 (3039.92–8036.84)	-	-	Dominated
		1-dose, 90% coverage	581.87 (475.74–713.27)	4413.62 (2531.41–6270.33)	34.14 (25.33–42.58)	171.73 (60.6–298.11)	197.44 (85.15–702.24)
		2-dose, 50% coverage ^c^	650.86 (522.48–814.85)	5052.00 (2791.19–7317.65)	-	-	Dominated
		2-dose, 70% coverage ^c^	769.83 (654.07–912.34)	4645.83 (2620.47–6680.84)	-	-	Dominated
		2-dose, 77% coverage ^c^	809.38 (701.45–948.99)	4541.81 (2579.57–6493.85)	-	-	Dominated
		2-dose, 90% coverage ^c^	885.85 (790.65–1021.16)	4391.11 (2520.83–6205.23)	313.33 (284–325.23)	49.64 (6.16–115.54)	6508.80 (2527.55–51,541.03)
	Alternative waning (30 EP/20 WP)	1-dose, 70% coverage	534.73 (411.73–680.77)	4712.72 (2640.58–6805.54)	124.02 (75.71–181.1)	1604.99 (885.57–2375.06)	76.43 (31.67–205.15)
		1-dose, 77% coverage	548.46 (433.87–692.20)	4590.55 (2598.04–6614.31)	16.71 (11.42–22.14)	112.74 (42.55–191.24)	140.23 (59.94–522.43)
		1-dose, 90% coverage	583.35 (476.25–716.95)	4430.33 (2536.90–6310.30)	33.9 (24.76–42.49)	174.06 (61.14–304.01)	191.95 (81.65–695.31)
		2-dose, 90% coverage	885.85 (790.65–1021.16)	4391.11 (2520.83–6205.23)	312.05 (283.11–324.95)	68.63 (15.86–146.08)	4736.59 (2053.19–20,027.37)
	Pessimistic waning (20 EP/10 WP)	1-dose, 70% coverage	552.53 (417.44–714.83)	4949.92 (2713.50–7235.11)	141.85 (120.03–187)	1370.93 (807.36–1827.14)	102.11 (66.41–232.15)
		1-dose, 77% coverage	566.41 (439.30–726.91)	4824.51 (2667.34–7051.80)	16.58 (13.32–23.97)	115.94 (46.16–183.31)	137.18 (66.12–475.72)
		1-dose, 90% coverage	598.16 (481.21–751.26)	4624.61 (2600.13–6741.83)	32.67 (24.36–41.91)	184.17 (67.21–309.98)	168.55 (78.8–626.06)
		2-dose, 90% coverage	885.85 (790.65–1021.16)	4391.11 (2520.83–6205.23)	291.13 (257.27–316.5)	276.02 (79.3–536.6)	1087.49 (503.03–3908.66)
Vaccination of girls by age 10 + additional vaccination strategies	Lifelong	1-dose, 70% coverage	532.50 (411.14–676.31)	4689.55 (2634.16–6757.53)	128.92 (99.49–183.61)	1579.44 (839.55–2061.36)	85.11 (48.29–236.32)
		1-dose, 11–24 YO catch-up of girls ^d^	580.44 (482.29–701.29)	4108.85 (2356.00–5833.02)	58.75 (39.37–135.52)	603.47 (278.16–3347.58)	78.73 (28.64–256.28)
		1-dose, 11–19 YO catch-up of girls ^e^	582.19 (483.80–703.48)	4138.94 (2378.70–5864.55)	-	-	Dominated
		1-dose, Vaccination for all by age 10 ^f^	862.35 (771.21–983.84)	4203.98 (2438.65–5868.82)	279.07 (279.07–279.07)	120.51 (120.51–120.51)	Weakly Dominated
	Alternative waning (30 EP/20 WP)	1-dose, 70% coverage	534.73 (411.73–680.77)	4712.72 (2640.58–6805.54)	142.54 (112.35–192.57)	1402.52 (720.28–1889.22)	97.89 (59.68–348.11)
		1-dose, 11–24 YO catch-up of girls ^d^	581.05 (482.42–703.09)	4115.01 (2357.46–5852.30)	69.23 (44.66–159.32)	636.39 (283.12–3328.31)	75.47 (29.35–250.21)
		1-dose, 11–19 YO catch-up of girls ^e^	582.80 (483.94–705.29)	4145.09 (2380.17–5883.79)	-	-	Dominated
		1-dose, Vaccination for all by age 10 ^f^	863.94 (771.74–986.70)	4219.35 (2444.50–5900.54)	279.81 (279.81–279.81)	110.68 (110.68–110.68)	Weakly Dominated
	Pessimistic waning (20 EP/10 WP)	1-dose, 70% coverage	534.73 (411.73–680.77)	4712.72 (2640.58–6805.54)	-	-	-
		1-dose, 11–24 YO catch-up of girls ^d^	590.69 (484.67–729.11)	4233.94 (2385.33–6174.27)	182.22 (126.04–254.05)	2066.75 (1140.82–3006.33)	86.16 (41.12–223.31)
		1-dose, 11–19 YO catch-up of girls ^e^	592.52 (486.23–731.61)	4265.01 (2408.43–6209.01)	-	-	Dominated
		1-dose, Vaccination for all by age 10 ^f^	879.21 (776.54–1016.81)	4387.00 (2505.77–6278.66)	285.63 (285.63–285.63)	30.59 (30.59–30.59)	Weakly Dominated

Abbreviations: EP, efficacy period; WP, waning period; YO, year-old. ^a^ Results are summarized as a median of all 25 parameter sets. ^b^ ICERs are calculated for each parameter set, and results are summarized as the median ICER of the 25 parameter sets. ^c^ Dominated two-dose strategies were included as part of the cost-effectiveness analysis but are not reported here for brevity since the results are the same as the vaccination of girls by age 10 lifelong efficacy results. Please see Supplementary Materials Section SIV dii for an expanded table of these results. ^d^ 11–24-year-old catch-up of girls and young women with one-dose at a coverage of 33%, in addition to vaccination of girls by age 10 at a coverage of 90%. ^e^ 11–19-year-old catch-up of girls and young women with one-dose at a coverage of 46%, in addition to vaccination of girls by age 10 at a coverage of 90%. ^f^ Vaccination of boys and girls by age 10 at a coverage of 90%.

## Data Availability

The data that support the findings presented in the study are included in the article/Supplementary Materials, further inquiries can be directed to the corresponding author.

## Supplementary Materials

The following supporting information are available online https://www.mdpi.com/article/10.3390/vaccines12111248/s1, Section SI: Model overview; Section SII: Modules and parameter values; Section SIII: Calibration and validation; Section SIV: Additional results; Section SV: Differential equations; Section SVI: Reporting; Section SVII: References.
