# Supplementary material for: The Potential Impact of a Single-Dose HPV Vaccination Schedule on Cervical Cancer Outcomes in Kenya: A Mathematical Modelling and Health Economic Analysis
Source: Vaccines (Basel). 2024 Nov 1;12(11):1248. doi: 10.3390/vaccines12111248 (PMC11598770; doi:10.3390/vaccines12111248)
Supplement: Supplementary file 1 [file vaccines-12-01248-s001.zip › vaccines-3268153-supplementary.pdf]

Supplementary Materials

Table of Contents

I. Model overview 2
II. Modules and parameter values 4
a. Demography 4
b. Sexual behavior 5
c. Natural history 6
i. HIV 6
ii. HPV and cervical cancer 7
d. Historical interventions 10
i. HIV antiretroviral therapy (ART) 10
ii. Condoms 11
iii. Circumcision 11
iv. HPV vaccination 12
v. HPV screening and treatment 14
III. Calibration and validation 15
a. Calibration 15
b. Validation 19
c. Comparing model output to data 19
IV. Additional results 23
a. Sensitivity Analysis: Changing the Screening Technology 23
b. Sensitivity Analysis: Unbounding Single Dose Efficacy 24
c. Additional Modelling Results 25
d. Additional Economic Results 25
V. Differential equations 36
a. Demography 37
b. Sexual Behavior 38
c. Transmission Probabilities 39
d. Natural History and Interventions 41
VI. Reporting 46
a. HPV-FRAME Checklist 47

b. CHEERS 2022 Checklist 50
VII. References.. 52

I. Model overview

For this modeling study, we used a deterministic, compartmental model parameterized to represent transmission and progression of HIV and oncogenic human papillomavirus (or high-risk HPV (hrHPV)) in Kenya, which has an HIV prevalence of 4.5% in 2019.[1] The model used for this study is based on a previously published model of HPV vaccination in Kenya, and has been expanded to include symptomatic detection of cervical cancer and treatment by modalities in addition to hysterectomy.[2] The primary objective of this modeling study is to evaluate and predict population-level impact of one- and two- dose HPV vaccination on cervical cancer outcomes, while taking into account the changing HIV epidemiology and scaling-up of HIV prevention in Kenya.

Because the data we use to inform the model are not stratified by gender, our model population represents a primarily heterosexual population in Kenya. Men and women infected with hrHPV may clear the infection, and infections in women may progress through stages of precancerous lesions to cervical cancer (Figure S1). HIV progression is tracked by CD4+ T-cell (CD4) count and HIV RNA concentration (viral load), and infected individuals may achieve viral suppression with antiretroviral therapy (ART) beginning in 2005 (Figure S1). A key feature of our model is representation of the bidirectional interaction between HIV and HPV, whereby HIV infection increases the probability of HPV acquisition and the rate of disease progression and HPV infection increases the probability of HIV acquisition. We calibrate the model to fit to HIV, HPV, and cervical cancer epidemiology in Kenya.

Model dynamics are governed by a system of differential equations that are solved in MATLAB using a 4th-order Runge-Kutta numerical method. The model simulates events in discrete time with two-month intervals. At each time step, differential equations are evaluated to estimate population demographics and the number of persons in each infection, disease, or treatment compartment for the following time step. The dynamic nature of our transmission model captures population-level effects such as herd immunity.

This work was facilitated through the use of advanced computational, storage, and networking infrastructure provided by ERISTwo at Mass General Brigham.

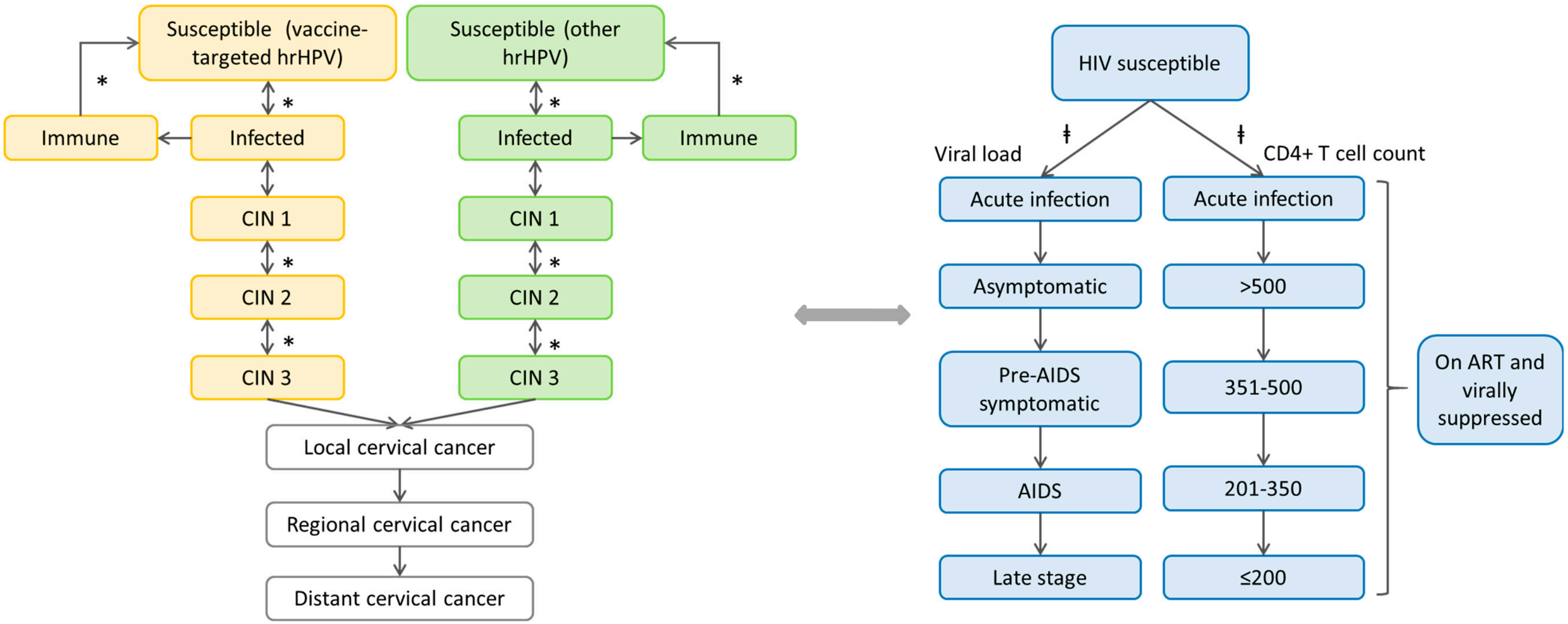

Figure S1. Model schematic illustrating HPV progression and HIV progression. We model HPV infection with high-risk HPV (hrHPV) types targeted by HPV vaccines (HPV 16, 18, 31, 33, 45, 52, and 58) and other high risk, non-vaccine targeted types (HPV 35, 39, 51, 56, 59 and 68). Arrows with * indicate processes that are affected by HIV infection, and arrows with ɫ indicated processes affected by HPV infection.

II. Modules and parameter values

a. Demography

We model a dynamic, open population of males and females aged 0 to 79 in Kenya in 5-year age groups. To allow HPV transmission dynamics and cervical cancer to equilibrate before introducing HIV in 1980, we initialize the model in 1925. The sex- and age-specific distribution of our initial population in 1925 is based data from United Nations Population Division (UNPD), which estimates Kenya population by sex and age starting in 1950.[3] We fit exponential distributions for each sex and age group to extrapolate backwards to 1925 (Table S1).

At each two-month time step, the model calculates the number of births and deaths based on fertility and mortality rate inputs. The initial fertility rates are based on UNPD-estimated rates for 1960.[3] We then scale down fertility rates to produce population distributions consistent with UNPD estimates from 1950-2020 as well as Kenya Census data from 2009 and 2019.[3-5] We assume fertility rates for women living with HIV (WLHIV) with CD4 count of <500 cells/mm^3 are 0.41-0.58 of women without HIV and the rates for WLHIV on ART or had CD4 >500 cells/mm3 are the same as women without HIV (Table S2).[6,7] We further scale down fertility rates after 2020 to fit projected population estimates.

To age the population, one-fifth of each compartment moves to the next age group annually. Persons leave the population due to death or aging past 79. To model deaths, we apply age- and sex-specific mortality rates estimated by UNPD (Table S3).[3] We assume linear changes in rates between 1950, 1985, 2010, and 2020, which are the years for which rates are available. After initializing HIV in 1980, we model background and HIV-specific mortality separately. To estimate background mortality rates not related to HIV, we subtract HIV-specific mortality[8,9] from UNPD-estimated mortality rates.

Table S1. Initial population size. The population distribution by sex and 5-year age groups is projected backward from UNPD estimates for Kenya in 1950.

Age Group	Initial Population Size		Source
	Male	Female	
**0 – 4**	237966	240166	
**5 – 9**	143534	145291	
**10 – 14**	110242	110999	
**15 – 19**	99096	97339	
**20 – 24**	91121	86235	
**25 – 29**	84685	75947	
**30 – 39**	80170	68470	
**35 – 39**	78914	63502	UNPD[3]
**40 – 44**	79282	61809	
**45 – 49**	76761	63348	
**50 – 54**	68455	62295	
**55 – 59**	52798	53896	
**60 – 64**	39427	45400	
**65 – 69**	27875	36647	
**70 – 74**	16553	25006	
**75 – 79**	7905	14683	
**TOTAL**	**1294783**	**1251034**	

Table S2. Baseline fertility rates per 1000 women by age. These fertility rates are applied from 1925-1970, then scaled down thereafter. Age groups not shown in this table have fertility rates of 0.

Age Group	HIV Uninfected	Source
**15 – 19**	0.182	
**20 – 24**	0.379	
**25 – 29**	0.365	
**30 – 34**	0.306	UNPD[3]
**35 – 39**	0.219	
**40 – 44**	0.119	
**45 – 49**	0.042	

Table S3. UNPD-estimated age- and sex-specific background mortality rates for 1950, 1985, 2000, and 2020.[3] We assume linear changes in background mortality between the years. These rates do not include deaths caused by HIV, which are estimated separately.

	1925-1950	By 1985	By 2000	By 2020	Source

Age Group	Male	Female	Male	Female	Male	Female	Male	Female	
**0 – 4**	0.103	0.088	0.047	0.040	0.071	0.062	0.036	0.027	
**5 – 9**	0.009	0.009	0.003	0.002	0.004	0.003	0.001	0.001	
**10 – 14**	0.005	0.005	0.002	0.002	0.003	0.002	0.001	0.001	
**15 – 19**	0.006	0.006	0.003	0.002	0.004	0.003	0.001	0.001	
**20 – 24**	0.009	0.007	0.004	0.003	0.006	0.005	0.002	0.001	
**25 – 29**	0.010	0.008	0.005	0.004	0.009	0.009	0.003	0.002	
**30 – 34**	0.011	0.009	0.006	0.005	0.012	0.012	0.004	0.003	
**35 – 39**	0.012	0.010	0.007	0.006	0.016	0.016	0.005	0.004	UNPD[3]
**40 – 44**	0.014	0.012	0.009	0.007	0.019	0.017	0.007	0.005	
**45 – 49**	0.016	0.013	0.011	0.008	0.023	0.018	0.009	0.006	
**50 – 54**	0.020	0.016	0.015	0.010	0.027	0.019	0.012	0.008	
**55 – 59**	0.025	0.020	0.019	0.013	0.033	0.022	0.016	0.010	
**60 - 64**	0.035	0.030	0.028	0.020	0.044	0.028	0.024	0.015	
**65- 69**	0.050	0.045	0.042	0.032	0.061	0.040	0.036	0.024	
**70 - 74**	0.075	0.071	0.066	0.054	0.091	0.061	0.056	0.039	
**75 - 79**	0.116	0.110	0.108	0.091	0.138	0.096	0.089	0.066	

b. Sexual behavior

In our model, sexual activity begins in the 10-14 age group. In each sexually active age group, we divide the population into low-, medium-, or high-risk groups with variable rates of partnership formation. The distribution of these risk groups by sex and age is informed by self-reported sexual behaviors data from 15-54 year old respondents in the 2014 Kenya Demographic and Health Survey (DHS),[10] and is calculated as the percentage of the cohort who reported 0-1 partner (low risk), 2-4 partners (moderate risk), or ≥5 partners (high risk) in the past year. We then calibrate risk group distribution for ages 15-54 to fit to observed HIV and HPV data. Individuals aged 10-14 are assumed to be predominately low risk, while the risk distribution for ages 55 and older is extrapolated from the 50-54 age group (Table S4).

Similarly, while the initial sexual partnership inputs are informed by the 2014 Kenya DHS data,[10] we calibrate the yearly partner change rates in each risk group by sex and age to fit to observed disease data (Table S5). This approach partially compensates for reporting biases and the lack of partnership concurrency in our model. Our compartmental model structure is not equipped to represent concurrent partnerships. Therefore, using the number of sex partners as reported would underestimate the rate of HPV and HIV transmission.

Using methods similar to other models, patterns of sexual contact in our model are characterized by age and sexual risk groups.[11] The degree of mixing is defined by the parameter, ϵ (ϵ_α for mixing by age and ϵ_r for mixing by risk group), which ranges from 0, indicating completely assortative (like-with-like), to 1, indicating random mixing that is proportional to compartment size. Based on studies that show that Kenyan women are on average 4-7 years younger than their male partners,[12] we assume an age mixing matrix with $\epsilon_\alpha = 0.2$. For mixing by risk group, we assume $\epsilon_r = 0.3$.

Because we model heterosexual contact, the modeled number of partnerships must be the same for men and women. However, the observed data we used to inform these parameters are subject to selection and response biases, resulting in imbalances. To correct this, we adjust contact rates such that the number of partners for men equals the number of partners for women.

Table S4. Proportion of individuals in low-, medium-, and high-risk groups by sex and age. The risk group distribution for those older than 55 is extrapolated from the 50-54 age group.

	Males			Females			Source
**Age Group**	**Low-Risk**	**Medium-Risk**	**High-Risk**	**Low-Risk**	**Medium-Risk**	**High-Risk**	
**10 – 14**	0.972	0.026	0.002	0.972	0.026	0.002	2014 Kenya DHS[10] / calibrated

**15 – 19**	0.779	0.216	0.004	0.757	0.234	0.009	
**20 – 24**	0.564	0.424	0.012	0.595	0.377	0.028	
**25 – 29**	0.611	0.365	0.023	0.635	0.354	0.010	
**30 – 34**	0.656	0.331	0.013	0.689	0.301	0.010	
**35 – 39**	0.717	0.275	0.008	0.730	0.263	0.007	
**40 – 44**	0.732	0.262	0.006	0.781	0.213	0.005	
**45 – 49**	0.807	0.188	0.005	0.826	0.169	0.005	
**50 – 54**	0.891	0.106	0.004	0.891	0.106	0.004	
**55 – 79**	0.891	0.106	0.004	0.891	0.106	0.004	

Table S5. Average number of partners in each risk group by sex and age. Estimates for those older than 55 is extrapolated from the 55-60 age group.

	**Males**			**Females**			**Source**
**Age Group**	**Low-Risk**	**Medium-Risk**	**High-Risk**	**Low-Risk**	**Medium-Risk**	**High-Risk**	
**10 – 14**	0.03	0.00	0.00	0.03	0.01	0.00	
**15 – 19**	0.23	2.45	5.41	0.50	2.45	6.41	
**20 – 24**	0.87	2.58	9.78	0.87	2.58	11.08	
**25 – 29**	0.87	2.33	11.10	0.90	2.33	10.15	
**30 – 34**	0.93	2.11	10.15	0.95	2.11	9.76	2014 Kenya DHS[10] / calibrated
**35 – 39**	0.93	2.08	9.67	0.93	2.08	9.67	
**40 – 44**	0.93	2.06	8.28	0.93	2.06	8.28	
**45 – 49**	0.93	2.00	7.35	0.93	2.00	7.35	
**50 – 54**	0.92	1.90	7.03	0.92	1.90	7.03	
**55 – 79**	0.92	1.85	2.50	0.92	1.85	2.50	

c. Natural history

i. HIV

HIV begins in 1980 in our model with an initial prevalence of 0.5% in ages 15-54 and 0.2% in other age groups. HIV infection occurs either through mother-to-child transmission or heterosexual contact. We model mother-to-child transmission rates that decrease over time to reflect improvements in services for pregnant women living with HIV (Table S6).[13,14] The force of HIV infection is estimated as a function of sexual mixing (by age and sexual risk group), HIV prevalence in the opposite sex, male circumcision, HIV viral load, and, among women, their current HPV infection status. The risk of HIV transmission is highest during the acute stage of infection. Risk decreases during the asymptomatic phase of HIV infection before increasing in the pre-AIDS symptomatic and AIDS stages.[15-18] We base HIV transmission per act in the asymptomatic stage on literature[19] and apply risk multipliers across the other stages of infection (Tables S6).[15-18,20] We assume male-to-female transmission probability is equal to female-to male transmission probability across all viral load stages. As a proxy for decreased sexual activity due to advanced disease during late-stage HIV, we reduce HIV per-act transmission to be 10% of the AIDS rate.[18] Based on the evidence that HPV, like other sexually transmitted infections, increased the risk of HIV acquisition, we assume that women with an HPV infection have between 1.4-2.2 times higher risk of acquiring HIV.[21,22] Eleven observational studies have been done to evaluate the association between HPV and HIV acquisition in women.[23-33] In five of the observational studies, HPV infection was ascertained one or more years before HIV seroconversion.[23,26,27,31,32] Because most HPV infections are transient and clear without intervention in less than one year,[34] measurement bias related to the exposure is likely in these studies. One study did not adjust for any confounders despite a high prevalence of herpes simplex virus 2 among its study participants.[30] Of the remaining five studies, four found that HPV infections significantly increased HIV risk[24,25,28,33] while one found no association.[29] All but two of the observational studies[32,33] were included in at least one of two meta-analyses summarizing the association between HPV infection and HIV acquisition.[21,22]

The transition rates between CD4 count stages and viral load stages are based on literature describing the average duration in each CD4 and viral load stage by sex and age (Tables S7).[16,35-37] Starting in 2005, we model individuals with ART-mediated

viral suppression. HIV-associated mortality rates with untreated HIV are estimated from studies of untreated persons living with HIV and depend on CD4 cell count and age (Table S8).[9,38,39] As a result of these combination of disease progression and mortality rates, untreated women have a longer average life expectancy than untreated men. In addition, children under five have the highest HIV-specific mortality, and adults >50 years have HIV mortality rates two times that of persons aged 5-49.[8,40] HIV-associated mortality for people on ART treatment is relative to background mortality, and decreases over time to reflect improvement in baseline health among persons initiating treatment.[41-43] From 2004 to 2011, HIV-associated mortality among people with HIV on ART is 1.5 times the background rate. This rate decreases to 1.4, 1.25, and 1.15 times the background mortality rate in 2011, 2015, and 2016, respectively.

Table S6. Annual HIV transmission rates by route of transmission. To capture uncertainty related to the values of select influential parameters, we let the probability of HIV transmission via sexual contact vary within a range.

Route	Value	Risk multipliers by viral load stages					Reference
		Acute	Asymptomatic	Pre-AIDS symptomatic	AIDS	Late-stage	
**Mother to child**							
**Before 2004**	0.42						
**By 2007**	0.32	1.0	1.0	1.0	1.0	1.0	13,14
**By 2013**	0.20						
**Sexual contact**	0.0008-0.0012	9.0	1.0	2.5	7.0	0.7	15-20

Table S7. Average duration of time (in years) spent in each CD4 stage and viral load stage with untreated HIV by sex and age. However, the actual time spent in each state is lower due to the effects of background and disease-specific mortality rates. We estimate time in the asymptomatic stage such that the total time spent in all viral load stages matches the total time spent in CD4 stages.

HIV states	Males			Females			Reference
	0-4	5-49	50-79	0-4	5-49	50-79	
**CD4 counts**							
**Acute**	0.25	0.25	0.25	0.25	0.25	0.25	
**≥501**	0.25	0.25	0.25	0.93	0.93	0.29	
**351-500**	3.56	3.56	2.85	3.71	3.71	3.34	35,37
**201-350**	4.67	4.67	4.51	4.68	4.68	4.23	
**≤200**	2.13	3.70	1.85	2.13	3.70	1.85	
**Viral load**							
**Acute**	0.25	0.25	0.25	0.25	0.25	0.25	
**Asymptomatic**	5.87	6.60	3.88	5.87	7.44	4.13	
**Pre-AIDS symptomatic**	4.00	4.00	4.00	4.00	4.00	4.00	16,36
**AIDS**	0.75	0.75	0.75	0.75	0.75	0.75	
**Late-stage**	0.83	0.83	0.83	0.83	0.83	0.83	

Table S8. HIV-specific mortality by CD4 cell count and age.

Age Group	Acute	CD4 ≥500	CD4 350-500	CD4 200-350	CD4 ≤200	Reference
**0 – 4**	0	0.4700	0.4700	0.4700	0.4700	
**5 – 49**	0	0.0035	0.0255	0.0455	0.2655	8,9,38-40
**50 – 79**	0	0.0071	0.0511	0.0911	0.5311	

ii. HPV and cervical cancer

The model simulation begins in 1925 with an initial HPV prevalence of 20% among 15-44-year-old men and women. Because we are primarily interested in cervical cancer outcomes, our model represents infection with the HPV types classified as high risk, or oncogenic, by the International Agency for Research on Cancer.[44] Infections are due to either vaccine-targeted hrHPV types (HPV 16, 18, 31, 33, 45, 52, and 58) or non-vaccine-targeted hrHPV types (HPV 35, 39, 51, 56, 59 and 68). Per-coital HPV transmission probability is based on literature[45] then calibrated (Table S9). We assume that transmission probability of non-vaccine-targeted HPV type is higher than vaccine-targeted HPV type to reproduce type distribution of HPV infections in sub-Saharan Africa.[46,47] HPV transmission among women with regional or distant cervical cancer is reduced by 50%. We set that male-to-female and female-to-male transmission probabilities to be equal. However, we assume that men do not develop natural immunity.[48] Women develop partial immunity against reinfection with the same HPV type group (vaccine-type or non-vaccine type) that wanes at an annual rate of 0.024 (Table S9).[49] In our model, women with persistent HPV infection can progress to precancerous lesions (represented as cervical intraepithelial neoplasia (CIN) grades 1, 2, or 3) and cervical cancer

(categorized as local, regional, or distant) as shown in Figure S1. CIN 1, 2, and 3 can regress and HPV infection can clear naturally. CIN progression and regression rates were based on data previously described by Tan et al. (Supplement Table S23).[50] The transition rates were defined separately for nonavalent vaccine-targeted hrHPV types and other hrHPV types. The original model used by Tan et al included additional transitional states. We reweighted transitions by types and consolidated transitions to better match our current HPV type grouping and natural history structure. For reweighting purposes, we assumed the type distribution of CIN2 to be the average of the type distributions for low-grade and high-grade lesions. After the reweighting, transition rates for each infection type (vaccine-targeted or non-vaccine-targeted) were manually calibrated (Table S9).

Individuals with HIV have higher rates of HPV acquisition, immunity waning, and disease progression, and lower rates of HPV clearance and CIN regression.[51] The effect size of HIV on HPV natural history is inversely correlated with CD4 cell count and is represented by risk multipliers on HPV infection acquisition and clearance and CIN progression or regression in women without HIV. The risk multipliers are initially based on literature then calibrated to fit to observed data (Table S9). Women on ART have lower risk of HPV infection and cervical precancer lesions compared to untreated women living with HIV and similar HPV prevalence compared to women without HIV.[52,53] However, sub-Saharan Africa women living with HIV continue to have elevated cancer incidence rates despite being on ART.[54,55] Based on these data, we assume women on treatment and virally suppressed have HPV acquisition and disease progression rates comparable to women without HIV, but HPV clearance, disease regression rates, and cervical cancer-associated mortality are equivalent to untreated women with high (>500) CD4 count. WLHIV also have higher cervical cancer-associated mortality rates than women without HIV,[56] with the mortality rates increasing with decreasing CD4 count (Table S10).[57] The effect of HIV on mortality decreases as cervical cancer progresses.[57]

Because the model does not track infection duration, we use age multipliers as proxies for HPV persistence. The multipliers are applied to both HPV types and scaled up linearly across each age grouping (Table S11). The age multipliers are based on those previously described by Tan et al., 2018 in Supplement Table S25.[50] The multiplier values are the average of the vaccine-type and non-vaccine-type relative risks after rate adjustment.

Table S9. HPV-related transition rates per year. Multipliers are relative risks of transitioning between HPV states for women with HIV compared to women without HIV, and depended on CD4 count and ART status. To capture uncertainty related to the values of select influential parameters, we let the probability of vaccine-targeted HPV transmission vary within a range.

Parameter description	Without HIV		Multipliers with HIV					
	Value	Reference	On ART	CD4 ≥500	CD4 350-500	CD4 200-350	CD4 ≤200	Reference
**Per-partner transmission probability**								
**Vaccine-targeted**	0.008-0.014	[45], calibrated	1	2.14	2.39	2.54	2.78	[22,58], calibrated
**Non-vaccine-targeted**	0.016	Calibrated						
**HPV to CIN 1**								
**Vaccine-targeted**	0.586	Calibrated	NA	NA	NA	NA	NA	
**Non-vaccine-targeted**	0.266	Calibrated						
**CIN 1 to CIN 2**								
**Vaccine-targeted**	0.182	Calibrated	1	1.05	1.47	1.89	2.31	Calibrated
**Non-vaccine-targeted**	0.065	Calibrated						
**CIN 2 to CIN 3**								
**Vaccine-targeted**	0.169	Calibrated	1	1	1.1	1.2	1.4	Calibrated
**Non-vaccine-targeted**	0.076	Calibrated						
**CIN3 to local cancer**								
**Vaccine-targeted**	0.002	Calibrated	NA	NA	NA	NA	NA	
**Non-vaccine-targeted**	0.001	Calibrated						
**Local to regional cancer**	0.225	[59]						
**Regional to distant cancer**	0.450	[59]						
**HPV to immune in women**								
**Vaccine-targeted**	1.772	Calibrated	0.6	0.6	0.55	0.45	0.30	Calibrated
**Non-vaccine-targeted**	1.768	Calibrated						
**HPV to susceptible in men**								
**Vaccine-targeted**	1.238	Calibrated	0.6	0.6	0.55	0.45	0.30	Calibrated
**Non-vaccine-targeted**	1.241	Calibrated						
**CIN 1 to HPV**								
**Vaccine-targeted**	0.360	Calibrated	0.6	0.6	0.55	0.45	0.30	Calibrated
**Non-vaccine-targeted**	0.423	Calibrated						
**CIN2 to CIN 1**								
**Vaccine-targeted**	0.494	Calibrated	0.93	0.93	0.853	0.698	0.465	Calibrated
**Non-vaccine-targeted**	0.301	Calibrated						
**CIN 3 to CIN 2**								
**Vaccine-targeted**	0.102	Calibrated	1.02	1.02	0.935	0.765	0.51	Calibrated
**Non-vaccine-targeted**	0.101	Calibrated						
**Natural immunity waning**	0.024	[49]	1.42	1.42	1.57	1.97	2.83	Calibrated
**Natural immunity protection**	70%	[60]	NA	NA	NA	NA	NA	
**Annual probability of getting diagnosed***								
**Local**	0.007-0.011	[61,62], Calibrated	NA	NA	NA	NA	NA	Calibrated
**Regional**	0.286-0.417	[61,62], Calibrated						
**Distant**	0.728-0.978	[61,62], Calibrated						

* In a process separate from routine screening

Table S10. Cervical cancer-associated mortality by cancer stage, treatment status, and CD4 cell count among women living with HIV.

	Untreated Cervical Cancer			Treated Cervical Cancer			
	Local	Regional	Distant	Local	Regional	Distant	Reference
**HIV uninfected**	0.0994	0.2239	0.5945	0.0692	0.1867	0.5744	56,57,63
**HIV-positive on ART**	0.3298	0.3604	0.5945	0.2296	0.3005	0.5744	
**CD4 ≥500**	0.3298	0.3604	0.5945	0.2296	0.3005	0.5744	
**CD4 350-500**	0.4004	0.4376	0.5945	0.2788	0.3649	0.5744	
**CD4 200-350**	0.4861	0.5313	0.5945	0.3384	0.4430	0.5744	
**CD4 ≤200**	0.7148	0.7813	0.5945	0.4977	0.6514	0.5744	

Table S11. Age multipliers on transition rates.

Transition rate	25-49	50-69	70-79	Reference
**HPV infection to CIN1 progression**	0.49	0.35	0.35	Calibrated
**CIN1 to CIN2 progression**	1.17	1.42	1.42	
**CIN2 to CIN3 progression**	1.79	2.54	3.04	
**CIN3 to cervical cancer progression**	10.86	25.72	30.64	
**HPV infection clearance to immune (females) or susceptible (males)**	0.79	0.51	0.45	
**CIN1 to HPV infection regression**	1.00	1.00	1.00	
**CIN2 to CIN1 regression**	1.00	1.00	1.00	
**CIN3 to CIN2 regression**	0.77	0.27	0.14	

d. Historical interventions

i. HIV antiretroviral therapy (ART)

Beginning in 2005, we model the effects of ART in populations that achieve viral suppression. People living with HIV who initiate ART without achieving viral suppression are assumed to have no benefit from treatment and are not tracked in our model. Populations in the ART/viral suppression compartment have reduced HIV-associated mortality, which also decreases over time as the average CD4 count at ART initiation increases.[41-43,64] Additionally, we assume no onward transmission of HIV with ART.[65,66] And women on ART have the same fertility rates as women without HIV.[7]

We define ART coverage as the percentage of all persons with HIV who are on treatment and virally suppressed. Reflecting historical treatment eligibility policy in Kenya,[67] only people with CD4 count ≤200 cells/µL are put on ART in 2005. The ART initiation threshold is subsequently raised to CD4 <250 cells/mm^3 in 2007, CD4 ≤350 cells/mm^3 in 2011, and to CD4 ≤500 cells/mm^3 in 2014. Finally, anyone with HIV regardless of CD4 cell count is eligible for ART from 2016 on, including those with acute infection. Our model recreates the historical ART coverages reported by the Kenya Ministry of Health.[68-70] However, due to treatment non-adherence and discontinuation as well as the CD4-based eligibility criteria,[71,72] the proportion of viral suppression among all people living with HIV was likely lower than the reported historical ART coverage levels, especially in the earlier years of ART. To adjust for this, we assume that 43% of people on ART are virally suppressed before 2013.[68] We increase the proportion virally suppressed among people on ART linearly from 43% to 75% between 2013-2015,[68] and from 75% to 90% from 2015 to 2018.[70] The proportions of all people with HIV who are virally suppressed in our model is listed in Table S12 and represented in Figure S2. Given the current trajectory, we assume that Kenya will achieve the Joint United Nations Programme (UNAIDS) on HIV/AIDS 90-90-90 goal to have 72.9% of all people living with HIV virally suppressed by 2030. We do not model ART discontinuation (i.e., loss of viral suppression). As a result, the cumulative probability of being on ART increases with age.

Trends in HIV-associated mortality on treatment mirror changes to the ART initiation threshold to reflect higher baseline health among persons initiating treatment over time. HIV-associated mortality with treatment is relative to background mortality.[41-43] From 2004 to 2011, HIV-associated mortality among people with HIV on ART is 0.5 times the background rate. This rate decreases to 0.4, 0.25, and 0.15 times the background mortality rate in 2011, 2015, and 2016, respectively.

Table S12. Proportion of persons living with HIV on ART and virally suppressed Kenya. Guided by viral suppression proportion among respondents of KAIS 2012 and the 2016 Kenya AIDS Progress Report and assuming an increasing proportion of individuals on ART achieve viral suppression over time, we derived the following estimates of viral suppression proportion among people living with HIV in Kenya.. Because data was not available for 2014, we

assume a linear increase in viral suppression from 2013 to 2015. We assume that Kenya will achieve UNAIDS 90-90-90 goal by 2030.

Year	Female	Male	Reference
**2005**	2.9	2.2	
**2006**	6.6	4.9	
**2007**	9.2	6.8	
**2008**	12.5	9.3	
**2009**	23.0	19.6	
**2010**	31.4	26.7	69,70,73
**2011**	35.3	30.0	
**2012**	38.7	32.9	
**2013**	44.4	37.7	
**2015**	44.8	38.0	
**2016**	54.2	46.1	
**2017**	63.0	53.6	
**2030**	72.9	72.9	

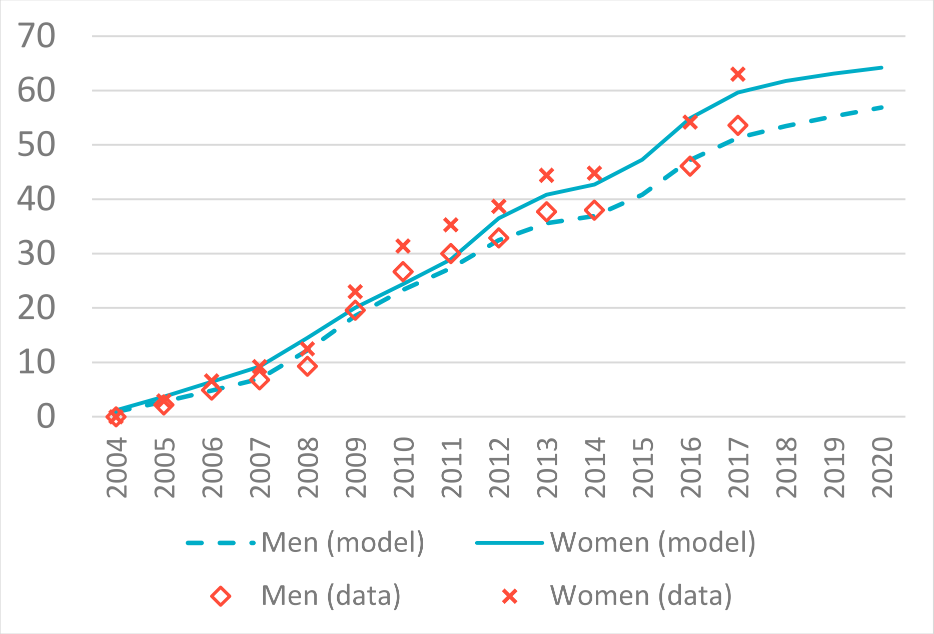

Figure S2. Viral suppression proportion among all men and women with HIV in the model (blue), compared to adjusted observed data (red).

ii. Condoms

Condoms use is initiated in the model in 1995 and stabilized in 2000. Based on data from observational studies and DHS surveys, we assume the condom use varied by sexual risk group, with people in the highest risk group most likely to use condoms.[10,74,75] We specify that condom use was 12% in the low risk group, 25% in the medium risk group, and 35% in the high risk group. We assume condoms reduce HIV acquisition in both males and females by 80%.[76] However, we assume no protection against HPV acquisition with condom use.[77-80]

iii. Circumcision

We assume that circumcised men without HIV have 55% lower risk of HIV acquisition compared to uncircumcised men,[81] however, their risk for HPV acquisition is not reduced.[82-85] We also assume no protective effect of circumcision against HPV infection for men with HIV.[86] In addition, while circumcision does not reduce the risk of HIV transmission to female partners,[87,88] we assume that women with circumcised, HPV-infected male partner have 23% lower risk of HPV acquisition.[89]

We model medical circumcision beginning in 1960 for 15-19 and 20-24 age groups to account for the traditional practice of male circumcision as a rite of passage for many young men in Kenya.[90] For this reason, the national prevalence of circumcision was >80% in 2003[91] before the campaigns to scale up circumcision began in 2008.[92] We adjusted the circumcision rate so that the proportion of men circumcised matches the coverage levels reported in 2003, 2008-2009, and 2014 DHS surveys (Table S13).[10,91,93] The prevalence of circumcision among men without HIV between 2000 and 2020 in the model is illustrated in Figure S3. The model does not track circumcision among men with HIV. We assume circumcision coverage scales up to 90% in all ages by 2030.

Table S13. Proportion of men without HIV who are circumcised.

Year	15-19	20-24	25-29	30-39	40-49	Reference
**2003**	71.5	89	88.3	89.3	83.7	91
**2008**	75.8	88.6	85.1	89.5	91.9	93
**2014**	87.1	96.5	94.6	93.4	91.9	10

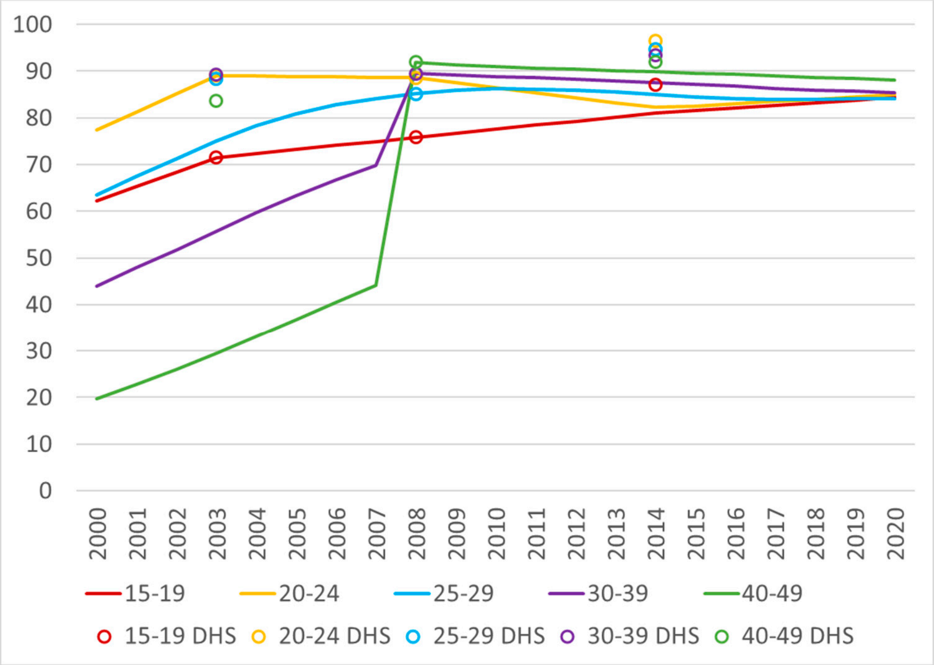

Figure S3. Prevalence of male circumcision among men without HIV in the model (lines) compared to DHS estimates for the entire Kenyan male population (circles).

iv. HPV vaccination

Vaccine Type:
Our model was designed to evaluate the impact of the nonavalent HPV vaccine, in that we group HPV infections into vaccine-targeted types (HPV 16, 18, 31, 33, 45, 52, and 58) and other high-risk HPV types. However, the current vaccination program in Kenya uses the quadrivalent vaccine, which targets only two of the seven oncogenic types in the nonavalent vaccines (types 16 and 18). To account for this, we adjust the vaccine coverage by a factor of (0.7/0.9), based on evidence that HPV types 16 and 18 contribute to approximately 70% of cervical cancer cases relative to the 90% attributable to one or more of the types included in the nonavalent vaccine.[94] In this calculation, we do not account for cross-protection against additional HPV types.

Vaccine Efficacy:
For two-dose vaccine strategy scenarios, we assume lifelong protection against covered types. Two-dose quadrivalent vaccine efficacy is randomly selected from a beta probability distribution[95] based on results from the FUTURE I and II trials, with an efficacy of 100% (95% CI 88.4, 100).[96] The parameters of the beta distribution are α=10.76 and β=0.15, and the distribution is shown in Figure S4.

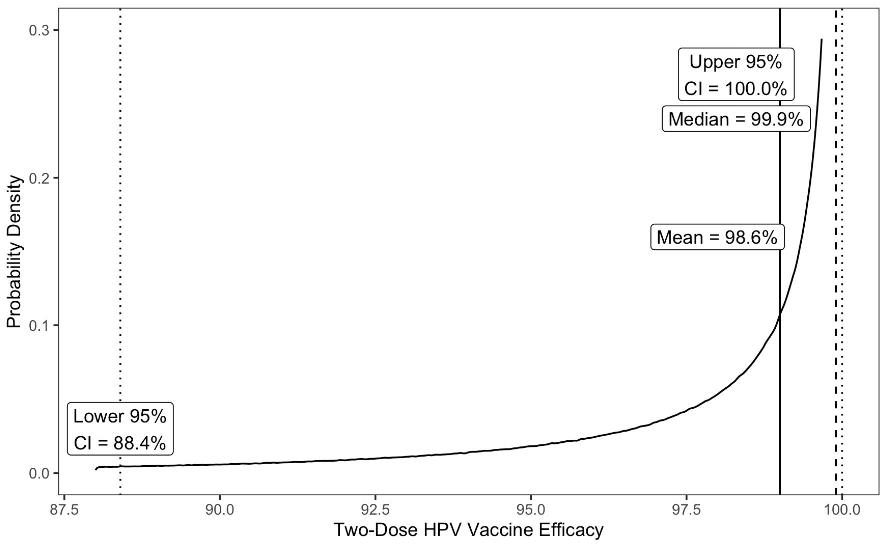

Figure S4. Beta probability density of two-dose HPV vaccine efficacy based on results from the FUTURE I and II trials.

For one-dose vaccine strategy scenarios, we assume lifelong protection against covered types and ran scenarios with various possible waning of vaccine efficacy. One-dose vaccine efficacy is randomly selected from a beta probability distribution[95] based on the most up-to-date results from the KEN SHE trial. The KEN SHE study evaluated the efficacy of the bivalent and nonavalent vaccines. Although this model assumes a national Kenyan strategy using the quadrivalent vaccine, we modeled vaccine efficacy based on the KEN SHE bivalent vaccine results since both the bivalent and quadrivalent vaccines protect against oncogenic HPV types 16 and 18. The KEN-SHE study shows a bivalent vaccine median efficacy of 97.5% (95% CI 90.0, 99.4).[97] A beta distribution was estimated to model the median KEN SHE bivalent vaccine efficacy results, as shown in Figure S5, and has parameters α=49.7 and β=1.6.

For the purposes of this study, we assumed that one-dose efficacy is equivalent or inferior to two doses. As a result, for each of the 25 parameter sets, we bounded one-dose efficacy so that it was equal to or less than two-dose efficacy. As a sensitivity analysis, we also ran a scenario with unbounded one-dose efficacy to assess the impact of this assumption. The results of this analysis can be found in Section IVa of this Supplementary Materials.

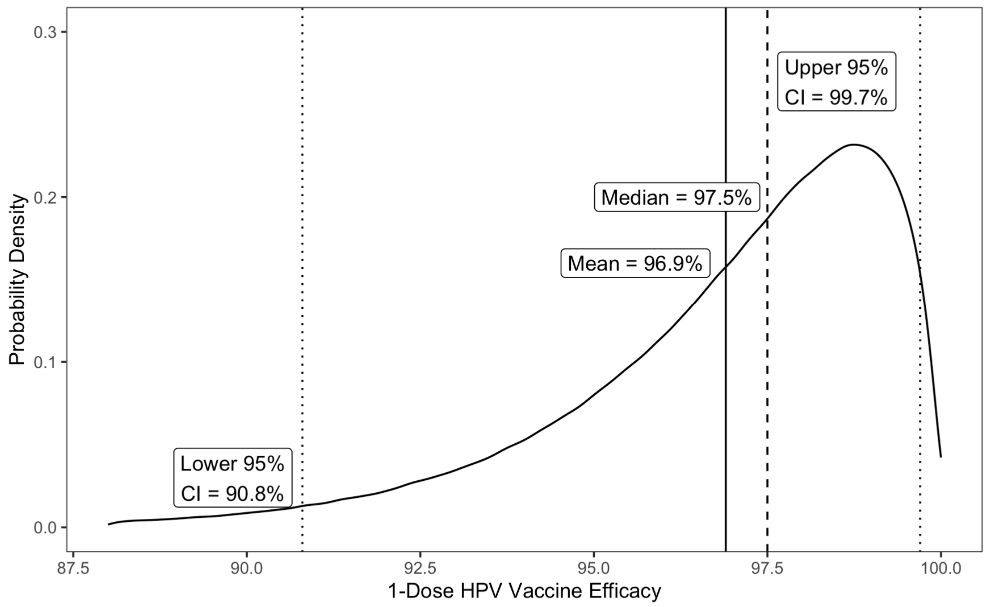

Figure S5. Beta probability density of one-dose HPV vaccine efficacy based on results from the KEN SHE study.

Vaccine Coverage:
From 2019-2020, we assume a linear scale-up of two-dose quadrivalent vaccination coverage of girls by the age of 10 years from 0% to 16%.[98] Then from 2020-2023, we assume a linear scale up from 16% to 31% of two-doses.[99] Starting in 2023, vaccination coverage and number of doses varies depending on the scenario being evaluated.

v. HPV screening and treatment

Beginning in 2000, we model once-per-lifetime cervical cancer screening for women in the age range of 35-39. Although the Kenyan Ministry of Health during this time[100] recommended screening every 5 years beginning at age 30, observed data suggest low compliance with this schedule.[101] Informed by consultation with in-country experts, we conservatively assume only one lifetime screen in the model. Based on data from literature of the proportion of women who report ever receiving cervical cancer screening, we assume 14% screening coverage in women without HIV[102] and 56% screening coverage among women with HIV[103]. We assume that women are screened using visual inspection with acetic acid (VIA) with a sensitivity of 62%.[104] Of the women who test positive, 72% return for triage using colposcopic biopsy.[105] Of those confirmed to have CIN2-3, we assume 50% are lost to follow up[105] while the remaining 50% are treated with cryotherapy, which has an efficacy of 97% in women without HIV and 66% in women with HIV.[106,107] These rates reflect challenges with follow-up and retention for cervical cancer prevention and treatment, particularly with a multi-visit screening and treatment approach.[108-111]

Additionally, evidence suggests that 28% of women treated for CIN2-3 have persistent HPV infection, including cases with residual or recurrent CIN.[112] Because this estimate is drawn from studies of primarily women without HIV, we used the estimate of 9% treatment failure in women without HIV above to calculate the percent of women who would have persistent HPV with successfully treated lesions (18.5%). Lacking data on differential HPV persistence by HIV status, we assume that HPV persistence after lesion clearance is the same among women living with HIV. Women with a successfully treated lesion who also clear HPV are assumed to develop temporary, partial immunity against reinfection with the same modeled HPV type (vaccine-targeted type vs. other hrHPV).

Women with CIN3 who transition to cervical cancer undergo cancer progression as illustrated in Figure S6. Undiagnosed cancers can be detected either by symptoms or by screening. Among women confirmed to have cervical cancer, 40% return for treatment.[108-111] Out of the women who receive cancer treatment, they can be treated either by hysterectomy or other modalities such as chemotherapy or radiation. We assume 69.6% of those with local cancer, 4.2% of those with regional cancer, and 0% of those with distant cancer are treated with hysterectomy. This was determined based on (1) previous modeling studies that assumed 100% hysterectomy treatment for stage IA1 to stage IB1[63], (2) consultation with in-country clinicians who estimate 30% hysterectomy treatment for stage IB2 and IIA and 0% hysterectomy treatment for stage IIA to IV, (3) defining local cancers in the model as stage I, regional as stages II and III, and distant as stage IV, (4) data in the literature showing that 87% of local cancers in Kenya are diagnosed at stage IB and 14% of regional cancers in Kenya are diagnosed at stage IIA[113], and (5) lacking data from literature showing the fraction of cervical cancers in Kenya diagnosed at stage IB1 versus IB2, assuming that half of stage IB cancers are diagnosed at stage IB1 and the other half at IB2.

All women with cervical cancer can die from cervical cancer or other causes. Cervical cancer-associated mortality rates are greater for later stages and decreasing CD4 counts, and mortality rates are lower for treated cancers compared to untreated cancers, as shown in Table S10. Women who are treated by hysterectomy do not experience cervical cancer-associated mortality because we assume that hysterectomy is 100% effective at treating cervical cancer. After hysterectomy, women are assumed to be infertile, unable to acquire or transmit HPV, and to have no increase in mortality due to their prior cancer status. We assume cervical screening and treatment are equally effective for both vaccine-targeted type and other hrHPV. For women infected with both, their disease status is determined by her most advanced HPV type. Finally, we do not account for recurrence of late-stage cancers that have spread to other organ systems.

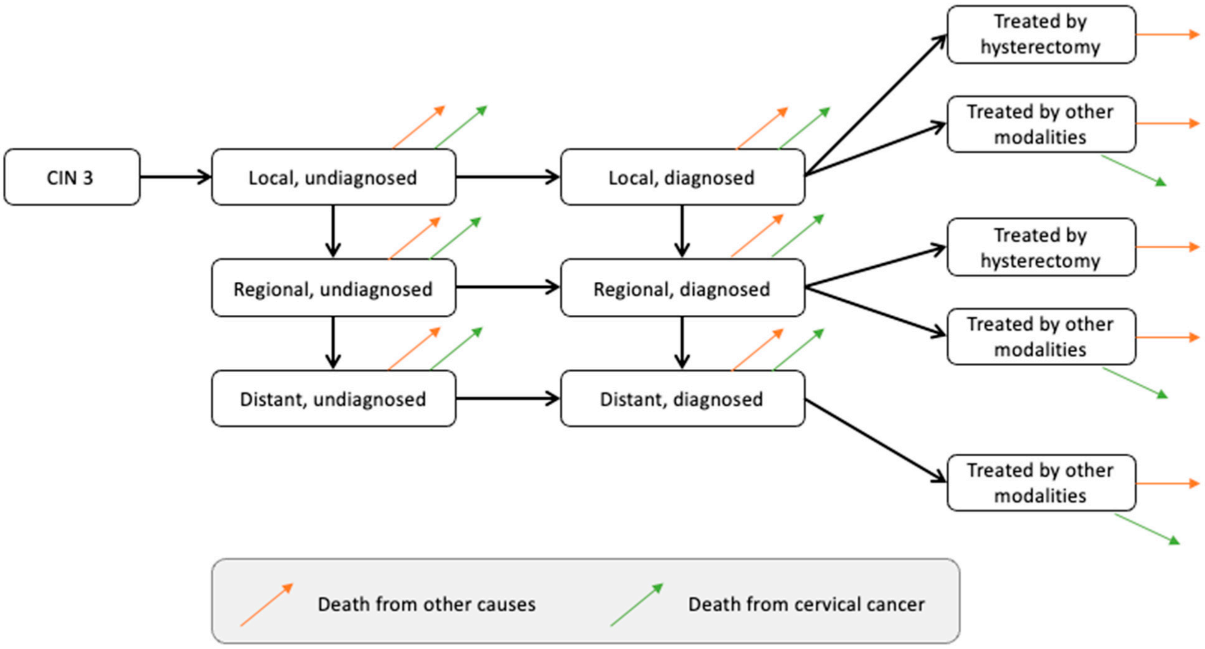

Figure S6. Model schematic illustrating cervical cancer progression from undiagnosed, diagnosed, to treated. Death can be due to cervical cancer or other causes. The only exception to this is women treated with hysterectomy, who only experience death from other causes since we assume hysterectomy is 100% effective at treating cervical cancer. Only untreated cancers progress to later stages.

III. Calibration and validation

a. Calibration

The initial model parameter values were based on a previously published version of the model fitted to KwaZulu-Natal, South Africa; the calibration method is discussed in detail in the Supplemental Materials to the publication.[50] For this current modeling study, we manually calibrated the parameters to fit to HIV, HPV, and cervical cancer epidemiology in 2000-2020 in Kenya. Our calibration targets are listed in Table S14. We calibrated the parameters related to demographics to match UNPD-estimated population size from 1950-2070. We based sexual behavior parameters on data reported in the 2014 Kenya DHS,[10] then calibrated these parameters to produce time and age trends in HIV and HPV prevalence that match the observed data. To calibrate sexual behaviors, we assume that people in higher risk groups have more sex partners per year but have fewer coital acts per partnership, reflecting higher partnership formation rate and shorter partnership duration. We calibrate the age-specific number of coital acts per partnership in women. We assume men aged 10-19 have the same number of acts per partnership as women, whereas men aged 20-79 have equal acts to women of the next lowest age group, reflecting age disparities in partnerships.[12]

To reflect higher transmission probability of non-vaccine-targeted types, we calibrate the HPV transmission probability for vaccine-targeted type, then we apply a multiplier on this probability to approximate the transmission probability of non-vaccine-targeted types. We adjust this multiplier so that relative distribution of types matches those observed in studies from sub-Saharan Africa (Table S15). We calibrated HPV and CIN progression parameters to cervical cancer incidence estimates from GLOBOCAN 2012[114] for Kenya due to a lack of high-quality national-level data. Although there two regional cancer registries in Kenya, their coverage is limited to the urban populations in Nairobi and Eldoret.[115] As a result, the registries may be underestimating the cancer burden in the country. Moreover, low coverage of cervical cancer screening uptake and incomplete case-finding within the registries' catchment areas could further contribute to the underestimation of cervical cancer burden. The value for the relative risk of HIV acquisition with HPV infection is randomly selected from a uniform distribution with a minimum of 1.6 and maximum of 2.2.[21] We ran 100 iterations, and report the median and the interquartile range of outcomes.

The main difference between this version of the model and the previously published version by Liu et al is the addition of symptomatic cervical cancer detection. Three parameters were calibrated as part of this addition to the model: the

probability of symptomatic detection in local, regional, and distant stages. These parameters were calibrated by using the prior distribution from by Myers et al and Van Schalwyk et al, assuming a uniform distribution.[61,62] Prior distributions for the probability of symptom diagnosis for local cancers were 0-0.03, regional cancers were 0-0.8, and distant cancers were 0.7-1.0.[61,62] During the calibration of these parameters, all other parameters sets were fixed and the relative risk of HIV acquisition with HPV infection was randomly selected from a uniform distribution. The model was calibrated to fit observed data on cervical cancer stage distribution in Kenya between 2017-2020.[113]

Table S14: Calibration targets.

	Year	Age	Value	Reference
**Total population size**	2000	0-79	31890777	3
	2005	0-79	36547290	
	2010	0-79	41942714	
	2015	0-79	47765057	
	2020	0-79	53627022	
	2025	0-79	59981315	
	2030	0-79	66449655	
	2035	0-79	73026286	
	2040	0-79	79469672	
	2045	0-79	85669262	
	2050	0-79	91575092	
	2055	0-79	97174763	
	2060	0-79	102398265	
	2065	0-79	107170157	
	2070	0-79	111411341	
**Population age distribution, men and women combined**	2010	0-9	0.31	3
		10-19	0.23	
		20-29	0.19	
		30-39	0.12	
		40-49	0.07	
		50-59	0.04	
		60-79	0.03	
**Population age distribution, men and women combined**	2020	0-9	0.31	3
		10-19	0.23	
		20-29	0.19	
		30-39	0.12	
		40-49	0.07	
		50-59	0.04	
		60-79	0.03	
**HIV Prevalence, men**	2003	15-19	0.004	91
		20-24	0.024	
		25-29	0.073	
		30-34	0.066	
		35-39	0.084	
		40-44	0.088	
		45-49	0.052	
		15-49	0.046	
	2007	15-19	0.01	116
		20-24	0.02	
		25-29	0.07	
		30-34	0.09	
		35-39	0.09	
		40-44	0.10	
		45-49	0.06	
		15-49	0.056	
	2009	15-19	0.007	93
		20-24	0.015	
		25-29	0.065	
		30-34	0.068	
		35-39	0.104	
		40-44	0.057	
		45-49	0.043	
		15-49	0.046	
	2012	15-19	0.009	117
		20-24	0.013	
		25-29	0.043	

		30-34	0.066	
		35-39	0.05	
		40-44	0.081	
		45-49	0.089	
		15-49	0.044	
**HIV prevalence, women**	2003	15-19	0.030	91
		20-24	0.090	
		25-29	0.129	
		30-34	0.117	
		35-39	0.118	
		40-44	0.095	
		45-49	0.039	
		15-49	0.087	
	2007	15-19	0.035	116
		20-24	0.074	
		25-29	0.102	
		30-34	0.133	
		35-39	0.112	
		40-44	0.094	
		45-49	0.088	
		15-49	0.09	
	2009	15-19	0.03	93
		20-24	0.06	
		25-29	0.10	
		30-34	0.11	
		35-39	0.09	
		40-44	0.14	
		45-49	0.06	
		15-49	0.08	
	2012	15-19	0.011	117
		20-24	0.046	
		25-29	0.079	
		30-34	0.066	
		35-39	0.123	
		40-44	0.106	
		45-49	0.107	
		15-49	0.069	
**HPV prevalence, women without HIV**	2005	20-24	0.29 (0.25-0.52)	118,119
		25-29	0.30 (0.12-0.47)	
		30-39	0.26 (0.13-0.39)	
		40-49	0.24 (0.10-0.39)	
**HPV prevalence, women with HIV**	2005	20-24	0.66 (0.58-0.75)	
		25-29	0.67 (0.56-0.79)	
		30-39	0.57 (0.45-0.71)	
		40-49	0.56 (0.43-0.68)	
**Cervical cancer incidence, per 100,00 women**	2012	15-19	0.7	114
		20-24	0.8	
		25-29	8.0	
		30-34	21.0	
		35-39	40.7	
		40-44	62.7	
		45-49	83.1	
		50-54	103.4	
		55-59	130.3	
		60-64	150.5	
		65-69	156.3	
		70-74	150.7	
		75-79	133.2	
		15-79	40.0	
**Proportion of cervical cancers diagnosed at each stage**	2017-2020	Local	0.213	113
		Regional	0.667	
		Distant	0.120	

Table S15. Relative distribution of HPV types (vaccine-targeted vs non-vaccine-targeted) at each stage of HPV infection and progression.

Criteria	HPV Type	Value	Reference
**HPV**	Vaccine-targeted	0.4682	120,121
	Non-vaccine-targeted	0.5318	
**CIN1**	Vaccine-targeted	0.5192	120,122
	Non-vaccine-targeted	0.4808	
**CIN3**	Vaccine-targeted	0.7371	47,120,123
	Non-vaccine-targeted	0.2629	
**Cervical cancer**	Vaccine-targeted	0.8578	46,94,120,124
	Non-vaccine-targeted	0.1422	

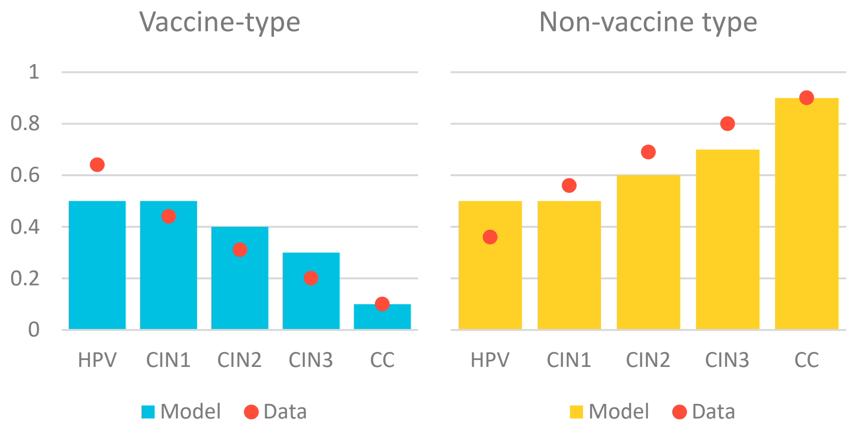

Figure S7. Distribution of HPV types at each HPV state in the model compared to data.

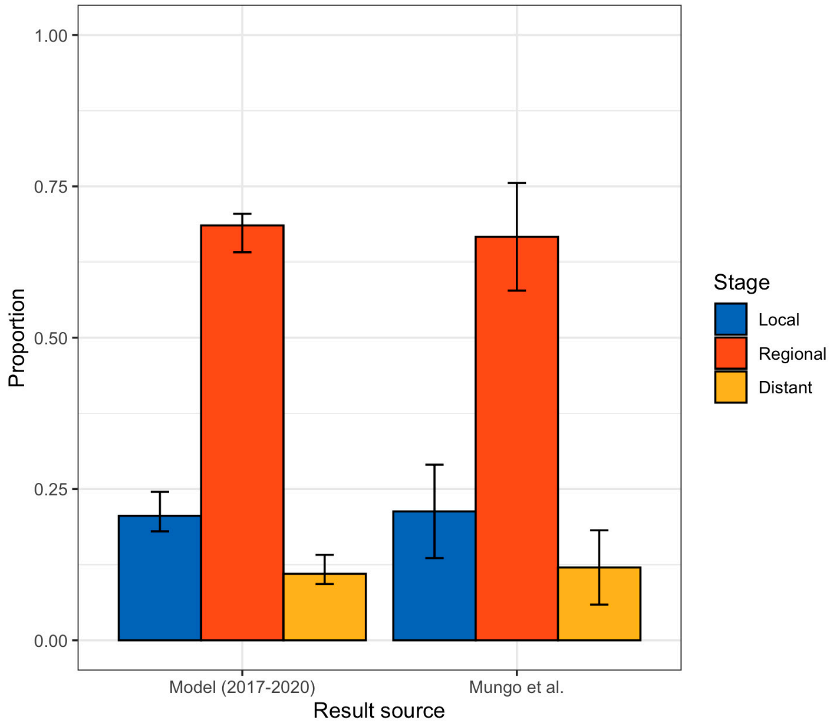

Figure S8. Distribution of diagnosed cervical cancer stages in the model compared to data from 2017-2020 from Mungo et al.[113]

b. Validation

We validated our HIV natural history module to additional time points and data sources (Table S16). We compared our model HIV output to age- and sex-specific HIV prevalence reported in the Population-based HIV Impact Assessment (PHIA) in 2018, which used a similar sampling method as DHS and KAIS surveys.[70] To validate cervical cancer incidence projection, we compared model output against GLOBOCAN 2020.[125]

Table S16. Validation targets.

	Year	Age	Value	Reference
**HIV Prevalence, men**	2018	15-19	0.005	
		20-24	0.006	
		25-29	0.022	
		30-34	0.032	70
		35-39	0.043	
		40-44	0.063	
		45-49	0.083	
		15-49	0.031	
**HIV Prevalence, women**	2018	15-19	0.012	
		20-24	0.034	
		25-29	0.060	
		30-34	0.095	70
		35-39	0.087	
		40-44	0.119	
		45-49	0.106	
		15-49	0.062	
**Cervical cancer incidence, per 100,00 women**	2020	15-19	0.2	
		20-24	0.38	
		25-29	3.5	
		30-34	14.4	
		35-39	29.1	
		40-44	50.4	
		45-49	72.9	125
		50-54	91.4	
		55-59	109.7	
		60-64	122.3	
		65-69	124.7	
		70-74	114.1	
		75-79	80.67	
		15-79	45.7	

c. Comparing model output to data

Demographics

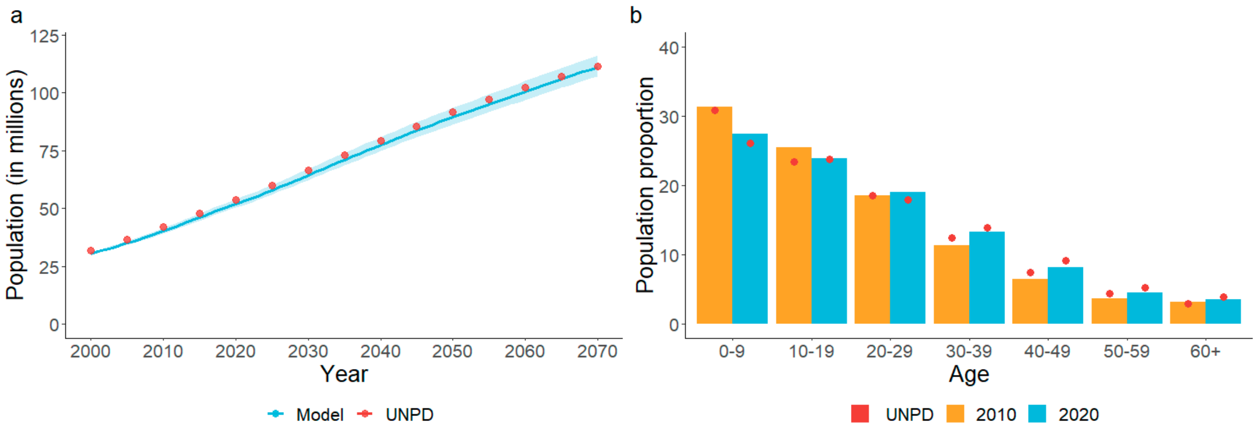

Figure S9. a). Model-projected total population size, including men and women, over time (blue line) compared to UNPD estimates for Kenya (red dots). b). Age distribution of model population, in 10-year age groups, in 2010 (yellow) and 2020 (blue) compared to UNPD estimates (red).

HIV

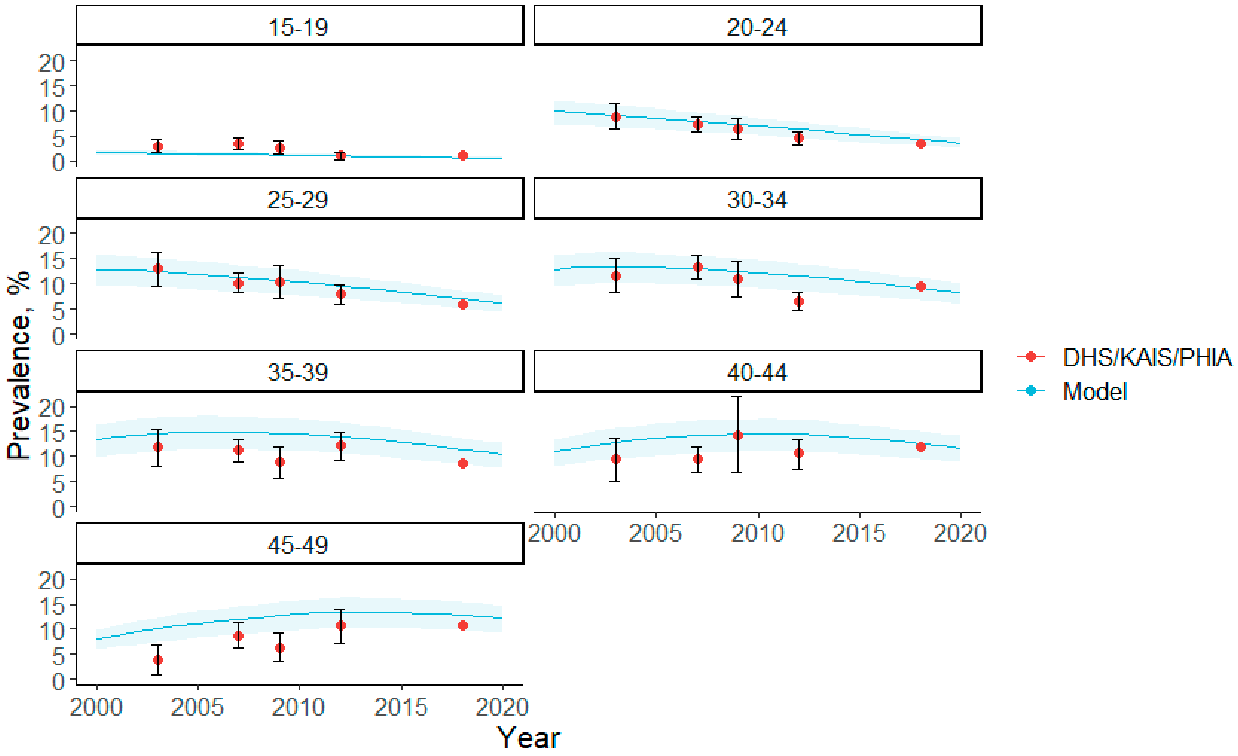

Figure S10. Model-estimated HIV prevalence among women by 5-year age groups (blue) compared to age-specific 2003 DHS, 2007 KAIS, 2008-2009 DHS, 2012 KAIS, and 2018 PHIA data for the same age groups (red). We calibrated to 2003, 2007, 2008-2009, and 2012 values and validated against the 2018 values.

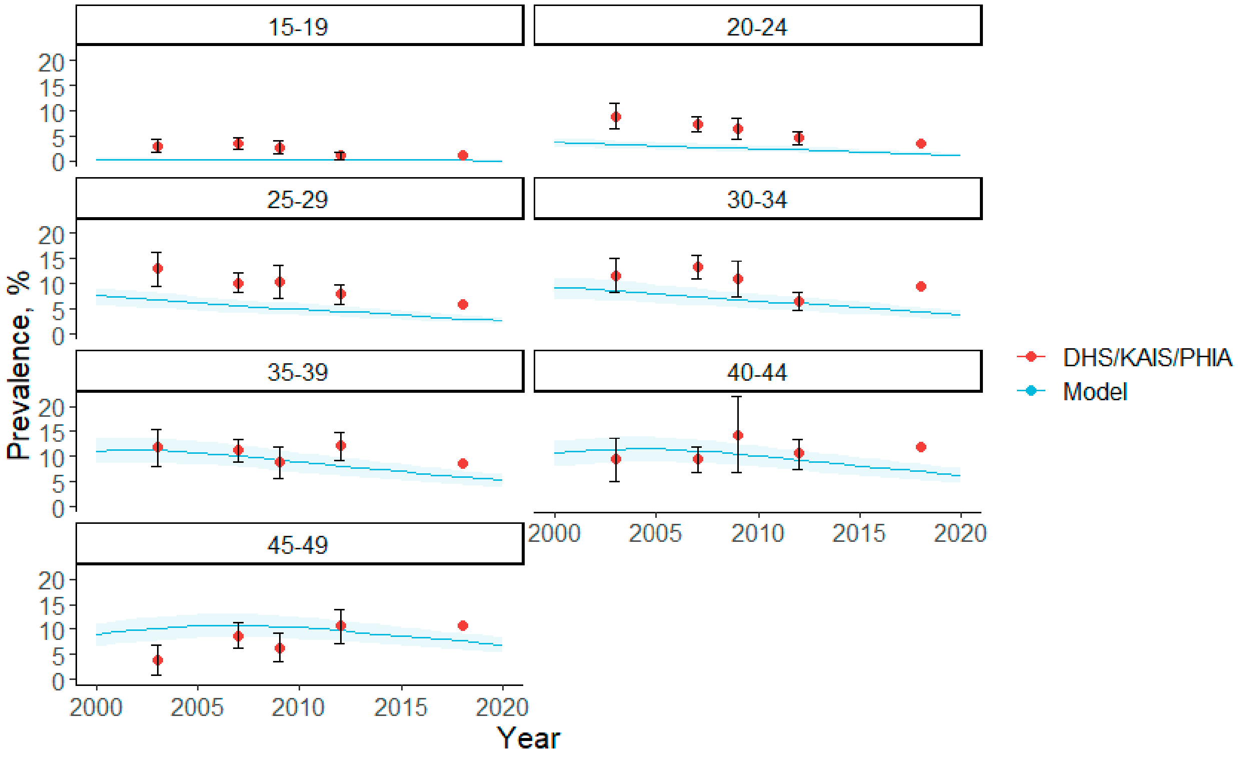

Figure S11. Model-estimated HIV prevalence among men by 5-year age groups (blue) compared to age-specific 2003 DHS, 2007 KAIS, 2008-2009 DHS, 2012 KAIS, and 2018 PHIA data for the same age groups (red). We calibrated to 2003, 2007, 2008-2009, and 2012 values and validated against the 2018 values.

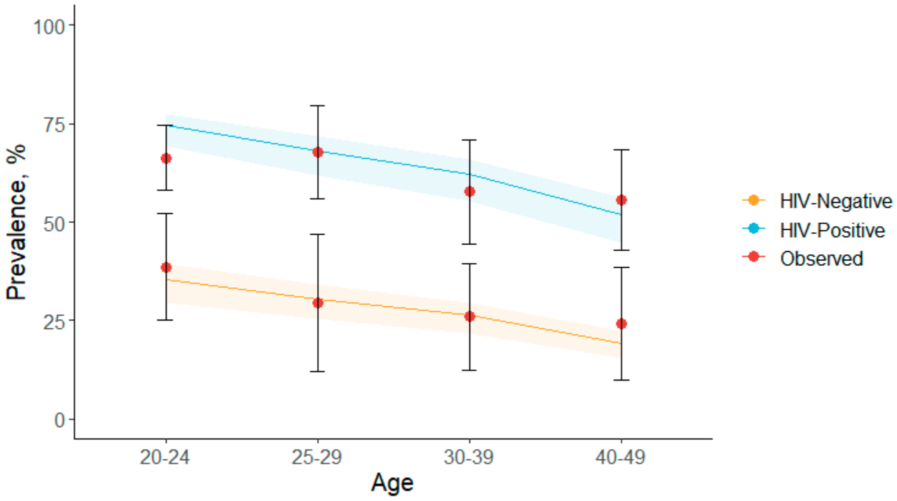

Figure S12. Model estimated HPV prevalence by 10-year age group among women without HIV (yellow) and women with HIV (blue) compared to estimates from observational studies.[118,119]

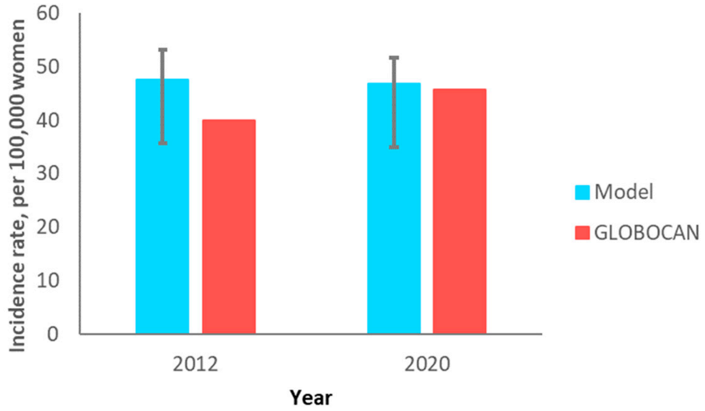

Figure S13. Age-standardized cervical cancer incidence rates in 2012 and 2020 for women aged 15-79 (blue) compared to age-standardized estimates for women of the same age from GLOBOCAN (red).

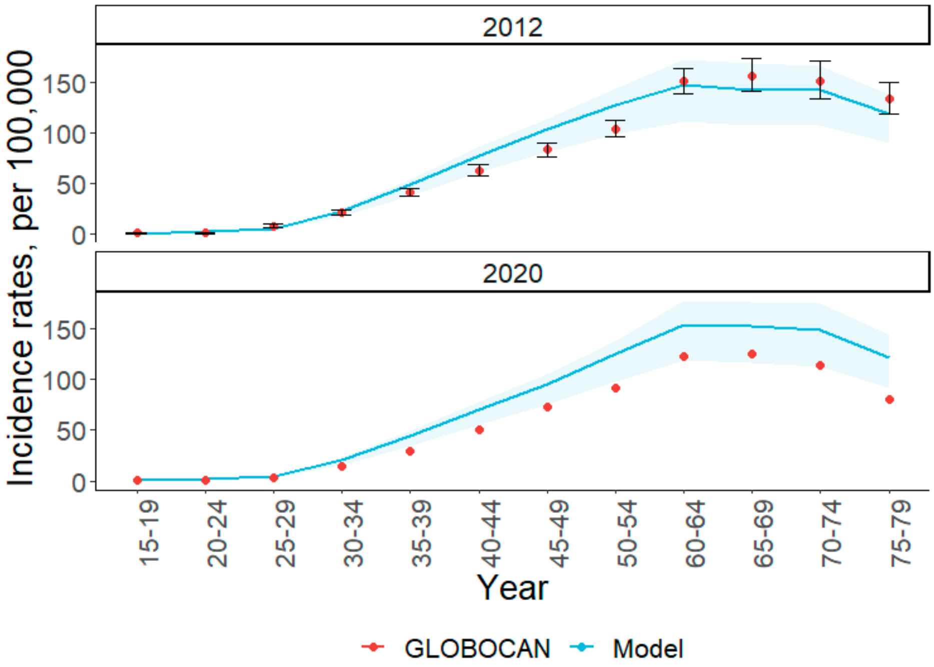

Figure S14. Model-estimated age-specific cervical cancer incidence rates (blue), compared to GLOBOCAN estimates (red). We calibrated to 2012 GLOBOCAN data and validated against 2020 GLOBOCAN data.

IV. Additional results

a. Sensitivity Analysis: Changing the Screening Technology

In all the scenarios explored in this study, we kept screening constant throughout the time horizon to isolate the impact of HPV vaccination on cervical cancer outcomes, and assuming screening by VIA, triage by colposcopy, and treatment by cryotherapy. This is in line with the screening modality predominantly used in Kenya today.[104] However, the country is expanding capacity for screening by HPV DNA and treatment by thermal ablation.[99] As a sensitivity analysis, we re-ran the one-dose 90% coverage lifelong efficacy scenario but changed the screening modality to HPV DNA, and then another scenario with HPV DNA screening and thermal ablation treatment. The aim of this sensitivity analysis was to assess the impact of the screening and treatment technology on the outcomes of the study.

Assumptions were made for screening test performance, retention to treatment, and treatment failure, which has been described extensively in previous modelling studies.[126] We assumed HPV DNA sensitivity of 85% for HIV-negative women, 94% for untreated women with HIV, and 90% for virally suppressed women with HIV.[127] We also assumed a 5% loss to follow-up for thermal ablation. Finally, we assumed that 39% of women treated for CIN2-3 with thermal ablation have persistent HPV infection.[112]

As shown in Figure S15A, the screening or treatment modality does not significantly change the age-adjusted cervical cancer incidence rate throughout the time horizon. Figure S15B shows similar results for cervical cancer cases averted. One explanation for this is that each scenario was run with current once in a lifetime screening coverage rates of 14% coverage for women without HIV[102] and 56% screening coverage among women with HIV[103]. We expect screening coverage to continue to increase, especially for women living without HIV, as Kenya plans to ramp up screening coverage to meet the WHO cervical cancer elimination targets of 70% of women screened by ages 35 and 45.[99] Because the purpose of this study was to isolate the impact of vaccination alone, we did not adjust screening coverage rates. However, we plan to supplement this work with future studies varying both vaccination and screening coverage and technologies.

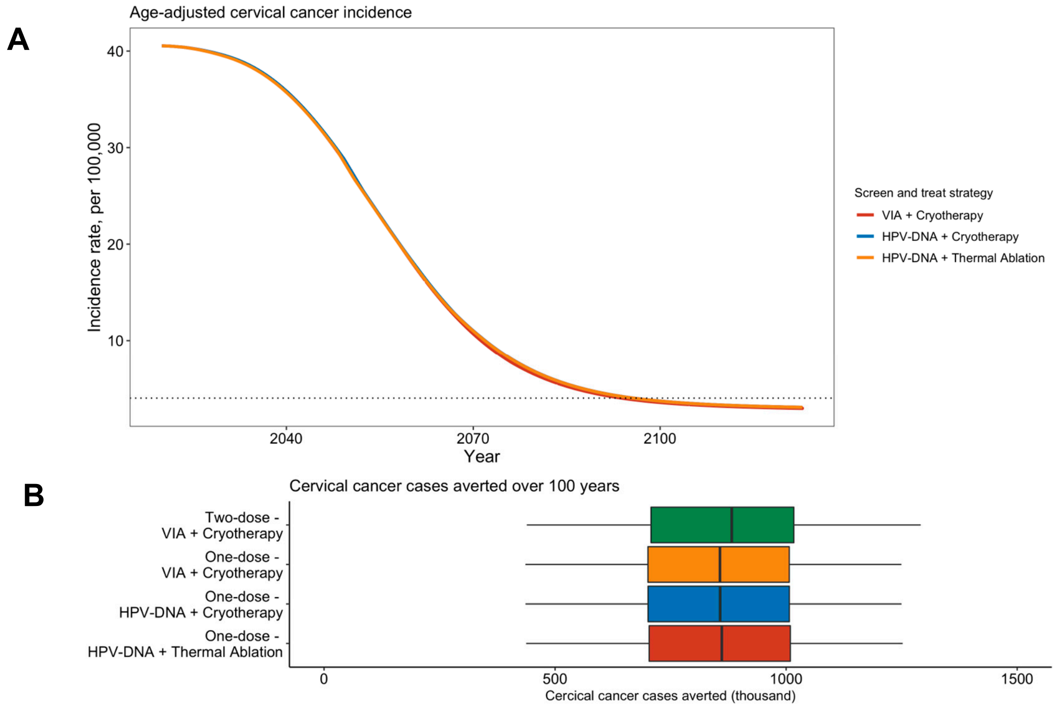

Figure S15. Sensitivity analysis showing the impact of running the 90% one-dose lifelong efficacy scenario with VIA and cryotherapy, HPV DNA and cryotherapy, and HPV DNA and thermal ablation on (A) age-adjusted cervical cancer, and (B) cervical cancer cases averted over a 100-year time horizon.

b. Sensitivity Analysis: Unbounding Single Dose Efficacy

An additional sensitivity analysis was explored to assess the impact of unbounding single dose HPV vaccination efficacy on cervical cancer outcomes. In the main analysis, we ran 25 parameter sets for each scenario in which HPV vaccine efficacy is pulled from a beta probability distribution. Two-dose vaccine efficacy is pulled from a beta distribution that reflects the results of the FUTURE I and II trials, and one-dose vaccine efficacy is pulled form a beta distribution that reflects the results of the KEN SHE trial. A key assumption that was conservatively made for each parameter set is that two-dose efficacy is always at least as high as single-dose efficacy. We ran a sensitivity analysis to assess the impact of this assumption on the modeling results. We re-ran the one-dose 90% coverage lifelong efficacy scenario without imposing the bounds on one-dose vaccine efficacy.

Figure S16 shows that by unbounding one-dose HPV vaccine efficacy, the number of cervical cancer cases averted is greater than originally predicted, and the results are more aligned to the two-dose 90% coverage results. This suggests that the main analysis of this study is likely underestimating the impact of single-dose HPV vaccination on long-term cervical cancer outcomes in Kenya.

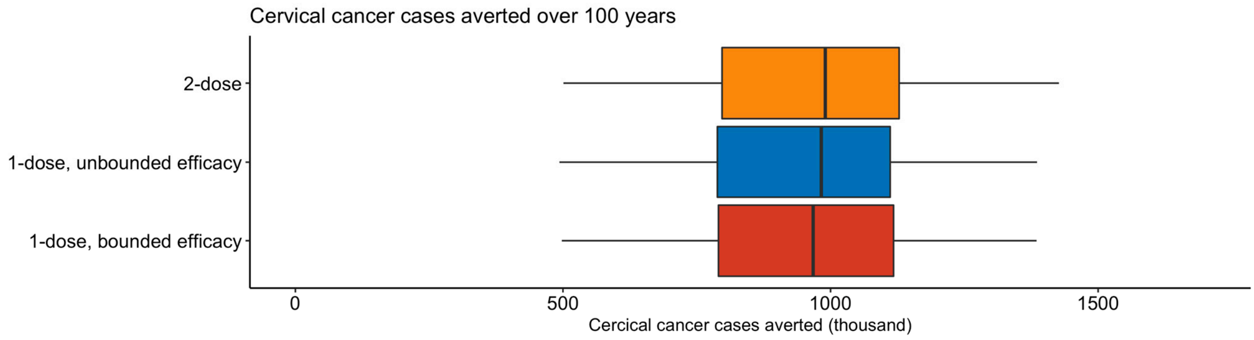

Figure S16. The impact of unbounding one-dose HPV vaccine efficacy (blue) compared with the two-dose 90% coverage results (orange). The box and whisker plot in red is the original one-dose 90% coverage results, assuming one-dose vaccine efficacy is always lower than or equal to two-dose efficacy for each parameter set.

c. Additional Modelling Results

In addition to evaluating the impact of vaccination strategies on cervical cancer incidence, cases averted, and DALYs averted, we also looked at the impact on cervical cancer mortality, shown in Table S17 below.

Table S17. Projected cervical cancer mortality rates from the years 2023 to 2123. Results are presented as a median and 90% confidence interval using 25 parameter sets.

Dose and durability	Cover	Additional strategy	Mortality rate, per 100,000	Percent reduction in mortality
One-dose, lifelong efficacy scenarios				
No vaccination	0%		3.8 (1.8-5.7)	Reference
Two-dose	31%		2.2 (0.9-3.6)	25.6 (23.3-29.3)
	50%		1.4 (0.5-2.4)	39.4 (36.4-43.4)
	70%		0.8 (0.3-1.5)	50.4 (46.5-53.3)
	77%		0.7 (0.3-1.2)	53.2 (49.1-55.8)
	90%		0.5 (0.3-0.9)	57.5 (52.7-58.8)
One-dose, lifelong efficacy	77%		0.9 (0.3-1.6)	49.2 (44-51.9)
	70%		0.7 (0.3-1.3)	51.9 (47.1-54.2)
	90%		0.6 (0.3-0.9)	56.3 (51.3-58)
One-dose, waning efficacy scenarios				
One-dose, 20EP/20WP	90%		0.6 (0.3-1.3)	52.5 (47.2-55)
One-dose, 25EP/20WP	90%		0.6 (0.3-1.1)	54.4 (49.7-56.5)
One-dose, 30EP/20WP	90%		0.6 (0.3-1)	55.5 (50.7-57.4)
One-dose, 20EP/10WP	90%		0.8 (0.3-1.6)	50 (42.8-53.4)
One-dose, 25EP/10WP	90%		0.6 (0.3-1.2)	53.5 (48.2-55.6)
One-dose, 30EP/10WP	90%		0.6 (0.3-1)	55 (50.3-57.1)
One-dose, using cost savings to invest in an additional vaccination strategy, lifelong and waning efficacy scenarios				
One-dose, lifelong efficacy	90%	CU females age 11-19	0.5 (0.3-0.9)	59.8 (54.8-61.5)
	90%	CU females age 11-24	0.5 (0.3-0.9)	59.9 (55-61.7)
	90%	Vaccination for all by age 10	0.5 (0.3-0.7)	61.2 (56-62.2)
One-dose, 30EP/20WP	90%	CU females age 11-19	0.6 (0.3-1)	59.4 (54.5-61.3)
	90%	CU females age 11-24	0.6 (0.3-1)	59.4 (54.7-61.4)
	90%	Vaccination for all by age 10	0.5 (0.3-0.7)	60.9 (55.6-61.8)
One-dose, 20EP/10WP	90%	CU females age 11-19	0.8 (0.3-1.6)	55.1 (47.4-58.4)
	90%	CU females age 11-24	0.8 (0.3-1.6)	55.2 (47.5-58.6)
	90%	Vaccination for all by age 10	0.5 (0.3-1)	56.1 (51.3-58.2)

d. Additional Economic Results

i. Pairwise Cost Effectiveness Analysis Results and Calculation of Five-Year Cost Savings

We evaluated the economic outcomes of all possible switches from two- to one-dose HPV vaccination schedule, as shown in Table S18 below. We used the mean to calculate the most conservative estimate for potential five-year cost savings if Kenya switches to a single dose strategy. We decided to evaluate cost savings over five years after consultation with experts from the National Vaccines and Immunisations Program within the Ministry of Health. Additionally, Table S19 below presents results from a similar analysis over a 100-year time horizon where we summarized the median of health and economic outcomes of all possible switches from a two- to a one-dose HPV vaccination schedule.

Table S18. DALYs averted over a 5-year time horizon, incremental costs over five-years, and ICERS if Kenya switches from various two-dose scenarios (columns) to one-dose scenarios (rows), with different waning assumptions. Orange signifies that the switch result into reduced cost and reduced DALYs averted, blue signifies that the switch would result into increased costs and increased DALYs averted, and green signifies that the switch would result into increased DALYs averted at lower costs.

		**No Vaccination**	**2-dose, 31%coverage**	**2-dose, 50%coverage**	**2-dose, 70%coverage**	**2-dose, 77%coverage**	**2-dose, 90%coverage**
		Mean	Mean	Mean	Mean	Mean	Mean
**No Vaccination**	DALYs Averted	0	-814,791	-1,291,100	-1,683,990	-1,791,966	-1,951,176
	Cost Increment	0	-$20,100,000	-$41,800,000	-$64,700,000	-$72,700,000	-$87,500,000
	ICER		29	39	47	50	56
**2-dose, 31%coverage**	DALYs Averted	814,791	0	-476,309	-869,199	-977,175	-1,136,385
	Cost Increment	$20,100,000	$0	-$21,700,000	-$44,600,000	-$52,600,000	-$67,500,000
	ICER	29		58	68	72	81
**2-dose, 50%coverage**	DALYs Averted	1,291,100	476,309	0	-392,890	-500,866	-660,076
	Cost Increment	$41,800,000	$21,700,000	$0	-$22,900,000	-$30,900,000	-$45,700,000
	ICER	39	58		82	88	100
**2-dose, 70%coverage**	DALYs Averted	1,683,990	869,199	392,890	0	-107,976	-267,186
	Cost Increment	$64,700,000	$44,600,000	$22,900,000	$0	-$8,003,008	-$22,900,000
	ICER	47	68	82		110	128
**2-dose, 77%coverage**	DALYs Averted	1,791,966	977,175	500,866	107,976	0	-159,210
	Cost Increment	$72,700,000	$52,600,000	$30,900,000	$8,003,008	$0	-$14,900,000
	ICER	50	72	88	110		139
**2-dose, 90%coverage**	DALYs Averted	1,951,176	1,136,385	660,076	267,186	159,210	0
	Cost Increment	$87,500,000	$67,500,000	$45,700,000	$22,900,000	$14,900,000	$0
	ICER	56	81	100	128	139	
**1-dose, 77%coverage**	DALYs Averted	1,731,319	916,528	440,218	47,328	-60,648	-219,857
	Cost Increment	$35,800,000	$15,800,000	-$5,955,062	-$28,800,000	-$36,800,000	-$51,700,000
	ICER	25	23	-19	-407	2,441	390
	DALYs Averted	1,620,310	805,519	329,210	-63,680	-171,656	-330,866

**1-dose, 70%coverage**	Cost Increment	$31,800,000	$11,800,000	-$9,956,564	-$32,800,000	-$40,800,000	-$55,700,000
	ICER	24	19	-41	2,042.93	413	268
**1-dose, 90%coverage**	DALYs Averted	1,899,385	1,084,594	608,284	215,394	107,418	-51,791
	Cost Increment	$43,300,000	$23,200,000	$1,476,299	- $21,400,000	-$29,400,000	-$44,300,000
	ICER	28	29	3	-141	-394	3359
**1-dose, 90%coverage (20EP/20WP)**	DALYs Averted	1,765,248	950,457	474,148	81,258	-26,718	-185,928
	Cost Increment	$43,300,000	$23,200,000	$1,476,301	-$21,400,000	-$29,400,000	-$44,300,000
	ICER	30	32	4	-311	-782	456
**1-dose, 90%coverage (25EP/20WP)**	DALYs Averted	1,841,715	1,026,924	550,615	157,725	49,749	-109,460
	Cost Increment	$43,300,000	$23,200,000	$1,476,299	-$21,400,000	-$29,400,000	-$44,300,000
	ICER	29	30	4	-185	-741	863
**1-dose, 90%coverage (30EP/20WP)**	DALYs Averted	1,880,297	1,065,506	589,196	196,306	88,330	-70,879
	Cost Increment	$43,300,000	$23,200,000	$1,476,299	-$21,400,000	-$29,400,000	-$44,300,000
	ICER	29	29	4	-153	-490	1646
**1-dose, 90%coverage (20EP/10WP)**	DALYs Averted	1,667,964	853,174	376,864	-16,026	-124,002	-283,211
	Cost Increment	$43,300,000	$23,200,000	$1,476,304	-$21,400,000	-$29,400,000	-$44,300,000
	ICER	31	35	5	1043	988	289
**1-dose, 90%coverage (25EP/10WP)**	DALYs Averted	1,800,967	986,176	509,867	116,977	9,001	-150,209
	Cost Increment	$43,300,000	$23,200,000	$1,476,299	-$21,400,000	-$29,400,000	-$44,300,000
	ICER	30	31	4	-245	-1029	591
**1-dose, 90%coverage (30EP/10WP)**	DALYs Averted	1,867,432	1,052,641	576,332	183,442	75,466	-83,744
	Cost Increment	$43,300,000	$23,200,000	$1,476,299	-$21,400,000	-$29,400,000	-$44,300,000
	ICER	29	29	4	-162	-639	1267

Table S19. DALYs averted over a 100-year time horizon, incremental costs over 100-years, and ICERS if Kenya switches from various two-dose scenarios (columns) to one-dose scenarios (rows), with different waning assumptions. Yellow signifies that the switch result into reduced cost and reduced DALYs averted, blue signifies that the switch would result into increased costs and increased DALYs averted, and green signifies that the switch would result into increased DALYs averted at lower costs.

		No Vaccination	2-dose, 31%coverage	2-dose, 50%coverage	2-dose, 70%coverage	2-dose, 77%coverage	2-dose, 90%coverage
		Median	Median	Median	Median	Median	Median
No Vaccination	DALYS Averted	0	-829397.50	-1315514.00	-1718444	-1829005	-1988002
	Cost Increment	$0	-$144,428,320	-$243,985,024	-$357,339,776	-$399,367,680	-$480,857,280
	ICER		171.17	182.27	203.83	213.81	236.081
2-dose, 31%coverage	DALYS Averted	829397.50	0	-486116.5	-883887.5	-989390	-1140014
	Cost Increment	$144,428,320	$0	-$99,738,560	-$213,093,312	-$255,121,216	-$336,610,816
	ICER	171.17		201.2473	234.34	249.25	282.55
2-dose, 50%coverage	DALYS Averted	1315514.00	486116.5	0	-395252.5	-500755	-651378.5
	Cost Increment	$243,985,024	$99,738,560	$0	-$113,354,752	-$155,382,656	-$236,783,040
	ICER	182.27	201.2473		274.30	294.73	341.21
2-dose, 70%coverage	DALYS Averted	1718444.0	883887.5	395252.5	0.0	-107257.5	-267078.5
	Cost Increment	$357,339,776	$213,093,312	$113,354,752	$0	-$41,974,528	-$122,498,816
	ICER	203.83	234.34	274.30		369.29	442.12
2-dose, 77%coverage	DALYS Averted	1829005	989390	500755	107257.5	0	-159821
	Cost Increment	$399,367,680	$255,121,216	$155,382,656	$41,974,528	$0	-$80,524,288
	ICER	213.81	249.25	294.73	369.29		493.59
2-dose, 90%coverage	DALYS Averted	1988002	1140014	651378.5	267078.5	159821	0
	Cost Increment	$480,857,280	$336,610,816	$236,783,040	$122,498,816	$80,524,288	$0
	ICER	236.08	282.55	341.21	442.12	493.59	
1-dose, 77%coverage	DALYS Averted	1731544	906631	417996	43525	-60913.5	-229956.5
	Cost Increment	$138,923,200	-$4,757,184	-$104,486,656	-$224,096,064	-$266,457,856	-$347,595,776
	ICER	78.52	-5.03	-230.50	-3928.61	4477.38	1529.083
1-dose, 70%coverage	DALYS Averted	1625570.00	795172.50	325207.50	-62662	-180545	-344841
	Cost Increment	$121,880,000	-$21,321,472	-$121,678,016	-$241,191,616	-$282,577,280	-$364,066,880
	ICER	74.3	-25.7	-362.5	3777.12	1564.675	1074.471
1-dose, 90%coverage	DALYS Averted	1887070	1072199	583564	207040.5	106800.5	-49639.5
	Cost Increment	$173,316,960	$28,456,576	-$68,994,048	-$187,920,192	-$231,852,032	-$313,827,136
	ICER	88.21	26.27	-114.26	-839.68	-2123.923	6519.192
1-dose, 90%coverage (15EP/20WP)	DALYS Averted	1630016	813222	340905.2	-56837.5	-173653.5	-326791.5
	Cost Increment	$192,827,072	$50,482,496	-$49,287,936	-$166,126,784	-$206,857,792	-$285,830,016
	ICER	117.59	60.37	-145.1789	2381.04	1216.12	908.08
1-dose, 90%coverage (20EP/20WP)	DALYS Averted	1767606	952906	464271	84606	-16946	-184965
	Cost Increment	$183,786,528	$39,262,720	-$59,576,000	-$178,086,784	-$220,696,192	-$302,185,792
	ICER	101.17	39.84	-123.01	-1998.88	2261.17	1654.124
1-dose, 90%coverage (25EP/20WP)	DALYS Averted	1836143	1021703	533067.5	160841	48702.5	-105595.5
	Cost Increment	$178,000,416	$33,029,952	-$64,820,992	-$183,996,480	-$227,167,168	-$308,656,768
	ICER	93.30	31.71	-116.98	-1107.42	-3787.04	2909.58
1-dose, 90%coverage (30EP/20WP)	DALYS Averted	1870361	1055736	567100.5	193460.2	93705.5	-68633.5
	Cost Increment	$174,989,984	$29,980,224	-$67,518,656	-$186,198,272	-$230,364,544	-$312,045,888
	ICER	89.92	28.11	-114.90	-915.71	-2500.77	4736.586
1-dose, 90%coverage (15EP/10WP)	DALYS Averted	1439535	621913	132839	-262413.5	-367916	-527245
	Cost Increment	$208,247,872	$65,158,624	-$35,556,416	-$149,091,904	-$191,119,808	-$272,609,408

	ICER	143.12	108.14	-246.33	587.09	538.02	525.77
**1-dose, 90%coverage (20EP/10WP)**	DALYS Averted	1678710	863996.5	380782	-2838	-117677.5	-276017
	Cost Increment	$190,303,552	$46,903,040	-$52,700,800	-$170,157,568	-$212,156,224	-$291,128,448
	ICER	112.20	52.70	-136.10	1709.28	1827.30	1087.495
**1-dose, 90%coverage (25EP/10WP)**	DALYS Averted	1800146	985934.5	497299.5	128500.2	18899.5	-147984
	Cost Increment	$181,255,776	$36,581,568	-$61,911,872	-$180,881,600	-$223,540,736	-$305,030,336
	ICER	97.54	35.96	-119.31	-1421.96	-5389.115	2085.597
**1-dose, 90%coverage (30EP/10WP)**	DALYS Averted	1859132	1044670	556034.5	184356.7	85873.8	-74696
	Cost Increment	$176,094,880	$30,987,200	-$66,543,680	-$185,513,408	-$229,286,144	-$310,775,744
	ICER	91.07	29.36	-115.38	-965.75	-2724.00	4307.366

ii. Full Incremental Cost Effectiveness Analysis Results

Table S20. Health and cost impact of vaccination strategies in Kenya if decision-makers consider strategies of vaccinating girls by age 10. These are the full CEA results expanded from Table 3, which includes total DALYs, DALYs averted, and all strategies ordered by increasing total cost.

One-dose durability	Strategy	Total cost (Million 2023 USD)	Total DALYs (Thousand)	Incremental cost (Million 2023 USD)	DALYs averted (Thousand)	ICER ($ per DALY averted)
Lifelong	No vaccination	414.46 (230.62-606.00)	6,467.38 (3,526.16-9,180.60)	-	-	-
	1-dose, 70% coverage	532.50 (411.14-676.31)	4,689.55 (2,634.16-6,757.53)	122.38 (70.8-181.01)	1,625.58 (892-2,423.09)	74.60 (29.41-203.58)
	1-dose, 77% coverage	546.33 (433.32-687.94)	4,569.15 (2,592.00-6,568.43)	16.84 (11.63-22.18)	111.46 (42.16-189.1)	142.78 (61.72-527.97)
	2-dose, 31% coverage	553.82 (403.54-730.33)	5,573.07 (3,039.92-8,036.84)	-	-	Dominated
	1-dose, 90% coverage	581.87 (475.74-713.27)	4,413.62 (2,531.41-6,270.33)	34.14 (25.33-42.58)	171.73 (60.6-298.11)	197.44 (85.15-702.24)
	2-dose, 50% coverage	650.86 (522.48-814.85)	5,052.00 (2,791.19-7,317.65)	-	-	Dominated
	2-dose, 70% coverage	769.83 (654.07-912.34)	4,645.83 (2,620.47-6,680.84)	-	-	Dominated
	2-dose, 77% coverage	809.38 (701.45-948.99)	4,541.81 (2,579.57-6,493.85)	-	-	Dominated
	2-dose, 90% coverage	885.85 (790.65-1,021.16)	4,391.11 (2,520.83-6,205.23)	313.33 (284-325.23)	49.64 (6.16-115.54)	6,508.8 (2,527.55-51,541.03)
Alternative waning (30EP/20WP)	No vaccination	414.46 (230.62-606.00)	6,467.38 (3,526.16-9,180.60)	-	-	-
	1-dose, 70% coverage	534.73 (411.73-680.77)	4,712.72 (2,640.58-6,805.54)	124.02 (75.71-181.1)	1,604.99 (885.57-2,375.06)	76.43 (31.67-205.15)
	1-dose, 77% coverage	548.46 (433.87-692.20)	4,590.55 (2,598.04-6,614.31)	16.71 (11.42-22.14)	112.74 (42.55-191.24)	140.23 (59.94-522.43)
	2-dose, 31% coverage	553.82 (403.54-730.33)	5,573.07 (3,039.92-8,036.84)	-	-	Dominated
	1-dose, 90% coverage	583.35 (476.25-716.95)	4,430.33 (2,536.90-6,310.30)	33.9 (24.76-42.49)	174.06 (61.14-304.01)	191.95 (81.65-695.31)
	2-dose, 50% coverage	650.86 (522.48-814.85)	5,052.00 (2,791.19-7,317.65)	-	-	Dominated
	2-dose, 70% coverage	769.83 (654.07-912.34)	4,645.83 (2,620.47-6,680.84)	-	-	Dominated
	2-dose, 77% coverage	809.38 (701.45-948.99)	4,541.81 (2,579.57-6,493.85)	-	-	Dominated
	2-dose, 90% coverage	885.85 (790.65-1,021.16)	4,391.11 (2,520.83-6,205.23)	312.05 (283.11-324.95)	68.63 (15.86-146.08)	4,736.59 (2,053.19-20,027.37)
	No vaccination	414.46 (230.62-606.00)	6,467.38 (3,526.16-9,180.60)	-	-	-

Pessimistic waning (20EP/10WP)	1-dose, 70% coverage	552.53 (417.44-714.83)	4,949.92 (2,713.50-7,235.11)	141.85 (120.03-187)	1,370.93 (807.36-1,827.14)	102.11 (66.41-232.15)
	2-dose, 31% coverage	553.82 (403.54-730.33)	5,573.07 (3,039.92-8,036.84)	-	-	Dominated
	1-dose, 77% coverage	566.41 (439.30-726.91)	4,824.51 (2,667.34-7,051.80)	16.58 (13.32-23.97)	115.94 (46.16-183.31)	137.18 (66.12-475.72)
	1-dose, 90% coverage	598.16 (481.21-751.26)	4,624.61 (2,600.13-6,741.83)	32.67 (24.36-41.91)	184.17 (67.21-309.98)	168.55 (78.8-626.06)
	2-dose, 50% coverage	650.86 (522.48-814.85)	5,052.00 (2,791.19-7,317.65)	-	-	Dominated
	2-dose, 70% coverage	769.83 (654.07-912.34)	4,645.83 (2,620.47-6,680.84)	-	-	Dominated
	2-dose, 77% coverage	809.38 (701.45-948.99)	4,541.81 (2,579.57-6,493.85)	-	-	Dominated
	2-dose, 90% coverage	885.85 (790.65-1,021.16)	4,391.11 (2,520.83-6,205.23)	291.13 (257.27-316.5)	276.02 (79.3-536.6)	1,087.49 (503.03-3,908.66)

Table S21. Health and cost impact of vaccination strategies in Kenya if decision-makers consider vaccinating girls by age 10 or expanding the HPV vaccination strategy to include catch-up or vaccination for all by age 10 using the 5-year cost savings of switching from a two- to one-dose schedule. These are the full CEA results expanded from Table 3, which includes total DALYs, DALYs averted, and all strategies ordered by increasing total cost.

One-dose durability	Strategy	Total cost (Million 2023 USD)	Total DALYs (Thousand)	Incremental cost (Million 2023 USD)	DALYs averted (Thousand)	ICER ($ per DALY averted)
Lifelong efficacy	No vaccination	414.46 (230.62-606.00)	6,467.38 (3,526.16-9,180.60)	-	-	-
	1-dose, 70% coverage	532.50 (411.14-676.31)	4,689.55 (2,634.16-6,757.53)	128.92 (99.49-183.61)	1,579.44 (839.55-2,061.36)	85.11 (48.29-236.32)
	1-dose, 77% coverage	546.33 (433.32-687.94)	4,569.15 (2,592.00-6,568.43)	-	-	Dominated
	2-dose, 31% coverage	553.82 (403.54-730.33)	5,573.07 (3,039.92-8,036.84)	-	-	Dominated
	1-dose, 11-24YO female catch-up	580.44 (482.29-701.29)	4,108.85 (2,356.00-5,833.02)	58.75 (39.37-135.52)	603.47 (278.16-3,347.58)	78.73 (28.64-256.28)
	1-dose, 90% coverage	581.87 (475.74-713.27)	4,413.62 (2,531.41-6,270.33)	-	-	Dominated
	1-dose, 11-19YO female catch-up	582.19 (483.80-703.48)	4,138.94 (2,378.70-5,864.55)	-	-	Dominated
	2-dose, 50% coverage	650.86 (522.48-814.85)	5,052.00 (2,791.19-7,317.65)	-	-	Dominated
	2-dose, 70% coverage	769.83 (654.07-912.34)	4,645.83 (2,620.47-6,680.84)	-	-	Dominated
	2-dose, 77% coverage	809.38 (701.45-948.99)	4,541.81 (2,579.57-6,493.85)	-	-	Dominated
	1-dose, vaccination for all by age 10	862.35 (771.21-983.84)	4,203.98 (2,438.65-5,868.82)	279.07 (279.07-279.07)	120.51 (120.51-120.51)	Weakly Dominated
	2-dose, 90% coverage	885.85 (790.65-1,021.16)	4,391.11 (2,520.83-6,205.23)	-	-	Dominated
Alternative waning (30EP/20WP)	No vaccination	414.46 (230.62-606.00)	6,467.38 (3,526.16-9,180.60)	-	-	-
	1-dose, 70% coverage	534.73 (411.73-680.77)	4,712.72 (2,640.58-6,805.54)	142.54 (112.35-192.57)	1,402.52 (720.28-1,889.22)	97.89 (59.68-348.11)
	1-dose, 77% coverage	548.46 (433.87-692.20)	4,590.55 (2,598.04-6,614.31)	-	-	Dominated
	2-dose, 31% coverage	553.82 (403.54-730.33)	5,573.07 (3,039.92-8,036.84)	-	-	Dominated

	1-dose, 11-24YO female catch-up	581.05 (482.42-703.09)	4,115.01 (2,357.46-5,852.30)	69.23 (44.66-159.32)	636.39 (283.12-3,328.31)	75.47 (29.35-250.21)
	1-dose, 11-19YO female catch-up	582.80 (483.94-705.29)	4,145.09 (2,380.17-5,883.79)	-	-	Dominated
	1-dose, 90% coverage	583.35 (476.25-716.95)	4,430.33 (2,536.90-6,310.30)	-	-	Dominated
	2-dose, 50% coverage	650.86 (522.48-814.85)	5,052.00 (2,791.19-7,317.65)	-	-	Dominated
	2-dose, 70% coverage	769.83 (654.07-912.34)	4,645.83 (2,620.47-6,680.84)	-	-	Dominated
	2-dose, 77% coverage	809.38 (701.45-948.99)	4,541.81 (2,579.57-6,493.85)	-	-	Dominated
	1-dose, vaccination for all by age 10	863.94 (771.74-986.70)	4,219.35 (2,444.50-5,900.54)	279.81 (279.81-279.81)	110.68 (110.68-110.68)	Weakly Dominated
	2-dose, 90% coverage	885.85 (790.65-1,021.16)	4,391.11 (2,520.83-6,205.23)	-	-	Dominated
Pessimistic waning (20EP/10WP)	No vaccination	414.46 (230.62-606.00)	6,467.38 (3,526.16-9,180.60)	-	-	-
	1-dose, 70% coverage	552.53 (417.44-714.83)	4,949.92 (2,713.50-7,235.11)	-	-	Dominated
	2-dose, 31% coverage	553.82 (403.54-730.33)	5,573.07 (3,039.92-8,036.84)	-	-	Dominated
	1-dose, 77% coverage	566.41 (439.30-726.91)	4,824.51 (2,667.34-7,051.80)	-	-	Dominated
	1-dose, 11-24YO female catch-up	590.69 (484.67-729.11)	4,233.94 (2,385.33-6,174.27)	182.22 (126.04-254.05)	2,066.75 (1,140.82-3,006.33)	86.16 (41.12-223.31)
	1-dose, 11-19YO female catch-up	592.52 (486.23-731.61)	4,265.01 (2,408.43-6,209.01)	-	-	Dominated
	1-dose, 90% coverage	598.16 (481.21-751.26)	4,624.61 (2,600.13-6,741.83)	-	-	Dominated
	2-dose, 50% coverage	650.86 (522.48-814.85)	5,052.00 (2,791.19-7,317.65)	-	-	Dominated
	2-dose, 70% coverage	769.83 (654.07-912.34)	4,645.83 (2,620.47-6,680.84)	-	-	Dominated
	2-dose, 77% coverage	809.38 (701.45-948.99)	4,541.81 (2,579.57-6,493.85)	-	-	Dominated
	1-dose, vaccination for all by age 10	879.21 (776.54-1,016.81)	4,387.00 (2,505.77-6,278.66)	285.63 (285.63-285.63)	30.59 (30.59-30.59)	Weakly Dominated
	2-dose, 90% coverage	885.85 (790.65-1,021.16)	4,391.11 (2,520.83-6,205.23)	-	-	Dominated

iii. One-Way Sensitivity Analysis

One-way sensitivity analyses were conducted for each scenario to assess the impact of changes in each economic parameter on ICERs using alternative estimates using range estimates extracted from published literature (Table 1). The following cost categories were investigated: Gavi contribution and costs of procurement, health system delivery, supplies, screening, and treatment. Using in-country expert input, Gavi contributions increased by 10% every year starting from a 30% contribution in 2023 (lower bound) or a 40% contribution in 2023 (upper bound) until the full cost of the vaccine was covered by the country. Results from one-way sensitivity analyses were presented using tornado diagrams to showcase changes in ICER for selected scenarios compared to the no vaccination scenario are presented on Figure S17, Tables S22 and S23 summarize all median ICERs and 95% CI of each scenario compared to the no vaccination scenario.

Figure S17. One Way sensitivity analyses of the ICER of selected scenarios compared to the no vaccination scenario

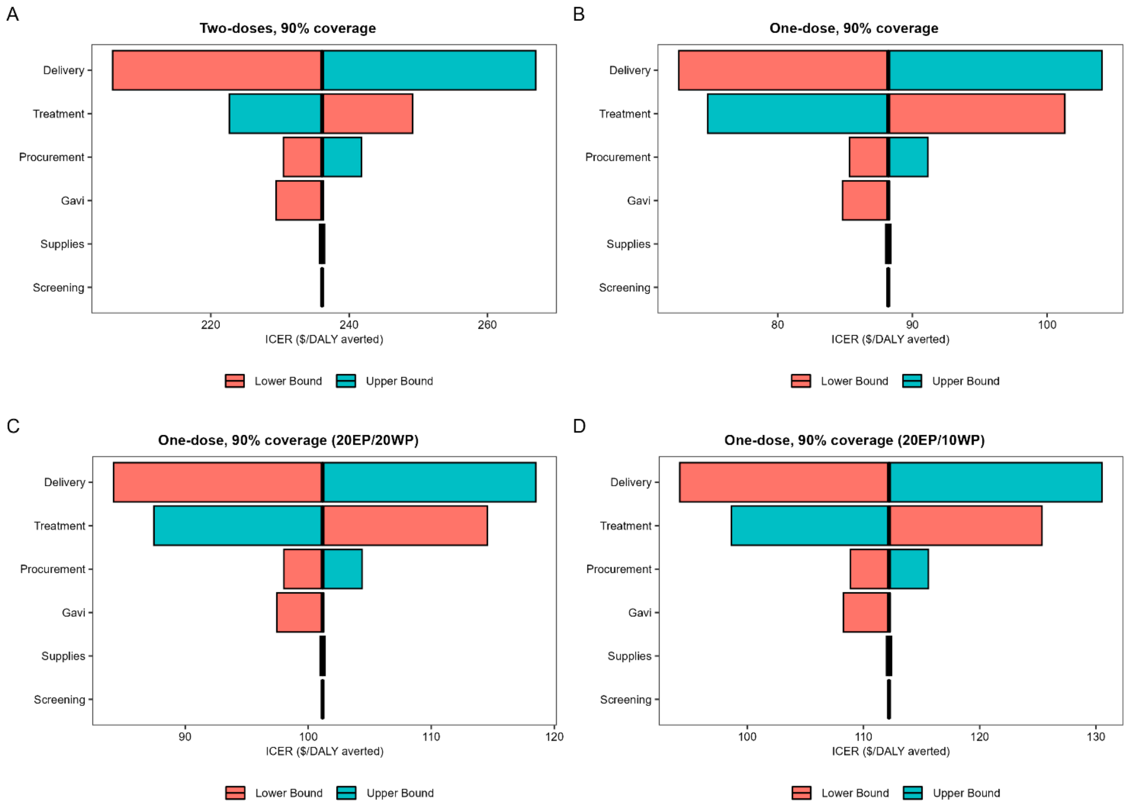

Table S22 Variation of the ICER estimates (median and 95% CI) comparing each scenario to the no vaccination scenario after adjusting GAVI contribution, Procurement and Delivery Costs. These are the full one-way sensitivity analysis expanded from Figure S17.

**One-dose durability**	**Strategy**	**Initial Run**	**GAVI Contribution**		**Procurement**		**Delivery**	
			**Lower Bound**	**Upper Bound**	**Lower Bound**	**Upper Bound**	**Lower Bound**	**Upper Bound**
Lifelong efficacy	No vaccination	-	-	-	-	-	-	-
	2-dose 31% cov	171.2 (108.8-356.3)	166.2 (105.1-347.4)	170.4 (108.2-354.9)	166.7 (105.4-348.3)	175.8 (112.2-364.5)	147.9 (91.4-314.9)	194.9 (126.6-398.6)
	2-dose 50% cov	182.3 (112.2-398)	176.9 (108.3-388)	182 (112.1-397.6)	177.6 (108.9-389.4)	187 (115.7-406.9)	157.6 (94.3-351.9)	207.5 (130.6-445.1)
	2-dose 70% cov	203.8 (122.7-468.7)	197.9 (118.6-457.1)	203.8 (122.7-468.8)	198.8 (119.2-458.8)	208.9 (126.4-478.9)	176.9 (103.6-415.3)	231.4 (142.3-523.3)
	2-dose 77% cov	213.8 (127.9-498.7)	207.7 (123.6-486.4)	213.9 (127.9-498.8)	208.6 (124.2-488.2)	219.1 (131.6-509.4)	185.8 (108.2-442.2)	242.4 (147.9-556.3)
	2-dose 90% cov	236.1 (139.8-558.5)	229.5 (135.2-544.8)	236.3 (139.9-558.9)	230.5 (135.9-547)	241.8 (143.7-570.3)	205.8 (118.9-496)	267 (161.1-622.3)
	1-dose 77% cov	78.5 (31.6-217.7)	75.3 (29.4-211.4)	78.5 (31.6-217.7)	75.8 (29.7-212.4)	81.3 (33.5-223.1)	63.7 (21.5-189.1)	93.6 (41.9-246.8)
	1-dose 70% cov	74.3 (29.2-203)	71.2 (27.1-197.1)	74.3 (29.2-203)	71.6 (27.4-198)	77 (31.1-208.2)	60 (19.4-176)	88.9 (39.3-230.6)
	1-dose 90% cov	88.2 (37.1-247.2)	84.8 (34.7-240.3)	88.3 (37.1-247.3)	85.3 (35.1-241.4)	91.1 (39.1-253.1)	72.7 (26.4-215.6)	104.1 (47.9-279.4)

	1-dose, vaccination for all by age 10	204.7 (114.4-498.4)	198.7 (110.3-485.9)	205.1 (114.6-499.2)	199.7 (111-488)	209.8 (117.8-509)	177.4 (95.9-441.5)	232.5 (133.2-556.4)
	1-dose, 11-19YO female catch-up	76.7 (29.6-221.3)	73.4 (27.3-214.7)	77.4 (30.1-222.7)	74.1 (27.8-216.1)	79.3 (31.4-226.6)	61.9 (19.4-191.6)	91.7 (40-251.6)
	1-dose, 11-24YO female catch-up	74.9 (28.6-215.7)	71.7 (26.4-209.2)	75.6 (29.1-217.1)	72.4 (26.9-210.6)	77.5 (30.4-220.9)	60.3 (18.6-186.5)	89.8 (38.9-245.4)
	1-dose, 31% cov	59.7 (25.8-149.1)	57 (23.8-144.5)	59.2 (25.4-148.3)	57.3 (24-145)	62.1 (27.6-153.3)	47.3 (16.5-127.9)	72.3 (35.2-170.8)
	1-dose, 50% cov	64 (25-168.8)	61.1 (23-163.7)	63.8 (24.9-168.5)	61.5 (23.3-164.4)	66.5 (26.8-173.3)	51 (15.8-145.3)	77.2 (34.5-192.8)
Alternative waning (30EP/20WP)	1-dose 90% cov	89.9 (38.9-249.1)	86.5 (36.5-242.1)	90 (38.9-249.2)	87 (36.9-243.2)	92.9 (40.9-255)	74.2 (28-217.4)	105.9 (49.9-281.4)
	1-dose, 11-19YO female catch-up	77.2 (30.3-221.7)	73.9 (28-215.1)	77.9 (30.8-223.1)	74.6 (28.5-216.5)	79.9 (32.1-227)	62.4 (20.1-191.9)	92.4 (40.7-252.1)
	1-dose, vaccination for all by age 10	206.9 (116.3-501.6)	200.8 (112.2-489)	207.3 (116.6-502.4)	201.9 (112.9-491.2)	212 (119.8-512.3)	179.4 (97.7-444.4)	234.9 (135.4-559.9)
	1-dose, 11-24YO female catch-up	75.5 (29.3-216.1)	72.2 (27.1-209.6)	76.2 (29.8-217.5)	72.9 (27.6-211)	78.1 (31.2-221.3)	60.8 (19.2-186.9)	90.4 (39.7-245.9)
	1-dose, 70% cov	76.4 (31.7-205.2)	73.3 (29.5-199.2)	76.4 (31.7-205.1)	73.7 (29.8-200.1)	79.2 (33.6-210.3)	62 (21.6-177.9)	91.2 (41.9-232.9)
	1-dose, 77% cov	80.9 (33.8-219.7)	77.6 (31.5-213.4)	80.9 (33.8-219.7)	78.1 (31.9-214.4)	83.7 (35.7-225.1)	65.9 (23.5-191)	96.2 (44.3-249)
Pessimistic waning (20EP/10WP)	1-dose 90% cov	112.2 (59.8-271.4)	108.3 (57-264)	112.3 (59.8-271.6)	108.9 (57.4-265.2)	115.6 (62.2-277.8)	94.2 (47-237.5)	130.5 (72.7-306)
	1-dose, 11-19YO female catch-up	88.4 (42.4-229.3)	84.8 (39.9-222.5)	89.1 (43-230.8)	85.5 (40.5-224)	91.2 (44.5-234.8)	72.2 (31.1-198.8)	104.8 (54-260.5)
	1-dose, vaccination for all by age 10	232.8 (141.8-536.4)	226.2 (137.2-523.1)	233.2 (142.1-537.3)	227.3 (138-525.4)	238.4 (145.7-547.7)	202.7 (120.7-475.8)	263.4 (163.3-598.3)
	1-dose, 11-24YO female catch-up	86.2 (41.1-223.3)	82.6 (38.6-216.6)	86.9 (41.7-224.7)	83.4 (39.2-218.1)	89 (43.1-228.6)	70.3 (29.9-193.4)	102.4 (52.6-253.8)
	1-dose, 70% cov	100.3 (58-230.5)	96.7 (55.4-224.1)	100.2 (58-230.5)	97.2 (55.8-225)	103.4 (60.4-236.2)	83.7 (45.7-200.9)	117.1 (70.6-260.8)
	1-dose, 77% cov	103.5 (58.1-243.7)	99.8 (55.4-236.9)	103.5 (58.1-243.7)	100.3 (55.8-237.9)	106.7 (60.4-249.6)	86.5 (45.7-212.7)	120.9 (70.7-275.4)
15EP/20WP	1-dose 90% cov	117.6 (64.9-278)	113.5 (62-270.4)	117.7 (64.9-278.1)	114.2 (62.4-271.6)	121.1 (67.4-284.5)	99.1 (51.6-243.5)	136.5 (78.4-313.2)
20EP/20WP	1-dose 90% cov	101.2 (49.4-260.9)	97.5 (46.8-253.7)	101.2 (49.5-261.1)	98 (47.2-254.8)	104.4 (51.6-267.1)	84.2 (37.6-228)	118.5 (61.4-294.4)
25EP/20WP	1-dose 90% cov	93.3 (42.3-252.9)	89.8 (39.8-245.9)	93.4 (42.3-253.1)	90.3 (40.2-247)	96.4 (44.4-259)	77.3 (31.2-220.9)	109.9 (53.6-285.7)
15EP/10WP	1-dose 90% cov	143.1 (92.2-305.8)	138.5 (88.8-297.7)	143.2 (92.3-306)	139.3 (89.3-298.9)	147.1 (95.1-312.8)	122.2 (76.5-268.6)	164.5 (108.2-343.8)
25EP/10WP	1-dose 90% cov	97.5 (46.3-257)	93.9 (43.7-249.9)	97.6 (46.3-257.2)	94.5 (44.1-251)	100.7 (48.4-263.1)	80.9 (34.8-224.5)	114.5 (58-290.1)
30EP/10WP	1-dose 90% cov	91.1 (40.1-250.3)	87.6 (37.7-243.4)	91.1 (40.1-250.5)	88.2 (38.1-244.4)	94.1 (42.1-256.3)	75.3 (29.2-218.5)	107.2 (51.2-282.8)
Lifelong efficacy + change in testing, treatment strategy	1-dose,90%cov HPV DNA	88.1 (37-246.8)	84.7 (34.7-239.9)	88.1 (37-247)	85.2 (35-241)	91 (39-252.8)	72.5 (26.3-215.3)	103.9 (47.9-279)
	1-dose,90%cov HPV DNA + ablative therapy	86.8 (36.5-243.8)	83.5 (34.2-237)	86.9 (36.5-243.9)	84 (34.5-238)	89.7 (38.5-249.7)	71.4 (25.9-212.6)	102.5 (47.3-275.6)

	1-dose, 90%cov unbound efficacy	84.4 (36.7-252.1)	81.1 (34.4-245.1)	84.4 (36.7-252.3)	81.6 (34.7-246.2)	87.2 (38.7-258.1)	69.2 (26.1-220.1)	99.9 (47.5-284.8)

Results are summarized as a median and 95% CI of all 25 parameter sets.

Table S23 Variation of the ICER estimates (median and 95% CI) comparing each scenario to the no vaccination scenario after adjusting Supplies, Screening and Treatment Costs. These are the full one-way sensitivity analysis expanded from Figure S17.

One-dose durability	Strategy	Initial Run	Supplies		Screening		Treatment	
			Lower Bound	Upper Bound	Lower Bound	Upper Bound	Lower Bound	Upper Bound
Lifelong efficacy	No vaccination	-	-	-	-	-	-	-
	2-dose 31% cov	171.2 (108.8-356.3)	170.9 (108.6-355.8)	171.4 (109-356.8)	171.2 (108.8-356.4)	171.1 (108.8-356.3)	184.3 (122.4-369.8)	157.7 (94.9-342.6)
	2-dose 50% cov	182.3 (112.2-398)	182 (112-397.5)	182.6(112.5-398.6)	182.3 (112.3-398)	182.3 (112.2-398)	195.4 (125.8-411.5)	168.9 (98.4-384.2)
	2-dose 70% cov	203.8 (122.7-468.7)	203.5 (122.5-468.1)	204.2 (123-469.4)	203.9 (122.7-468.8)	203.8 (122.7-468.7)	216.9 (136.2-482.2)	190.4 (108.9-455)
	2-dose 77% cov	213.8 (127.9-498.7)	213.5 (127.6-498)	214.1(128.1-499.4)	213.8 (127.9-498.7)	213.8 (127.8-498.7)	226.9 (141.4-512.1)	200.4 (114-484.9)
	2-dose 90% cov	236.1 (139.8-558.5)	235.7 (139.5-557.8)	236.4 (140-559.3)	236.1 (139.8-558.5)	236.1 (139.7-558.5)	249.2 (153.3-572)	222.7 (125.9-544.7)
	1-dose 77% cov	78.5 (31.6-217.7)	78.3 (31.4-217.3)	78.7 (31.7-218)	78.5 (31.6-217.7)	78.5 (31.5-217.7)	92.1 (45.1-231.1)	64.6 (17.7-203.9)
	1-dose 70% cov	74.3 (29.2-203)	74.1 (29.1-202.7)	74.5 (29.3-203.3)	74.3 (29.2-203)	74.3 (29.2-203)	87.7 (42.7-216.5)	60.6 (15.4-189.2)
	1-dose 90% cov	88.2 (37.1-247.2)	88 (36.9-246.8)	88.4 (37.2-247.6)	88.2 (37.1-247.2)	88.2 (37-247.2)	101.3 (50.6-260.6)	74.8 (23.2-233.4)
	1-dose, vaccination for all by age 10	204.7 (114.4-498.4)	204.3 (114.1-497.7)	205 (114.6-499.1)	204.7 (114.4-498.4)	204.7 (114.3-498.4)	217.8 (127.9-511.9)	191.3 (100.5-484.6)
	1-dose, 11-19YO female catch-up	76.7 (29.6-221.3)	76.5 (29.5-220.9)	76.8 (29.7-221.7)	76.7 (29.6-221.3)	76.6 (29.6-221.3)	89.7 (43.1-234.7)	63.3 (15.8-207.6)
	1-dose, 11-24YO female catch-up	74.9 (28.6-215.7)	74.7 (28.5-215.3)	75.1 (28.8-216)	74.9 (28.7-215.7)	74.9 (28.6-215.7)	88 (42.1-229.1)	61.5 (14.8-202)
	1-dose, 31% cov	59.7 (25.8-149.1)	59.5 (25.6-148.8)	59.8 (25.9-149.4)	59.7 (25.8-149.1)	59.7 (25.7-149.1)	72.8 (39.1-162.6)	46.2 (12.1-135.3)
	1-dose, 50% cov	64 (25-168.8)	63.8 (24.9-168.5)	64.1 (25.2-169.1)	64 (25.1-168.8)	63.9 (25-168.8)	77.1 (38.4-182.3)	50.5 (11.4-155)
Alternative waning (30EP/20WP)	1-dose 90% cov	89.9 (38.9-249.1)	89.7 (38.7-248.7)	90.1 (39-249.4)	89.9 (38.9-249.1)	89.9 (38.8-249)	103 (52.3-262.5)	76.6 (25.1-235.3)
	1-dose, 11-19YO female catch-up	77.2 (30.3-221.7)	77.1 (30.2-221.3)	77.4 (30.4-222.1)	77.3 (30.3-221.7)	77.2 (30.3-221.7)	90.3 (43.8-235.1)	63.9 (16.5-208)
	1-dose, vaccination for all by age 10	206.9 (116.3-501.6)	206.6 (116.1-500.9)	207.2(116.6-502.3)	206.9 (116.4-501.6)	206.9 (116.3-501.6)	220 (129.8-515)	193.5 (102.5-487.8)
	1-dose, 11-24YO female catch-up	75.5 (29.3-216.1)	75.3 (29.2-215.7)	75.6 (29.5-216.4)	75.5 (29.4-216.1)	75.5 (29.3-216.1)	88.5 (42.8-229.5)	62.1 (15.6-202.3)
	1-dose, 70% cov	76.4 (31.7-205.2)	76.3 (31.6-204.8)	76.6 (31.8-205.5)	76.5 (31.7-205.2)	76.4 (31.7-205.1)	89.8 (45.1-218.6)	62.8 (17.9-191.4)
	1-dose, 77% cov	80.9 (33.8-219.7)	80.7 (33.7-219.3)	81 (33.9-220)	80.9 (33.8-219.7)	80.8 (33.8-219.7)	94.4 (47.2-233.1)	67 (20-205.9)

Pessimistic waning (20EP/10WP)	1-dose 90% cov	112.2 (59.8-271.4)	112 (59.6-271)	112.4(59.9-271.8)	112.2 (59.8-271.4)	112.2 (59.7-271.4)	125.4 (72.9-284.7)	98.6 (46.3-257.8)
	1-dose, 11-19YO female catch-up	88.4 (42.4-229.3)	88.2 (42.3-229)	88.6 (42.6-229.7)	88.4 (42.5-229.4)	88.3 (42.4-229.3)	101.7 (55.7-242.7)	74.7 (28.9-215.7)
	1-dose, vaccination for all by age 10	232.8 (141.8-536.4)	232.4 (141.6-535.7)	233.1(142.1-537.2)	232.8 (141.8-536.5)	232.7 (141.8-536.4)	245.7 (155.1-549.8)	219.5 (128.3-522.8)
	1-dose, 11-24YO female catch-up	86.2 (41.1-223.3)	86 (41-222.9)	86.4 (41.3-223.7)	86.2 (41.1-223.3)	86.1 (41.1-223.3)	99.5 (54.4-236.7)	72.5 (27.6-209.6)
	1-dose, 70% cov	100.3 (58-230.5)	100.1 (57.9-230.2)	100.5(58.2-230.9)	100.3 (58.1-230.6)	100.2 (58-230.5)	113 (70.8-243.8)	87.2 (45-217)
	1-dose, 77% cov	103.5 (58.1-243.7)	103.3 (57.9-243.3)	103.7(58.2-244.1)	103.5 (58.1-243.7)	103.5 (58.1-243.7)	116.6 (70.8-257)	90.1 (45-230.1)
15EP/20WP	1-dose 90% cov	117.6 (64.9-278)	117.4 (64.7-277.6)	117.8 (65-278.4)	117.6 (64.9-278)	117.6 (64.8-278)	130.7 (78-291.3)	104.1 (51.6-264.4)
20EP/20WP	1-dose 90% cov	101.2 (49.4-260.9)	101 (49.3-260.5)	101.4(49.6-261.3)	101.2 (49.4-260.9)	101.1 (49.4-260.9)	114.6 (62.7-274.3)	87.5 (35.8-247.2)
25EP/20WP	1-dose 90% cov	93.3 (42.3-252.9)	93.1 (42.1-252.6)	93.5 (42.4-253.3)	93.3 (42.3-253)	93.3 (42.3-252.9)	106.8 (55.7-266.3)	80 (28.6-239.2)
15EP/10WP	1-dose 90% cov	143.1 (92.2-305.8)	142.9 (92-305.4)	143.4(92.4-306.3)	143.1 (92.2-305.8)	143.1 (92.2-305.8)	156.1 (104.8-319)	129.8 (79.3-292.3)
25EP/10WP	1-dose 90% cov	97.5 (46.3-257)	97.3 (46.1-256.6)	97.7 (46.4-257.4)	97.6 (46.3-257)	97.5 (46.2-257)	110.9 (59.6-270.4)	83.8 (32.6-243.3)
30EP/10WP	1-dose 90% cov	91.1 (40.1-250.3)	90.9 (40-249.9)	91.3 (40.2-250.7)	91.1 (40.1-250.3)	91.1 (40.1-250.3)	104.3 (53.5-263.7)	77.7 (26.4-236.6)
Lifelong efficacy + change in testing, treatment strategy	1-dose,90%cov HPV DNA	88.1 (37-246.8)	87.9 (36.9-246.4)	88.2 (37.1-247.2)	88.1 (37-246.8)	88 (37-246.8)	101.2 (50.5-260.3)	74.7 (23.2-233.1)
	1-dose,90%cov HPV DNA + ablative therapy	86.8 (36.5-243.8)	86.6 (36.4-243.4)	87 (36.6-244.2)	86.8 (36.5-243.8)	86.8 (36.5-243.8)	99.9 (50-257.2)	73.5 (22.7-230.1)
	1-dose,90%cov unbound efficacy	84.4 (36.7-252.1)	84.2 (36.6-251.7)	84.6 (36.8-252.5)	84.4 (36.7-252.1)	84.4 (36.7-252.1)	97.5 (50.2-265.6)	71 (22.9-238.3)

Results are summarized as a median and 95% CI of all 25 parameter sets.

V. Differential equations

We use a system of differential equations to estimate changes in population and infection dynamics over each time step. We split the full system of equations into topic-based modules and solve each iteratively using a 4th-order Runge-Kutta numerical solver in MATLAB. The order is largely historical and based on the work of (Tan et al., 2018)[50]. The modules are:

1. HPV natural history
    a. Progression and clearance of HPV
    b. Progression and regression of precancerous lesions
    c. Development and progression of cervical cancer
    d. Cervical cancer- associated mortality
2. Cervical cancer symptomatic diagnosis, screening, and treatment
    a. Symptomatic diagnosis
    b. Screening
    c. Treatment
3. HPV and HIV transmission
    a. Heterosexual mixing by gender, age, and risk group
    b. Partnership adjustment
    c. HPV infection by type
    d. HIV infection
4. HIV natural history and treatment
    a. CD4 progression
    b. Viral load progression
    c. ART initiation, discontinuation, and scale-up by CD4 count
    d. HIV-associated mortality
5. Demography
    a. Births
    b. Mother-to-child HIV transmission
    c. Aging and risk-group redistribution
    d. Natural deaths
6. Male circumcision
7. HPV vaccination
    a. School-based regimen
    b. Catch-up regimen

Throughout each simulation, we track population demographics and the number of persons with infection, with progressed disease, or with preventative or therapeutic treatment. We describe these states $X_{g,a,r}^{d,v,h,s,x,p}(t)$ with the following indices (using 1-based indexing):

Index	Description	Values
d	HIV disease state, CD4 count, circumcision status, and ART status	1. HIV-negative, uncircumcised<br>2. HIV-negative, circumcised<br>3. HIV-positive, acute infection<br>4. HIV-positive, CD4 > 500 cells/μL<br>5. HIV-positive, CD4 350-500 cells/μL<br>6. HIV-positive, CD4 200-350 cells/μL<br>7. HIV-positive, CD4 ≤ 200 cells/μL<br>8. HIV-positive, on ART
v	HIV viral load	1. If ($2 < d < 8$), Acute infection; if ($d = 1,2$), HIV-negative: VL = 0.0<br>2. Asymptomatic: VL = 3.0-4.5 $\log_{10}$<br>3. Pre-AIDS symptomatic: VL = 4.0-5.5 $\log_{10}$<br>4. AIDS: VL = 5.5-7.0 $\log_{10}$<br>5. Late-stage<br>6. On ART and virally suppressed: VL = 0.0
h	Vaccine-type HPV precancer or disease state	1. Susceptible<br>2. Infected<br>3. CIN1<br>4. CIN2<br>5. CIN3<br>6. Cervical cancer or hysterectomy<br>7. Immune
s	Non-vaccine type HPV precancer or disease state	1. Susceptible

		2. Infected<br>3. CIN1<br>4. CIN2<br>5. CIN3<br>6. Cervical cancer or hysterectomy<br>7. Immune
x	Cervical cancer or hysterectomy status	1. If (h = 6 or s = 6), Cervical cancer, local, undiagnosed; else, no cancer or hysterectomy<br>2. Cervical cancer, regional, undiagnosed<br>3. Cervical cancer, distant, undiagnosed<br>4. Cervical cancer, local, diagnosed & untreated<br>5. Cervical cancer, regional, diagnosed & untreated<br>6. Cervical cancer, distant, diagnosed & untreated<br>7. Cervical cancer, local, treated<br>8. Cervical cancer, regional, treated<br>9. Cervical cancer, distant, treated<br>10. Hysterectomy
p	Vaccination and screening history	1. Non-vaccinated, non-screened<br>2. Vaccinated<br>3. Screened<br>4. Vaccinated and screened
g	Gender	1. Male<br>2. Female
a	Age	1. 0-4<br>2. 5-9<br>3. 10-14<br>4. 15-19<br>5. 20-24<br>6. 25-29<br>7. 30-34<br>8. 35-39<br>9. 40-44<br>10. 45-49<br>11. 50-54<br>12. 55-59<br>13. 60-64<br>14. 65-69<br>15. 70-74<br>16. 75-79
r	Risk	1. Low risk<br>2. Medium risk<br>3. High risk

a. Demography

**Equation variables**	
γ_a^d(t)	The annual fertility rate for females by age *a* and HIV disease stage *d*.<br>Women ages 15-49 bear children.
$\eta(t)$	The proportion of births from HIV-positive females that result in vertical transmission.
$b\_s(t)$	Number of births by women without HIV and women on ART.
$b\_i(t)$	Number of births by women living with HIV.
$b_{g,a,r}^{d,1,h,s,x,p}(t)$	Number of infant births of HIV disease stage *d* and gender *g*.<br>We assume an equal gender ratio at birth of 1:1, that all newborns are born as low risk, no vertical transmission of HPV, and that if HIV is vertically transmitted, that infected newborns are born into the acute stage of HIV.
$\phi_{g,a,r}$	Distribution of sexual risk *r* by gender *g* and age *a*. (Currently, the risk distribution derived from male partner data is used for both males and females for simplicity.)
$\mu\_bkrd_{g,a}(t)$	Annual background mortality rate by gender *g* and age *a*.

Fertility

The number of births by HIV status of the mother are calculated as:

HIV-negative women and women on ART

$$b\_s(t) = \sum_{h=1}^{7}\sum_{s=1}^{7}\sum_{x=1}^{3}\sum_{p=1}^{4}\sum_{a=4}^{10}\sum_{r=1}^{3}\left[\gamma_a^1(t)\cdot X_{g,a,r}^{1,1,h,s,x,p}(t) + \gamma_a^8(t)\cdot X_{g,a,r}^{8,6,h,s,x,p}(t)\right]$$

Women living with HIV

$$b\_i(t) = \sum_{d=3}^{7}\sum_{v=1}^{5}\sum_{h=1}^{7}\sum_{s=1}^{7}\sum_{x=1}^{3}\sum_{p=1}^{4}\sum_{a=4}^{10}\sum_{r=1}^{3}\left[\gamma_a^d(t)\cdot X_{g,a,r}^{d,v,h,s,x,p}(t)\right]$$

We then compute the number of births by gender and HIV status of the infant as:

HIV-negative, uncircumcised births

For h = s = x = p = a = r = 1,

$$b_{g,a,r}^{1,1,h,s,x,p}(t) = 0.5\,\left(b\_s(t) + \left(1-\eta(t)\right)b\_i(t)\right)$$

else,

$$b_{g,a,r}^{1,1,h,s,x,p}(t) = 0$$

HIV-positive births

For h = s = x = p = a = r = 1,

$$b_{g,a,r}^{3,1,h,s,x,p}(t) = 0.5\,\eta(t)\cdot b\_i(t)$$

else,

$$b_{g,a,r}^{3,1,h,s,x,p}(t) = 0$$

Aging

To age the population, one-fifth of each compartment moves to the next age group while maintaining the same gender, disease state, and sexual risk distribution $\phi_{g,a,r}$:

$$\frac{dX_{g,1,r}^{d,v,h,s,x,p}(t)}{dt} = -\frac{1}{5}\sum_{r=1}^{3} X_{g,1,r}^{d,v,h,s,x,p}(t)\,\cdot\,\phi_{g,a,r} \qquad (for\ a = 1)$$

$$\frac{dX_{g,a,r}^{d,v,h,s,x,p}(t)}{dt} = -\frac{1}{5}\sum_{r=1}^{3} X_{g,a,r}^{d,v,h,s,x,p}(t)\,\cdot\,\phi_{g,a,r} + \frac{1}{5}\sum_{r=1}^{3} X_{g,a-1,r}^{d,v,h,s,x,p}(t)\,\cdot\,\phi_{g,a-1,r} \qquad (for\ a \neq 1)$$

Upon aging to the next five-year group, individuals are re-distributed into the closest unfilled risk group to match observed data on the age distribution of low, medium, and high-risk individuals.

Mortality

We compute the number of deaths due to background mortality as:

$$\frac{dX_{g,a,r}^{d,v,h,s,x,p}(t)}{dt} = -\mu\_bkrd_{g,a}(t)\cdot X_{g,a,r}^{d,v,h,s,x,p}(t)$$

b. Sexual Behavior

Mixing matrix

**Equation variables**	
$c_{g,a,r}$	Number of partners a person has per year of gender *g,* age *a,* and sexual-risk group *r* (ie. the partner exchange rate, or contact rate).
ϵ_a	Mixing parameter by age *a.*<br>We assume a mixing pattern that is partially random and partially off-diagonal (0 < ϵ_a < 1), where ($\epsilon_a = 0$) indicates completely off-diagonal mixing, and ($\epsilon_a = 1$) indicates completely random mixing.
ϵ_r	Mixing parameter by sexual-risk group *r.*<br>We assume a mixing pattern that is partially random and partially on-diagonal (0 < ϵ_r < 1), where ($\epsilon_r = 0$) indicates completely on-diagonal mixing, and ($\epsilon_r = 1$) indicates completely random mixing.
$\delta_{g,a,a'}$	Mixing pattern by age.<br>In completely non-random mixing by age, females are most likely to form partnerships with males of the next oldest age group. We represent this pattern using on off-diagonal matrix.<br><br>For males (*g* = 1) of age *a* mixing with females of age *a'*:<br>= 0.3 if ($a = a'$)<br>= 0.7 if ($a = a' + 1$)<br>except for the following (correct for no sexual activity before age group 3):<br>= 0.0 if ($a = a' = 1$)<br>= 0.0 if ($a = 2$) and (*a'* = 1)<br>= 0.0 if ($a = 2$) and (*a'* = 2)<br>= 0.0 if ($a = 3$) and (*a'* = 2)<br><br>For females (*g* = 2) of age *a* mixing with males of age *a'*:

	= 0.3 if $(a = a')$<br>= 0.7 if $(a = a' - 1)$<br>except for the following (correct for no sexual activity before age group 3):<br>= 0.0 if $(a = a' = 1)$<br>= 0.0 if $(a = 1)$ and $(a' = 2)$<br>= 0.0 if $(a = a' = 2)$<br>= 0.0 if $(a = 2)$ and $(a' = 3)$
$\delta_{r,r'}$	Mixing pattern by risk.<br>Completely non-random mixing by risk confines sexual encounters to individuals within the same risk group. We represent this pattern using an identity matrix.<br>= 1.0 if $(r = r')$<br>= 0.0 if $(r \neq r')$

For a person of gender *g,* age *a,* and sexual-risk group *r,* we use the mixing matrix $\rho_{g,a,a',r,r'}(t)$ to describe the proportion of sexual partners that come from age group *a'* and sexual-risk group *r'*. We assume that mixing is partially random and partially designated by a mixing pattern $\delta_{g,a,a'}$ or $\delta_{r,r'}$. The overall mixing matrix is therefore a weighted average of random mixing proportional to the number of available partnerships of each group, and mixing among groups with similar characteristics. Although an off-diagonal mixing pattern results in the first and last ages groups (ages 10-14 and 75-79) having fewer than 100% of their partnerships, these age groups have relatively few partnerships and contribute marginally to overall infection transmission.

$$\rho_{g,a,a',r,r'}(t) = \left(\epsilon_a \cdot \frac{\sum_{r'=1}^{3}\left(c_{g',a',r'} \cdot \sum_{d'=1}^{8}\sum_{v'=1}^{6}\sum_{h'=1}^{7}\sum_{s'=1}^{7}\sum_{x'=1}^{4}\sum_{p'=1}^{4} X_{g',a',r'}^{d',v',h',s',x',p'}(t)\right)}{\sum_{a'=1}^{16}\sum_{r'=1}^{3}\left(c_{g',a',r'} \cdot \sum_{d'=1}^{8}\sum_{v'=1}^{6}\sum_{h'=1}^{7}\sum_{s'=1}^{7}\sum_{x'=1}^{4}\sum_{p'=1}^{4} X_{g',a',r'}^{d',v',h',s',x',p'}(t)\right)} + (1-\epsilon_a)\delta_{g,a,a'} \right) \cdot \left(\epsilon_r \cdot \frac{\left(c_{g',a',r'} \cdot \sum_{d'=1}^{8}\sum_{v'=1}^{6}\sum_{h'=1}^{7}\sum_{s'=1}^{7}\sum_{x'=1}^{4}\sum_{p'=1}^{4} X_{g',a',r'}^{d',v',h',s',x',p'}(t)\right)}{\sum_{r'=1}^{3}\left(c_{g',a',r'} \cdot \sum_{d'=1}^{8}\sum_{v'=1}^{6}\sum_{h'=1}^{7}\sum_{s'=1}^{7}\sum_{x'=1}^{4}\sum_{p'=1}^{4} X_{g',a',r'}^{d',v',h',s',x',p'}(t)\right)} + (1-\epsilon_r)\delta_{g,r,r'} \right)$$

Rate of partner change

Equation variables	
$c_{g,a,r}$	Number of partners a person has per year of gender *g,* age *a,* and sexual-risk group *r* (ie. the partner exchange rate, or contact rate).<br>We assume zero partnerships for individuals below the age of sexual debut (age 10).
θ	Gender influence on contact rate adjustment.<br>We assume an adjusted contact rate equally driven by rates reported by males and females (θ = 0.5), where (θ = 0) when completely female-driven, and (θ = 1) when completely male-driven.
$\rho_{g,a,a',r,r'}(t)$	Mixing matrix for a person of gender *g,* age *a,* and sexual-risk group *r* that describes the proportion of sexual partners that come from age group *a'* and sexual-risk group *r'*.<br>We assume a solely heterosexual population and therefore that all contacts are with the opposite gender.

Bias in observed data leads to contact rates $c_{g,a,r}$ that, when assuming solely heterosexual contact, are inconsistent between males and females. We account for this variability by using an adjusted contact rate $c^{*}_{g,a,a',r,r'}(t)$ that ensures that the number of partnerships of males of age *a* and risk group *r* with females of age *a'* and risk group *r'* equals the number of partnerships of females of age *a* and risk group *r* with males of age *a'* and risk group *r'*.

We first calculate the discrepancy between reported male and female contacts as:

$$B_{a,a',r,r'}(t) = \frac{c_{1,a,r} \cdot \rho_{1,a,a',r,r'}(t) \cdot \sum_{d'=1}^{8}\sum_{v'=1}^{6}\sum_{h'=1}^{7}\sum_{s'=1}^{7}\sum_{x'=1}^{4}\sum_{p'=1}^{4} X_{1,a',r'}^{d',v',h',s',x',p'}(t)}{c_{2,a,r} \cdot \rho_{2,a,a',r,r'}(t) \cdot \sum_{d=1}^{8}\sum_{v=1}^{6}\sum_{h=1}^{7}\sum_{s=1}^{7}\sum_{x=1}^{4}\sum_{p=1}^{4} X_{2,a,r}^{d,v,h,s,x,p}(t)}$$

We then compute the adjusted contact rate for females as:

$$c^{*}_{2,a,a',r,r'}(t) = c_{2,a,r} \cdot \rho_{2,a,a',r,r'}(t) \cdot B_{a,a',r,r'}(t)^{\theta} \cdot \left(\frac{\sum_{d'=1}^{8}\sum_{v'=1}^{6}\sum_{h'=1}^{7}\sum_{s'=1}^{7}\sum_{x'=1}^{4}\sum_{p'=1}^{4} X_{1,a',r'}^{d',v',h',s',x',p'}(t)}{\sum_{d=1}^{8}\sum_{v=1}^{6}\sum_{h=1}^{7}\sum_{s=1}^{7}\sum_{x=1}^{4}\sum_{p=1}^{4} X_{2,a,r}^{d,v,h,s,x,p}(t)} \right)^{-(1-\theta)}$$

and for males, an adjusted contact rate of:

$$c^{*}_{1,a,a',r,r'}(t) = c_{1,a,r} \cdot \rho_{1,a,a',r,r'}(t) \cdot B_{a,a',r,r'}(t)^{-(1-\theta)} \cdot \left(\frac{\sum_{d'=1}^{8}\sum_{v'=1}^{6}\sum_{h'=1}^{7}\sum_{s'=1}^{7}\sum_{x'=1}^{4}\sum_{p'=1}^{4} X_{1,a',r'}^{d',v',h',s',x',p'}(t)}{\sum_{d=1}^{8}\sum_{v=1}^{6}\sum_{h=1}^{7}\sum_{s=1}^{7}\sum_{x=1}^{4}\sum_{p=1}^{4} X_{2,a,r}^{d,v,h,s,x,p}(t)} \right)^{\theta}$$

c. Transmission Probabilities

Per-partnership probability of transmission

Equation variables	
$A_{g,a,r}$	Number of acts per partnership of gender *g,* age *a,* and sexual-risk group *r*.<br>We assume zero acts for individuals below the age of sexual debut (age 10).
$\chi\_HIV_g^{v',x'}$	Per-act probability of HIV transmission to a person of gender *g* based on the viral load *v'* of the HIV-positive partner.<br>We assume the probability of female-to-male HIV transmission is equal to the probability of male-to-female transmission across all viral load stages ($\chi\_HIV_1^{v'} = \chi\_HIV_2^{v'}$). We reduce HIV per-act transmission as a proxy for decreased sexual activity during late-stage HIV (*v'* = 5), regional or distant cervical cancer (*x'* = 2 or *x'* = 3), or hysterectomy (*x'* = 4).
$\chi\_HPV_g^{v',x'}$	Per-act probability of HPV transmission to a person of gender *g*.

	We assume the per-act probability of HPV transmission is the same for vaccine-type and non-vaccine-type HPV and across all stages of pre-cancer or cervical cancer. We reduce HPV per-act transmission as a proxy for decreased sexual activity during late-stage HIV (v' = 5) or regional or distant cervical cancer (x' = 2 or x' = 3). We assume no HPV transmission after hysterectomy (x' = 4).

The per-partnership probability of HIV transmission $\beta\_HIV_{g,a,r}^{v',x'}$ is the cumulative risk of acquiring HIV from all sexual acts with a partner. This quantity depends on the per-act probability of HIV transmission and the number of acts per partnership.

We calculate the per-partnership probability of HIV transmission to a male partner:

$$\beta\_HIV_{1,a,r}^{v',x'} = 1 - (1 - \chi\_HIV_1^{v',x'})^{A_{1,a,r}}$$

Similarly, the per-partnership probability of HIV transmission to a female partner:

$$\beta\_HIV_{2,a,r}^{v',x'} = 1 - (1 - \chi\_HIV_2^{v',x'})^{A_{2,a,r}}$$

Likewise, the per-partnership probability of HPV transmission $\beta\_HPV_{g,a,r}^{v',x'}$ depends on the per-act probability of HPV transmission and the number of acts per partnership.

We calculate the per-partnership probability of HPV transmission to a male partner:

$$\beta\_HPV_{1,a,r}^{v',x'} = 1 - (1 - \chi\_HPV_1^{v',x'})^{A_{1,a,r}}$$

Similarly, the per-partnership probability of HPV transmission to a female partner:

$$\beta\_HPV_{2,a,r}^{v',x'} = 1 - (1 - \chi\_HPV_2^{v',x'})^{A_{2,a,r}}$$

Force of infection

Equation variables	
$c^*_{g,a,a',r,r'}(t)$	Adjusted yearly contact rate for persons of gender *g,* age *a,* and risk group *r,* with persons of the opposite gender, age *a',* and risk group *r'*.
$\beta\_HIV_{g,a,r}^{v',x'}$	Annual per-partnership probability of HIV transmission from a HIV-positive person with viral load v' and cervical cancer stage x' to a HIV-susceptible partner with gender *g,* age *a,* and risk group *r*.
$\beta\_HPV_{g,a,r}^{v',x'}$	Annual per-partnership probability of HPV transmission from a HPV-infected person with viral load v' and cervical cancer stage x' to a HPV-susceptible partner with gender *g,* age *a,* and risk group *r*.

The force of infection represents the cumulative risk of acquiring HIV or HPV from all possible partners, and depends on the adjusted contact rate, the per-partnership probability of transmission, and the proportion of sexually active persons who are HIV- or HPV-infected.

The force of infection $\lambda\_HIV_{g,a,r}(t)$ determines HIV disease transmission:

$$\lambda\_HIV_{g,a,r}(t) = \sum_{a'=1}^{16}\sum_{r'=1}^{3}\left(c^*_{g,a,a',r,r'}(t) \cdot \frac{-\sum_{v'=1}^{6}\sum_{x'=1}^{4}\ln\left(1-\beta\_HIV_{g,a,r}^{v',x'}\right)\cdot\sum_{d'=3}^{8}\sum_{h'=1}^{7}\sum_{s'=1}^{7}\sum_{p'=1}^{4}X_{g',a',r'}^{d',v',h',s',x',p'}(t)}{\sum_{d'=1}^{8}\sum_{v'=1}^{6}\sum_{h'=1}^{7}\sum_{s'=1}^{7}\sum_{x'=1}^{4}\sum_{p'=1}^{4}X_{g',a',r'}^{d',v',h',s',x',p'}(t)}\right)$$

Similarly, the force of infection $\lambda\_vHPV_{g,a,r}(t)$ determines vaccine-type HPV transmission:

$$\lambda\_vHPV_{g,a,r}(t) = \sum_{a'=1}^{16}\sum_{r'=1}^{3}\left(c^*_{g,a,a',r,r'}(t) \cdot \frac{-\sum_{v'=1}^{6}\sum_{x'=1}^{4}\ln\left(1-\beta\_HPV_{g,a,r}^{v',x'}\right)\cdot\sum_{d'=1}^{8}\sum_{h'=2}^{6}\sum_{s'=1}^{7}\sum_{p'=1}^{4}X_{g',a',r'}^{d',v',h',s',x',p'}(t)}{\sum_{d'=1}^{8}\sum_{v'=1}^{6}\sum_{h'=1}^{7}\sum_{s'=1}^{7}\sum_{x'=1}^{4}\sum_{p'=1}^{4}X_{g',a',r'}^{d',v',h',s',x',p'}(t)}\right)$$

and $\lambda\_nvHPV_{g,a,r}(t)$ defines non-vaccine-type HPV transmission:

$$\lambda\_nvHPV_{g,a,r}(t) = \sum_{a'=1}^{16}\sum_{r'=1}^{3}\left(c^{*}_{g,a,a',r,r'}(t) \cdot \frac{-\sum_{v'=1}^{6}\sum_{x'=1}^{4}\ln\left(1-\beta\_HPV_{g,a,r}^{v',x'}\right)\cdot\sum_{d'=1}^{8}\sum_{h'=1}^{7}\sum_{s'=2}^{6}\sum_{p'=1}^{4}X_{g',a',r'}^{d',v',h',s',x',p'}(t)}{\sum_{d'=1}^{8}\sum_{v'=1}^{6}\sum_{h'=1}^{7}\sum_{s'=1}^{7}\sum_{x'=1}^{4}\sum_{p'=1}^{4}X_{g',a',r'}^{d',v',h',s',x',p'}(t)}\right)$$

d. Natural History and Interventions

HIV

Equation variables	
$\mu\_HIV_{g,a}^{d}$	Annual HIV-associated mortality rate by gender *g*, age *a*, and HIV disease stage *d* for ($3 \le d \le 8$).
$\lambda\_HIV_{g,a,r}(t)$	Force of HIV infection fo persons without HIV by gender *g*, age *a*, and risk *r*.
ρ_HIV_g	Reduction in HIV acquisition due to circumcision by gender.<br>Only males receive circumcision ($\rho\_HIV_2 = 1$).
ψ_HIV_g	Reduction in HIV acquisition due to population-level condom use by gender.
ω^d	The rate of progressing from HIV stage *d* to stage *d + 1,* for ($3 \le d \le 7$).
l^d	The rate of progressing from viral load stage *v* to *v + 1,* for ($1 \le v \le 5$).
$P_{g,a}(t)$	The proportion of HIV-negative persons of gender *g* and age *a* that are circumcised.<br>Only males receive circumcision ($P_{2,a}(t) = 0$).
$A_{g,a}^{d}(t)$	The proportion of persons living with HIV of disease stage *d,* gender *g,* and age *a* that initiate ART.
$\sigma_{g,a,r}^{d,v}(t)$	The proportion of persons who discontinue ART based on the recent distribution of persons initiating ART by gender *g*, age *a,* risk *r,* disease *d,* and viral *v*.

We calculate changes in HIV status and HIV stage defined by CD4 count, viral load, and treatment status. The population without HIV can acquire HIV after sexual debut with a force of infection reduced by circumcision in males and condom use by either gender. We only track circumcision among men without HIV. Individuals with HIV infection experience HIV-associated mortality, CD4 and viral load stage progression, and ART initiation and discontinuation. CD4 and viral load stage are not tracked among persons on treatment.

HIV-negative, uncircumcised

$$\frac{dX_{g,a,r}^{1,1,h,s,x,p}(t)}{dt} = -\left(\psi\_HIV_g \cdot \lambda\_HIV_{g,a,r}(t) + P_{g,a}(t)\right) X_{g,a,r}^{1,1,h,s,x,p}(t)$$

HIV-negative, circumcised

$$\frac{dX_{g,a,r}^{2,1,h,s,x,p}(t)}{dt} = P_{g,a}(t) \cdot X_{g,a,r}^{1,1,h,s,x,p}(t) - \left(\psi\_HIV_g \cdot \rho\_HIV_g \cdot \lambda\_HIV_{g,a,r}(t)\right) X_{g,a,r}^{2,1,h,s,x,p}(t)$$

HIV-positive, acute infection

$$\frac{dX_{g,a,r}^{3,1,h,s,x,p}(t)}{dt} = \psi\_HIV_g \cdot \lambda\_HIV_{g,a,r}(t) \cdot X_{g,a,r}^{1,1,h,s,x,p}(t) + \psi\_HIV_g \cdot \rho\_HIV_g \cdot \lambda\_HIV_{g,a,r}(t) \cdot X_{g,a,r}^{2,1,h,s,x,p}(t) + \sigma_{g,a,r}^{3,1}(t) \cdot X_{g,a,r}^{8,6,h,s,x,p}(t) - \left(\mu\_HIV_{g,a}^{3} + \omega^3 + A_{g,a}^{3}(t)\right) X_{g,a,r}^{3,1,h,s,x,p}(t)$$

HIV-positive, CD4 > 500 cells/μL

$$\frac{dX_{g,a,r}^{4,v,h,s,x,p}(t)}{dt} = \omega^3 \cdot X_{g,a,r}^{3,v,h,s,x,p}(t) + l^{v-1} \cdot X_{g,a,r}^{4,v-1,h,s,x,p}(t) + \sigma_{g,a,r}^{4,v}(t) \cdot X_{g,a,r}^{8,6,h,s,x,p}(t) - \left(\mu\_HIV_{g,a}^{4} + \omega^4 + l^v + A_{g,a}^{4}(t)\right) X_{g,a,r}^{4,v,h,s,x,p}(t)$$

HIV-positive, CD4 350-500 cells/μL

$$\frac{dX_{g,a,r}^{5,v,h,s,x,p}(t)}{dt} = \omega^4 \cdot X_{g,a,r}^{4,v,h,s,x,p}(t) + l^{v-1} \cdot X_{g,a,r}^{5,v-1,h,s,x,p}(t) + \sigma_{g,a,r}^{5,v}(t) \cdot X_{g,a,r}^{8,6,h,s,x,p}(t) - \left(\mu\_HIV_{g,a}^{5} + \omega^5 + l^v + A_{g,a}^{5}(t)\right) X_{g,a,r}^{5,v,h,s,x,p}(t)$$

HIV-positive, CD4 200-350 cells/μL

$$\frac{dX_{g,a,r}^{6,v,h,s,x,p}(t)}{dt} = \omega^5 \cdot X_{g,a,r}^{5,v,h,s,x,p}(t) + l^{v-1} \cdot X_{g,a,r}^{6,v-1,h,s,x,p}(t) + \sigma_{g,a,r}^{6,v}(t) \cdot X_{g,a,r}^{8,6,h,s,x,p}(t) - \left(\mu\_HIV_{g,a}^{6} + \omega^6 + l^v + A_{g,a}^{6}(t)\right) X_{g,a,r}^{6,v,h,s,x,p}(t)$$

HIV-positive, CD4 ≤ 200 cells/μL

$$\frac{dX_{g,a,r}^{7,v,h,s,x,p}(t)}{dt} = \omega^6 \cdot X_{g,a,r}^{6,v,h,s,x,p}(t) + l^{v-1} \cdot X_{g,a,r}^{7,v-1,h,s,x,p}(t) + \sigma_{g,a,r}^{7,v}(t) \cdot X_{g,a,r}^{8,6,h,s,x,p}(t)$$
$$- \left(\mu\_HIV_{g,a}^7 + \omega^7 + l^v + A_{g,a}^7(t)\right) X_{g,a,r}^{7,v,h,s,x,p}(t)$$

HIV-positive, on ART

$$\frac{dX_{g,a,r}^{8,6,h,s,x,p}(t)}{dt} = \sum_{d=3}^{7}\sum_{v=1}^{5}\left(A_{g,a}^d(t) \cdot X_{g,a,r}^{d,v,h,s,x,p}(t) - \sigma_{g,a,r}^{d,v} \cdot X_{g,a,r}^{8,6,h,s,x,p}(t)\right)$$

HPV

Equation variables	
$\mu\_utHPV_g^{d,h,s,x}$	Annual untreated cervical cancer-associated mortality rate by gender *g*, HIV disease stage *d*, vaccine-type HPV stage *h*, non-vaccine-type HPV stage *s*, and cervical cancer stage *x* for ($1 \le x \le 6$). Only women have cervical cancer-associated mortality ($\mu\_HPV_1^{d,h,s,x} = 0$) and only when ($h = 6$ or $s = 6$).
$\mu\_tHPV_g^{d,h,s,x}$	Annual treated cervical cancer-associated mortality rate by gender *g*, HIV disease stage *d*, vaccine-type HPV stage *h*, non-vaccine-type HPV stage *s*, and cervical cancer stage *x* for ($7 \le x \le 9$). Only females have treated cervical cancer-associated mortality ($\mu\_tHPV_1^{d,h,s,x} = 0$) and only when ($h = 6$ or $s = 6$).
$\lambda\_vHPV_{g,a,r}(t)$	Force of vaccine-type HPV infection for susceptible persons of gender *g*, age *a*, and risk *r*.
$\lambda\_nvHPV_{g,a,r}(t)$	Force of non-vaccine-type HPV infection for susceptible persons of gender g, age *a*, and risk *r*.
$ж_d$	HPV acquisition risk multiplier for HIV-positive individuals with CD4 count ($4 \le d \le 7$).
ρ_HPV_g	HPV acquisition reduction multiplier due to circumcision by gender. Only males receive circumcision ($\rho\_HPV_2 = 1$).
ψ_HPV_g	HPV acquisition reduction multiplier due to population-level condom use by gender.
$\xi_{g,a}$	HPV acquisition reduction multiplier by gender and age for individuals with type-specific natural immunity. Only females temporarily develop partial natural immunity ($\xi_{1,a} = 0$). Older women develop stronger natural immunity than young girls.
ϕ_a	Vaccine-type HPV acquisition reduction multiplier by age for vaccinated individuals. We assume life-long protection with vaccination (ϕ_a is equivalent for all vaccinated ages).
$k\_v_{g,a}^{h,h'}$	Transition rate of progressing or regressing from vaccine-type HPV precancer or disease stage *h* to stage *h'*. Only women develop precancerous lesions and cervical cancer ($k\_v_{1,a}^{h,h'} = 0$ except for HPV clearance when $h = 2$ and $h' = 1$).
$k\_nv_{g,a}^{s,s'}$	Transition rate of progressing or regressing from non-vaccine-type HPV precancer or disease stage *s* to stage *s'*. Only women develop precancerous lesions and cervical cancer ($k\_v_{1,a}^{s,s'} = 0$ except for HPV clearance when $s = 2$ and $s' = 1$).
r_g	Rate of waning type-specific natural immunity. Only females temporarily develop partial natural immunity ($r_1 = 0$).
$\varphi_g^{h,s,x,x'}$	Progression rate of cervical cancer from stage *x* to stage *x'*. Only women develop cervical cancer ($\varphi_1^{h,s,x,x'} = 0$) and ($\varphi_2^{h,s,x,x'} > 0$ only when *h* or $s = 6$). Only untreated cervical cancers progress from stage *x* to stage *x'*.
$\zeta\_v^{d,h,h'}$	Transition rate multiplier for HIV-positive individuals progressing or regressing from vaccine-type precancer or disease stage *h* to stage *h'* with CD4 count *d*. Transition rate multipliers for HIV-positive individuals are the same for vaccine-type and non-vaccine-type HPV ($\zeta\_v^{d,h,h'} = \zeta\_nv^{d,s,s'}$ when $h = s$ and $h' = s'$).
$\zeta\_nv^{d,s,s'}$	Transition rate multiplier for HIV-positive individuals progressing or regressing from non-vaccine-type precancer or disease stage *s* to stage *s'* with gender *g* and CD4 count *d*. Transition rate multipliers for HIV-positive individuals are the same for vaccine-type and non-vaccine-type HPV ($\zeta\_v^{d,h,h'} = \zeta\_nv^{d,s,s'}$ when $h = s$ and $h' = s'$).
ℓ_g	Additional multiplier for clearance of vaccine or non-vaccine-type HPV infection. Only applied to males ($\ell_2 = 1$).
$V_{g,a}^d$	The proportion of persons with HIV disease status *d*, gender *g*, and age *a* vaccinated.
$k\_sy_g^x$	Annual probability of being diagnosed with cervical cancer due to symptoms by cervical cancer stage *x* ($1 \le x \le 3$).
ρ_tx_g	The proportion of women who are diagnosed with cervical cancer and continue to treatment.
$\rho\_hy_g^x$	The proportion of women who are diagnosed and treated with cervical cancer who are treated with hysterectomy, by cancer stage *x* ($1 \le x \le 3$).

Vaccine-targeted HPV types and precancer equations

Male susceptible

$$\frac{dX_{1,a,r}^{d,v,1,s,1,1}(t)}{dt} = \ell_1 \cdot \zeta\_v^{d,2,1} \cdot k\_v_{1,a}^{2,1} \cdot X_{1,a,r}^{d,v,2,s,1,1}(t)$$
$$- \left(ж_d \cdot \rho\_HPV_1 \cdot \psi\_HPV_1 \cdot \lambda\_vHPV_{1,a,r}(t) + V_{1,a}^d\right) X_{1,a,r}^{d,v,1,s,1,1}(t)$$

Male HPV-infected

$$\frac{dX_{1,a,r}^{d,v,2,s,1,1}(t)}{dt} = ж_d \cdot \rho_{HPV_1} \cdot \psi_{HPV_1} \cdot \lambda_{vHPV_{1,a,r}}(t) \cdot X_{1,a,r}^{d,v,1,s,1,1}(t) - \ell_1 \cdot \zeta\_v^{d,2,1} \cdot k\_v_{1,a}^{2,1} \cdot X_{1,a,r}^{d,v,2,s,1,1}(t)$$

Male susceptible, vaccinated

$$\frac{dX_{1,a,r}^{d,v,1,s,1,2}(t)}{dt} = V_{1,a}^{d} \cdot X_{1,a,r}^{d,v,1,s,1,1}(t) + \ell_1 \cdot \zeta\_v^{d,2,1} \cdot k\_v_{1,a}^{2,1} \cdot X_{1,a,r}^{d,v,2,s,1,2}(t) - \phi_a \cdot ж_d \cdot \rho\_HPV_1 \cdot \psi\_HPV_1 \cdot \lambda\_vHPV_{1,a,r}(t) \cdot X_{1,a,r}^{d,v,1,s,1,2}(t)$$

Male HPV-infected, vaccinated

$$\frac{dX_{1,a,r}^{d,v,2,s,1,2}(t)}{dt} = \phi_a \cdot ж_d \cdot \rho_{HPV_1} \cdot \psi_{HPV_1} \cdot \lambda_{vHPV_{1,a,r}}(t) \cdot X_{1,a,r}^{d,v,1,s,1,2}(t) - \ell_1 \cdot \zeta\_v^{d,2,1} \cdot k\_v_{1,a}^{2,1} \cdot X_{1,a,r}^{d,v,2,s,1,2}(t)$$

Female, susceptible

Untreated cervical cancer health states

$$\frac{dX_{2,a,r}^{d,v,1,s,[1,2,3,4,5,6],[1,3]}(t)}{dt} = \zeta\_v^{d,7,1} \cdot r_2 \cdot X_{2,a,r}^{d,v,7,s,[1,2,3,4,5,6],[1,3]}(t) - \left(ж_d \cdot \psi\_HPV_2 \cdot \lambda\_vHPV_{2,a,r}(t) + V_{2,a}^{d} + \mu\_utHPV_2^{d,1,s,[1,2,3,4,5,6]}\right) X_{2,a,r}^{d,v,1,s,[1,2,3,4,5,6],[1,3]}(t)$$

Treated cervical cancer health states

$$\frac{dX_{2,a,r}^{d,v,1,s,[7,8,9],[1,3]}(t)}{dt} = \zeta\_v^{d,7,1} \cdot r_2 \cdot X_{2,a,r}^{d,v,7,s,[7,8,9],[1,3]}(t) - \left(ж_d \cdot \psi\_HPV_2 \cdot \lambda\_vHPV_{2,a,r}(t) + V_{2,a}^{d} + \mu\_tHPV_2^{d,1,s,[7,8,9]}\right) X_{2,a,r}^{d,v,1,s,[7,8,9],[1,3]}(t)$$

Female, immune

Untreated cervical cancer health states

$$\frac{dX_{2,a,r}^{d,v,7,s,[1,2,3,4,5,6],[1,3]}(t)}{dt} = V_{2,a}^{d} \cdot X_{2,a,r}^{d,v,7,s,[1,2,3,4,5,6],[1,3]}(t) + \zeta\_v^{d,2,7} \cdot k\_v_{2,a}^{2,7} \cdot X_{2,a,r}^{d,v,2,s,[1,2,3,4,5,6],[1,3]}(t) - \left(\zeta\_v^{d,7,1} \cdot k\_v_{2,a}^{7,1} + \xi_{2,a} \cdot ж_d \cdot \psi\_HPV_2 \cdot \lambda\_vHPV_{2,a,r}(t) + V_{2,a}^{d} + \mu\_utHPV_2^{d,7,s,[1,2,3,4,5,6]}\right) X_{2,a,r}^{d,v,7,s,[1,2,3,4,5,6],[1,3]}(t)$$

Treated cervical cancer health states

$$\frac{dX_{2,a,r}^{d,v,7,s,[7,8,9],[1,3]}(t)}{dt} = V_{2,a}^{d} \cdot X_{2,a,r}^{d,v,7,s,[7,8,9],[1,3]}(t) + \zeta\_v^{d,2,7} \cdot k\_v_{2,a}^{2,7} \cdot X_{2,a,r}^{d,v,2,s,[7,8,9],[1,3]}(t) - \left(\zeta\_v^{d,7,1} \cdot k\_v_{2,a}^{7,1} + \xi_{2,a} \cdot ж_d \cdot \psi\_HPV_2 \cdot \lambda\_vHPV_{2,a,r}(t) + V_{2,a}^{d} + \mu\_tHPV_2^{d,7,s,[7,8,9]}\right) X_{2,a,r}^{d,v,7,s,[7,8,9],[1,3]}(t)$$

Female, HPV-infected

Untreated cervical cancer health states

$$\frac{dX_{2,a,r}^{d,v,2,s,[1,2,3,4,5,6],[1,3]}(t)}{dt} = \zeta\_v^{d,3,2} \cdot k\_v_{2,a}^{3,2} \cdot X_{2,a,r}^{d,v,3,s,[1,2,3,4,5,6],[1,3]}(t) + ж_d \cdot \psi\_HPV_2 \cdot \lambda\_vHPV_{2,a,r}(t) \cdot X_{2,a,r}^{d,v,1,s,[1,2,3,4,5,6],[1,3]}(t) + \xi_{2,a} \cdot ж_d \cdot \psi\_HPV_2 \cdot \lambda\_vHPV_{2,a,r}(t) \cdot X_{2,a,r}^{d,v,7,s,[1,2,3,4,5,6],[1,3]}(t) - \left(\zeta\_v^{d,2,7} \cdot k\_v_{2,a}^{2,7} + \zeta\_v^{d,2,3} \cdot k\_v_{2,a}^{2,3} + \mu\_utHPV_2^{d,2,s,[1,2,3,4,5,6]}\right) X_{2,a,r}^{d,v,2,s,[1,2,3,4,5,6],[1,3]}(t)$$

Treated cervical cancer health states

$$\frac{dX_{2,a,r}^{d,v,2,s,[7,8,9],[1,3]}(t)}{dt} = \zeta\_v^{d,3,2} \cdot k\_v_{2,a}^{3,2} \cdot X_{2,a,r}^{d,v,3,s,[7,8,9],[1,3]}(t) + ж_d \cdot \psi\_HPV_2 \cdot \lambda\_vHPV_{2,a,r}(t) \cdot X_{2,a,r}^{d,v,1,s,[7,8,9],[1,3]}(t) + \xi_{2,a} \cdot ж_d \cdot \psi\_HPV_2 \cdot \lambda\_vHPV_{2,a,r}(t) \cdot X_{2,a,r}^{d,v,7,s,[7,8,9],[1,3]}(t) - \left(\zeta\_v^{d,2,7} \cdot k\_v_{2,a}^{2,7} + \zeta\_v^{d,2,3} \cdot k\_v_{2,a}^{2,3} + \mu\_tHPV_2^{d,2,s,[7,8,9]}\right) X_{2,a,r}^{d,v,2,s,[7,8,9],[1,3]}(t)$$

Female, susceptible, vaccinated

Untreated cervical cancer health states

$$\frac{dX_{2,a,r}^{d,v,1,s,[1,2,3,4,5,6],[2,4]}(t)}{dt} = \zeta\_v^{d,7,1} \cdot r_2 \cdot X_{2,a,r}^{d,v,7,s,[1,2,3,4,5,6],[2,4]}(t) + V_{2,a}^{d} \cdot X_{2,a,r}^{d,v,1,s,[1,2,3,4,5,6],[1,3]}(t) - \left(\phi_a \cdot ж_d \cdot \psi\_HPV_2 \cdot \lambda\_vHPV_{2,a,r}(t) + \mu\_utHPV_2^{d,1,s,[1,2,3,4,5,6]}\right) X_{2,a,r}^{d,v,1,s,[1,2,3,4,5,6],[2,4]}(t)$$

Treated cervical cancer health states

$$\frac{dX_{2,a,r}^{d,v,1,s,[7,8,9],[2,4]}(t)}{dt} = \zeta\_v^{d,7,1} \cdot r_2 \cdot X_{2,a,r}^{d,v,7,s,[7,8,9],[2,4]}(t) + V_{2,a}^{d} \cdot X_{2,a,r}^{d,v,1,s,[7,8,9],[1,3]}(t) - \left(\phi_a \cdot ж_d \cdot \psi\_HPV_2 \cdot \lambda\_vHPV_{2,a,r}(t) + \mu\_tHPV_2^{d,1,s,[7,8,9]}\right) X_{2,a,r}^{d,v,1,s,[7,8,9],[2,4]}(t)$$

Female, immune, vaccinated

Untreated cervical cancer health states

$$\frac{dX_{2,a,r}^{d,v,7,s,[1,2,3,4,5,6],[2,4]}(t)}{dt} = \zeta\_v^{d,2,7} \cdot k\_v_{2,a}^{2,7} \cdot X_{2,a,r}^{d,v,2,s,[1,2,3,4,5,6],[2,4]}(t) + V_{2,a}^{d} \cdot X_{2,a,r}^{d,v,7,s,[1,2,3,4,5,6],[1,3]}(t) - \left(\zeta\_v^{d,7,1} \cdot k\_v_{2,a}^{7,1} + \phi_a \cdot \xi_{2,a} \cdot ж_d \cdot \psi\_HPV_2 \cdot \lambda\_vHPV_{2,a,r}(t) + \mu\_utHPV_2^{d,7,s,[1,2,3,4,5,6]}\right) X_{2,a,r}^{d,v,7,s,[1,2,3,4,5,6],[2,4]}(t)$$

Treated cervical cancer health states

$$\frac{dX_{2,a,r}^{d,v,7,s,[7,8,9],[2,4]}(t)}{dt} = \zeta\_v^{d,2,7} \cdot k\_v_{2,a}^{2,7} \cdot X_{2,a,r}^{d,v,2,s,[7,8,9],[2,4]}(t) + V_{2,a}^{d} \cdot X_{2,a,r}^{d,v,7,s,[7,8,9],[1,3]}(t) - \left(\zeta\_v^{d,7,1} \cdot k\_v_{2,a}^{7,1} + \phi_a \cdot \xi_{2,a} \cdot ж_d \cdot \psi\_HPV_2 \cdot \lambda\_vHPV_{2,a,r}(t) + \mu\_tHPV_2^{d,7,s,[7,8,9]}\right) X_{2,a,r}^{d,v,7,s,[7,8,9],[2,4]}(t)$$

Female, HPV-infected, vaccinated

Untreated cervical cancer health states

$$\frac{dX_{2,a,r}^{d,v,2,s,[1,2,3,4,5,6],[2,4]}(t)}{dt} = \zeta\_v^{d,3,2} \cdot k\_v_{2,a}^{3,2} \cdot X_{2,a,r}^{d,v,3,s,[1,2,3,4,5,6],[2,4]}(t) + \phi_a \cdot ж_d \cdot \psi\_HPV_2 \cdot \lambda\_vHPV_{2,a,r}(t) \cdot X_{2,a,r}^{d,v,2,s,[1,2,3,4,5,6],[2,4]}(t) + \phi_a \cdot \xi_{2,a} \cdot ж_d \cdot \psi\_HPV_2 \cdot \lambda\_vHPV_{2,a,r}(t) \cdot X_{2,a,r}^{d,v,7,s,[1,2,3,4,5,6],[2,4]}(t) - \left(\zeta\_v^{d,2,7} \cdot k\_v_{2,a}^{2,7} + \zeta\_v^{d,2,3} \cdot k\_v_{2,a}^{2,3} + \mu\_utHPV_2^{d,2,s,[1,2,3,4,5,6]}\right) X_{2,a,r}^{d,v,2,s,[1,2,3,4,5,6],[2,4]}(t)$$

Treated cervical cancer health states

$$\frac{dX_{2,a,r}^{d,v,2,s,[7,8,9],[2,4]}(t)}{dt} = \zeta\_v^{d,3,2} \cdot k\_v_{2,a}^{3,2} \cdot X_{2,a,r}^{d,v,3,s,[7,8,9],[2,4]}(t) + \phi_a \cdot ж_d \cdot \psi\_HPV_2 \cdot \lambda\_vHPV_{2,a,r}(t) \cdot X_{2,a,r}^{d,v,2,s,[7,8,9],[2,4]}(t) + \phi_a \cdot \xi_{2,a} \cdot ж_d \cdot \psi\_HPV_2 \cdot \lambda\_vHPV_{2,a,r}(t) \cdot X_{2,a,r}^{d,v,7,s,[7,8,9],[2,4]}(t) - \left(\zeta\_v^{d,2,7} \cdot k\_v_{2,a}^{2,7} + \zeta\_v^{d,2,3} \cdot k\_v_{2,a}^{2,3} + \mu\_tHPV_2^{d,2,s,[7,8,9]}\right) X_{2,a,r}^{d,v,2,s,[7,8,9],[2,4]}(t)$$

Female, CIN1

Untreated cervical cancer health states

$$\frac{dX_{2,a,r}^{d,v,3,s,[1,2,3,4,5,6],p}(t)}{dt} = \zeta\_v^{d,4,3} \cdot k\_v_{2,a}^{4,3} \cdot X_{2,a,r}^{d,v,4,s,[1,2,3,4,5,6],p}(t) + \zeta\_v^{d,2,3} \cdot k\_v_{2,a}^{2,3} \cdot X_{2,a,r}^{d,v,2,s,[1,2,3,4,5,6],p}(t) - \left(\zeta\_v^{d,3,4} \cdot k\_v_{2,a}^{3,4} + \zeta\_v^{d,3,2} \cdot k\_v_{2,a}^{3,2} + \mu\_utHPV_2^{d,3,s,[1,2,3,4,5,6]}\right) X_{2,a,r}^{d,v,3,s,[1,2,3,4,5,6],p}(t)$$

Treated cervical cancer health states

$$\frac{dX_{2,a,r}^{d,v,3,s,[7,8,9],p}(t)}{dt} = \zeta\_v^{d,4,3} \cdot k\_v_{2,a}^{4,3} \cdot X_{2,a,r}^{d,v,4,s,[7,8,9],p}(t) + \zeta\_v^{d,2,3} \cdot k\_v_{2,a}^{2,3} \cdot X_{2,a,r}^{d,v,2,s,[7,8,9],p}(t) - \left(\zeta\_v^{d,3,4} \cdot k\_v_{2,a}^{3,4} + \zeta\_v^{d,3,2} \cdot k\_v_{2,a}^{3,2} + \mu\_tHPV_2^{d,3,s,[7,8,9]}\right) X_{2,a,r}^{d,v,3,s,[7,8,9],p}(t)$$

Female, CIN2

Untreated cervical cancer health states

$$\frac{dX_{2,a,r}^{d,v,4,s,[1,2,3,4,5,6],p}(t)}{dt} = \zeta\_v^{d,5,4} \cdot k\_v_{2,a}^{5,4} \cdot X_{2,a,r}^{d,v,5,s,[1,2,3,4,5,6],p}(t) + \zeta\_v^{d,3,4} \cdot k\_v_{2,a}^{3,4} \cdot X_{2,a,r}^{d,v,3,s,[1,2,3,4,5,6],p}(t) - \left(\zeta\_v^{d,4,5} \cdot k\_v_{2,a}^{4,5} + \zeta\_v^{d,4,3} \cdot k\_v_{2,a}^{4,3} + \mu\_utHPV_2^{d,4,s,[1,2,3,4,5,6]}\right) X_{2,a,r}^{d,v,4,s,[1,2,3,4,5,6],p}(t)$$

Treated cervical cancer health states

$$\frac{dX_{2,a,r}^{d,v,4,s,[7,8,9],p}(t)}{dt} = \zeta\_v^{d,5,4} \cdot k\_v_{2,a}^{5,4} \cdot X_{2,a,r}^{d,v,5,s,[7,8,9],p}(t) + \zeta\_v^{d,3,4} \cdot k\_v_{2,a}^{3,4} \cdot X_{2,a,r}^{d,v,3,s,[7,8,9],p}(t) - \left(\zeta\_v^{d,4,5} \cdot k\_v_{2,a}^{4,5} + \zeta\_v^{d,4,3} \cdot k\_v_{2,a}^{4,3} + \mu\_tHPV_2^{d,4,s,[7,8,9]}\right) X_{2,a,r}^{d,v,4,s,[7,8,9],p}(t)$$

Female, CIN3

Untreated cervical cancer health states

$$\frac{dX_{2,a,r}^{d,v,5,s,[1,2,3,4,5,6],p}(t)}{dt} = \zeta\_v^{d,4,5} \cdot k\_v_{2,a}^{4,5} \cdot X_{2,a,r}^{d,v,4,s,[1,2,3,4,5,6],p}(t) - \left(\zeta\_v^{d,5,6} \cdot k\_v_{2,a}^{5,6} + \zeta\_v^{d,5,4} \cdot k\_v_{2,a}^{5,4} + \mu\_utHPV_2^{d,5,s,[1,2,3,4,5,6]}\right) X_{2,a,r}^{d,v,5,s,[1,2,3,4,5,6],p}(t)$$

Treated cervical cancer health states

$$\frac{dX_{2,a,r}^{d,v,5,s,[7,8,9],p}(t)}{dt} = \zeta\_v^{d,4,5} \cdot k\_v_{2,a}^{4,5} \cdot X_{2,a,r}^{d,v,4,s,[7,8,9],p}(t) - \left(\zeta\_v^{d,5,6} \cdot k\_v_{2,a}^{5,6} + \zeta\_v^{d,5,4} \cdot k\_v_{2,a}^{5,4} + \mu\_tHPV_2^{d,5,s,[7,8,9]}\right) X_{2,a,r}^{d,v,5,s,[7,8,9],p}(t)$$

Non-vaccine-targeted HPV types and precancer equations

The non-vaccine-type HPV and precancer equations follow the same pattern as the vaccine-type HPV equations with a few updates. All values of s equal the values of h in the vaccine-type equations, and h equals any value. Vaccination does not depend on non-vaccine-type HPV infection status.

Cervical cancer equations

Female cervical cancer, local

(where h=6)

$$\frac{dX_{2,a,r}^{d,v,6,s,x,p}(t)}{dt} = \zeta\_v^{d,5,6} \cdot k\_v_{2,a}^{5,6} \cdot X_{2,a,r}^{d,v,5,s,x,p}(t)$$

(where s=6)

$$\frac{dX_{2,a,r}^{d,v,h,6,x,p}(t)}{dt} = \zeta\_v^{d,5,6} \cdot k\_v_{2,a}^{5,6} \cdot X_{2,a,r}^{d,v,h,6,x,p}(t)$$

(where h=6 or s=6, and x=1 or x=4)

$$\frac{dX_{2,a,r}^{d,v,h,s,[1,4],p}(t)}{dt} = -\left(\varphi_2^{h,s,[1,4],[2,5]} + \mu\_utHPV_2^{d,h,s,[1,4]}\right) X_{2,a,r}^{d,v,h,s,[1,4],p}(t)$$

(where h=6 or s=6, and x=7)

$$\frac{dX_{2,a,r}^{d,v,h,s,7,p}(t)}{dt} = -\left(\mu\_tHPV_2^{d,h,s,7}\right) X_{2,a,r}^{d,v,h,s,7,p}(t)$$

Female cervical cancer, regional

(where h=6 or s=6, and x=2 or x=5)

$$\frac{dX_{2,a,r}^{d,v,h,s,[2,5],p}(t)}{dt} = \varphi_2^{h,s,[1,4],[2,5]} \cdot X_{2,a,r}^{d,v,h,s,[1,4],p}(t) - \left(\varphi_2^{h,s,[2,5],[3,6]} + \mu\_utHPV_2^{d,h,s,[2,5]}\right) X_{2,a,r}^{d,v,h,s,[2,5],p}(t)$$

(where h=6 or s=6, and x=8)

$$\frac{dX_{2,a,r}^{d,v,h,s,8,p}(t)}{dt} = -\left(\mu\_tHPV_2^{d,h,s,8}\right) X_{2,a,r}^{d,v,h,s,8,p}(t)$$

Female cervical cancer, distant

(where h=6 or s=6, and x=3 or x=6)

$$\frac{dX_{2,a,r}^{d,v,h,s,[3,6],p}(t)}{dt} = \varphi_2^{h,s,[2,5],[3,6]} \cdot X_{2,a,r}^{d,v,h,s,[2,5],p}(t) - \left(\mu\_utHPV_2^{d,h,s,[3,6]}\right) X_{2,a,r}^{d,v,h,s,[3,6],p}(t)$$

(where h=6 or s=6, and x=9)

$$\frac{dX_{2,a,r}^{d,v,h,s,9,p}(t)}{dt} = -\left(\mu\_tHPV_2^{d,h,s,9}\right) X_{2,a,r}^{d,v,h,s,9,p}(t)$$

Rate of Symptomatic Detection of Cervical Cancer

Female cervical cancer, local, untreated

$$\frac{dX_{2,a,r}^{d,v,h,s,4,p}(t)}{dt} = (1-\rho_{tx_2}) \cdot k\_sy^1 \cdot X_{2,a,r}^{d,v,h,s,1,p}(t)$$

Female cervical cancer, local, treated by other modalities

$$\frac{dX_{2,a,r}^{d,v,h,s,7,p}(t)}{dt} = \left[\rho_{tx_2} \cdot (1-\rho_{hy_2^1})\right] \cdot k\_sy^1 \cdot X_{2,a,r}^{d,v,h,s,1,p}(t)$$

Female cervical cancer, regional, untreated

$$\frac{dX_{2,a,r}^{d,v,h,s,5,p}(t)}{dt} = (1-\rho_{tx_2}) \cdot k\_sy^2 \cdot X_{2,a,r}^{d,v,h,s,2,p}(t)$$

Female cervical cancer, regional, treated by other modalities

$$\frac{dX_{2,a,r}^{d,v,h,s,8,p}(t)}{dt} = \left[\rho_{tx_2} \cdot (1-\rho_{hy_2^2})\right] \cdot k\_sy^2 \cdot X_{2,a,r}^{d,v,h,s,2,p}(t)$$

Female cervical cancer, distant, untreated

$$\frac{dX_{2,a,r}^{d,v,h,s,6,p}(t)}{dt} = (1-\rho_{tx_2}) \cdot k\_sy^3 \cdot X_{2,a,r}^{d,v,h,s,3,p}(t)$$

Female cervical cancer, distant, treated by other modalities

$$\frac{dX_{2,a,r}^{d,v,h,s,9,p}(t)}{dt} = \left[\rho_{tx_2} \cdot (1-\rho_{hy_2^3})\right] \cdot k\_sy^3 \cdot X_{2,a,r}^{d,v,h,s,3,p}(t)$$

Female cervical cancer, treated by hysterectomy

$$\frac{dX_{2,a,r}^{d,v,h,s,10,p}(t)}{dt} = \left[\rho_{tx_2} \cdot \rho_{hy_2^1}\right] \cdot k\_sy^1 \cdot X_{2,a,r}^{d,v,h,s,1,p}(t) + \left[\rho_{tx_2} \cdot \rho_{hy_2^2}\right] \cdot k\_sy^2 \cdot X_{2,a,r}^{d,v,h,s,2,p}(t) + \left[\rho_{tx_2} \cdot \rho_{hy_2^3}\right] \cdot k\_sy^3 \cdot X_{2,a,r}^{d,v,h,s,3,p}(t)$$

VI. Reporting

Our paper conforms to published guidance for reporting modeling studies of HPV prevention and economic evaluations – HPV-FRAME[128] and Consolidated Health Economic Evaluation Reporting Standards (CHEERS) 2022[129], respectively.

a. HPV-FRAME Checklist

Inputs	Reported by age? (Y/N)	Reported by sex?	Comments
Target population for intervention	Y	Y	Vaccination of girls by the age of 10. Additional scenarios explore catch-up vaccination of females age 10-19 or 10-24, as well as vaccination for all by age 10.
Sexual behavior	Y	Y	Sexual risk distribution reported by age in Table S4.
Cohort examined for evaluation / time horizon	Y	Y	100-year time horizon from 2023-2123. Intervention is given to cohorts, but we examine the outcome in the population.
Quality of life assumptions	N	Y	Disability weights were used for cervical cancer health state. Disability weights were only applied to women, but not stratified by age (see Section Methods of the article).
Calibration	Y	Y	The model was calibrated with country-specific behavioral and epidemiological data (see Section IIIa of the Supplementary Materials).
Validation (where possible)	Y	Y	The model was validated to HIV, HPV, and cervical cancer outcomes (see Section IIIb of the Supplementary Materials).
Costs	N	Y	All costs were related to both cervical cancer (screening and treatment) and HPV vaccination, drawn from published literature and insights from in-country experts (see Section Methods of the article).
**Reporting standards for models of vaccination in adolescent individuals**			
Vaccine coverage	Y	Y	See Methods Section of the article for information on the vaccine coverages modelled.
Vaccine efficacy	Y	Y	See Methods Section of the article for the assumptions around one- and two-dose efficacy.
Vaccine cross-protection	N	N	We do not account for cross-protection against additional HPV types.
Duration vaccine protection and waning	Y	Y	Waning assumptions are described in the Methods Section of the article. We assume duration of protection is the same regardless of age or sex.
Vaccine and delivery costs	Y	Y	See Table 1 of the article for vaccine and delivery costs. We

			assume costs are the same across all ages and sexes.
Pre-vaccination disease burden	Y	Y	Pre-vaccination disease burden is reflected in the model validation, as shown in III of the Supplementary Materials.
Duration of natural immunity	Y	Y	See Section IIc of the Supplementary Materials.
**Reporting standards for evaluations assessing alternative vaccine types or reduced-dose schedules**			
Timing between doses	Not applicable	Not applicable	The model does not simulate timing between doses. The time at which an individual is fully vaccinated is when they receive the second dose.
**Reporting standards for models of HPV prevention in LMIC**			
HIV prevalence rates, if endemic in country	Y	Y	The model was calibrated to HIV prevalence data stratified by age and sex (see Section IIIa of the Supplementary Materials).
Description of any opportunistic or pilot/demonstration screening projects ongoing	Y	Y	We ran scenarios reflecting current screening and vaccination coverage in Kenya (see Section Methods of the article).
Costs	N	Y	Total costs of each strategy are reported in Table 3 of the Section Results of the article.
**Reporting standards for models of HPV-associated cancers among individuals living with HIV (ILWH)**			
HPV prevalence, CIN prevalence, and cervical cancer incidence by HIV status	N	N	The purpose of the study was to evaluate cervical cancer outcomes nationally in Kenya so we did not stratify results by HIV status at this time.
HPV disease multipliers on HPV acquisition, progression from HPV infection to cancer for HIV-infected women/men	Y	Y	HPV acquisition multipliers for persons living with HIV is reported in Table S9 of the Supplementary Materials.
HPV-associated cancer mortality by HIV status	Y	Y	Cervical cancer-associated mortality rates by HIV health state are reported in Table S10 of the Supplementary Materials.
Relevant co-morbidities	Y	Y	Mortality rates account for HIV-associated mortality, cervical cancer-associated mortality, and background mortality due to other causes (see Section II of the Supplementary Materials).
HPV-associated screening sensitivity/specificity by HIV status	N	Y	Screening test performance stratified by HIV status is reported in Section IIv of the Supplementary Materials. Test performance is assumed to be the same for all age groups.
**Reporting standards for models of cervical screening**			
Routine screening behavior	Y	Y	We model once-per lifetime cervical screening for women in the age range of 35-39 with VIA,

			colposcopy triage, and cryotherapy treatment (see Section IIv of the Supplementary Materials).
Screening test(s) and colposcopy accuracies	N	Y	Accuracy of VIA and colposcopy is reported in Section IIv of the Supplementary Materials. Accuracy is assumed to be the same for all age groups.
Abnormal test management	N	Y	Follow-up after an abnormal screening test is reported in Section IIv of the Supplementary Materials. Follow-up tests are not stratified by age.
Diagnostic follow-up of abnormal tests	N	Y	Follow-up after an abnormal screening test is reported in Section IIv of the Supplementary Materials. Follow-up tests are not stratified by age.
Management by disease grade	N	Y	Treatment is by cryotherapy. Management is consistent across all ages and disease grades. This is reported in Section IIv of the Supplementary Materials.
Sources of information for screening structure and parameterization	Y	Y	Screening structure in the model is described in Section IIv of the Supplementary Materials.
**Reporting standards for integrated models of cervical screening and vaccination**			
HPV type incidence, clearance, and progression rates	Y	Y	HPV natural history parameters stratified by age are reported in Table S9 of the Supplementary Materials.
Herd effect	Y	Y	The model is dynamic in natural history, so it captures population-level effects such as herd immunity.
Association between vaccination and screening uptake	Y	Y	The model assumes the same level of screening uptake by age regardless of vaccination status.
Screening test(s) and colposcopy accuracies	N	Y	Screening test performance and accuracy of colposcopy is reported in Section IIv of the Supplementary Materials. It is not stratified by age.
Fixed-variable costs	N	Y	All cost assumptions are reported in Table 1 of the article. Costs are not stratified by age.
**B. Outputs**	**Reported by age? (Y/N)**	**Reported by sex?**	**Comments**
**Core reporting standards**			
Cancer incidence, mortality, life years, QALYs/DALYs (as appropriate)	N	Y	Cancer incidence is reported in Table 2, mortality in Table S17 (in the Supplementary Materials), life years in Tables S19 and S20 (in the Supplementary Materials), and DALYs in Tables S19 and S20 (in the Supplementary Materials).

HPV prevalence, pre-intervention	N	Y	HPV outcomes pre-intervention were reported in the previous modeling publication by Liu, et al.[2]
CIN2/3 detected	N	Y	CIN outcomes were reported during model calibration, shown in Figure S7 of the Supplementary Materials.
Sensitivity analysis on key inputs	N	Y	Results of sensitivity analyses are described in Section IVdiii of the Supplementary Materials.
Incremental cost-effectiveness ratios and costs saved	N	Y	ICERs are reported in Table 3 of the article.
**Reporting standards for models of vaccination in adolescent individuals**			
Absolute reductions in HPV infections, and/or warts, post-vaccination	N	N	This was not presented since this study focuses on the impact of vaccination on the burden of cervical cancer.
Absolute reductions in CIN2+ post-vaccination	N	N	This was not presented since this study focuses on the impact of vaccination on the burden of cervical cancer.
Absolute reductions in invasive cancer (cervical and other HPV cancers, as relevant) post-vaccination	N	Y	We presented reduction in cervical cancer cases and incidence in Table 2 of the article. We do not present the results stratified by age.
**Reporting standards for models of HPV-associated cancers among individuals living with HIV (ILWH)**			
Reduction in cervical cancer incidence over time by HIV status (and CD4 count and ART status if modelled)	N	N	The purpose of this article was to explore the population-level health economic impact of HPV vaccination. Cervical cancer outcomes by HIV status are beyond the scope of this article.

b. CHEERS 2022 Checklist

Section/topic	Item No	Guidance for reporting	Reported in section
**Title**			
Title	1	Identify the study as an economic evaluation and specify the interventions being compared.	Introduction
**Abstract**			
Abstract	2	Provide a structured summary that highlights context, key methods, results, and alternative analyses.	Abstract
**Introduction**			
Background and objectives	3	Give the context for the study, the study question, and its practical relevance for decision making in policy or practice.	Introduction
**Methods**			

Section/topic	Item No	Guidance for reporting	Reported in section
Health economic analysis plan	4	Indicate whether a health economic analysis plan was developed and where available.	Methods
Study population	5	Describe characteristics of the study population (such as age range, demographics, socioeconomic, or clinical characteristics).	Methods
Setting and location	6	Provide relevant contextual information that may influence findings.	Introduction, Methods
Comparators	7	Describe the interventions or strategies being compared and why chosen.	Methods
Perspective	8	State the perspective(s) adopted by the study and why chosen.	Methods, Discussion
Time horizon	9	State the time horizon for the study and why appropriate.	Methods, Discussion
Discount rate	10	Report the discount rate(s) and reason chosen.	Methods
Selection of outcomes	11	Describe what outcomes were used as the measure(s) of benefit(s) and harm(s).	Methods
Measurement of outcomes	12	Describe how outcomes used to capture benefit(s) and harm(s) were measured.	Methods
Valuation of outcomes	13	Describe the population and methods used to measure and value outcomes.	Methods
Measurement and valuation of resources and costs	14	Describe how costs were valued.	Methods
Currency, price date, and conversion	15	Report the dates of the estimated resource quantities and unit costs, plus the currency and year of conversion.	Methods
Rationale and description of model	16	If modelling is used, describe in detail and why used. Report if the model is publicly available and where it can be accessed.	Methods
Analytics and assumptions	17	Describe any methods for analysing or statistically transforming data, any extrapolation methods, and approaches for validating any model used.	Methods, Supplementary Materials
Characterizing heterogeneity	18	Describe any methods used for estimating how the results of the study vary for subgroups.	Not applicable
Characterizing distributional effects	19	Describe how impacts are distributed across different individuals or adjustments made to reflect priority populations.	Not applicable
Characterizing uncertainty	20	Describe methods to characterise any sources of uncertainty in the analysis.	Methods
Approach to engagement with	21	Describe any approaches to engage patients or service recipients, the general public,	Methods

Section/topic	Item No	Guidance for reporting	Reported in section
patients and others affected by the study		communities, or stakeholders (such as clinicians or payers) in the design of the study.	
**Results**			
Study parameters	22	Report all analytic inputs (such as values, ranges, references) including uncertainty or distributional assumptions.	Results
Summary of main results	23	Report the mean values for the main categories of costs and outcomes of interest and summarise them in the most appropriate overall measure.	Results
Effect of uncertainty	24	Describe how uncertainty about analytic judgments, inputs, or projections affect findings. Report the effect of choice of discount rate and time horizon, if applicable.	Supplementary Materials
Effect of engagement with patients and others affected by the study	25	Report on any difference patient/service recipient, general public, community, or stakeholder involvement made to the approach or findings of the study	Not applicable
**Discussion**			
Study findings, limitations, generalizability, and current knowledge	26	Report key findings, limitations, ethical or equity considerations not captured, and how these could affect patients, policy, or practice.	Discussion
**Other relevant information**			
Source of funding	27	Describe how the study was funded and any role of the funder in the identification, design, conduct, and reporting of the analysis	Funding disclosure
Conflicts of interest	28	Report authors conflicts of interest according to journal or International Committee of Medical Journal Editors requirements.	Conflict of interest disclosure

VII. References

1. UNAIDS. UNAIDS Data Geneva, Switzerland: Joint United Nations Programme on HIV/AIDS, 2020.
2. Liu G, Mugo NR, Bayer C, et al. Impact of catch-up human papillomavirus vaccination on cervical cancer incidence in Kenya: A mathematical modeling evaluation of HPV vaccination strategies in the context of moderate HIV prevalence. *EClinicalMedicine* 2022; **45**: 101306.
3. United Nations Department of Economic and Social Affairs. World Population Prospects. 2019. https://population.un.org/wpp/Download/Standard/Population/ (accessed June 20 2020).
4. Kenya National Bureau of Statistics. The 2009 Kenya Population and Housing Census. Nairobi, Kenya: Kenya National Bureau of Statistics, 2010.
5. Kenya National Bureau of Statistics. The 2019 Kenya Population and Housing Census. Nairobi, Kenya: Kenya National Bureau of Statistics, 2020.

6. Ross A, Van der Paal L, Lubega R, Mayanja BN, Shafer LA, Whitworth J. HIV-1 disease progression and fertility: the incidence of recognized pregnancy and pregnancy outcome in Uganda. *AIDS* 2004; **18**(5): 799-804.
7. Tweya H, Feldacker C, Breeze E, et al. Incidence of pregnancy among women accessing antiretroviral therapy in urban Malawi: a retrospective cohort study. *AIDS and behavior* 2013; **17**(2): 471-8.
8. Newell ML, Coovadia H, Cortina-Borja M, et al. Mortality of infected and uninfected infants born to HIV-infected mothers in Africa: a pooled analysis. *Lancet (London, England)* 2004; **364**(9441): 1236-43.
9. Badri M, Lawn SD, Wood R. Short-term risk of AIDS or death in people infected with HIV-1 before antiretroviral therapy in South Africa: a longitudinal study. *Lancet (London, England)* 2006; **368**(9543): 1254-9.
10. Kenya National Bureau of S, Ministry of HK, National ACCK, Kenya Medical Research I, National Council for P, Development/Kenya. Kenya Demographic and Health Survey 2014. Rockville, MD, USA, 2015.
11. Garnett GP, Anderson RM. Factors controlling the spread of HIV in heterosexual communities in developing countries: patterns of mixing between different age and sexual activity classes. *Philosophical transactions of the Royal Society of London Series B, Biological sciences* 1993; **342**(1300): 137-59.
12. Ferry B, Caraël M, Buvé A, et al. Comparison of key parameters of sexual behaviour in four African urban populations with different levels of HIV infection. *AIDS* 2001; **15**: S41-S50.
13. Datta P, Embree JE, Kreiss JK, et al. Mother-to-child transmission of human immunodeficiency virus type 1: report from the Nairobi Study. *J Infect Dis* 1994; **170**(5): 1134-40.
14. Waruru A, Achia TNO, Muttai H, et al. Spatial-temporal trend for mother-to-child transmission of HIV up to infancy and during pre-Option B+ in western Kenya, 2007-13. *PeerJ* 2018; **6**: e4427.
15. Quinn TC, Wawer MJ, Sewankambo N, et al. Viral load and heterosexual transmission of human immunodeficiency virus type 1. Rakai Project Study Group. *The New England journal of medicine* 2000; **342**(13): 921-9.
16. Hubert JB, Burgard M, Dussaix E, et al. Natural history of serum HIV-1 RNA levels in 330 patients with a known date of infection. The SEROCO Study Group. *AIDS* 2000; **14**(2): 123-31.
17. Lingappa JR, Hughes JP, Wang RS, et al. Estimating the impact of plasma HIV-1 RNA reductions on heterosexual HIV-1 transmission risk. *PLoS One* 2010; **5**(9): e12598.
18. Hollingsworth TD, Anderson RM, Fraser C. HIV-1 transmission, by stage of infection. *The Journal of infectious diseases* 2008; **198**(5): 687-93.
19. Gray RH, Wawer MJ, Brookmeyer R, et al. Probability of HIV-1 transmission per coital act in monogamous, heterosexual, HIV-1-discordant couples in Rakai, Uganda. *Lancet (London, England)* 2001; **357**(9263): 1149-53.
20. Boily MC, Baggaley RF, Wang L, et al. Heterosexual risk of HIV-1 infection per sexual act: systematic review and meta-analysis of observational studies. *The Lancet Infectious diseases* 2009; **9**(2): 118-29.
21. Houlihan CF, Larke NL, Watson-Jones D, et al. Human papillomavirus infection and increased risk of HIV acquisition. A systematic review and meta-analysis. *AIDS (London, England)* 2012; **26**(17): 2211-22.
22. Looker KJ, Rönn MM, Brock PM, et al. Evidence of synergistic relationships between HIV and Human Papillomavirus (HPV): systematic reviews and meta-analyses of longitudinal studies of HPV acquisition and clearance by HIV status, and of HIV acquisition by HPV status. *J Int AIDS Soc* 2018; **21**(6): e25110-e.
23. Auvert B, Marais D, Lissouba P, Zarca K, Ramjee G, Williamson A-L. High-risk human papillomavirus is associated with HIV acquisition among South African female sex workers. *Infect Dis Obstet Gynecol* 2011; **2011**: 692012-.
24. Averbach SH, Gravitt PE, Nowak RG, et al. The association between cervical human papillomavirus infection and HIV acquisition among women in Zimbabwe. *AIDS* 2010; **24**(7): 1035-42.
25. Nowak RG, Gravitt PE, Morrison CS, et al. Increases in human papillomavirus detection during early HIV infection among women in Zimbabwe. *J Infect Dis* 2011; **203**(8): 1182-91.

26. Low AJ, Clayton T, Konate I, et al. Genital warts and infection with human immunodeficiency virus in high-risk women in Burkina Faso: a longitudinal study. *BMC Infect Dis* 2011; **11**: 20-.
27. Myer L, Denny L, Wright TC, Kuhn L. Prospective study of hormonal contraception and women's risk of HIV infection in South Africa. *Int J Epidemiol* 2007; **36**(1): 166-74.
28. Smith-McCune KK, Shiboski S, Chirenje MZ, et al. Type-specific cervico-vaginal human papillomavirus infection increases risk of HIV acquisition independent of other sexually transmitted infections. *PLoS One* 2010; **5**(4): e10094-e.
29. Gallagher KE, Baisley K, Grosskurth H, et al. The Association Between Cervical Human Papillomavirus Infection and Subsequent HIV Acquisition in Tanzanian and Ugandan Women: A Nested Case-Control Study. *J Infect Dis* 2016; **214**(1): 87-95.
30. Veldhuijzen NJ, Vyankandondera J, van de Wijgert JH. HIV acquisition is associated with prior high-risk human papillomavirus infection among high-risk women in Rwanda. *AIDS* 2010; **24**(14): 2289-92.
31. Wang C, Wright TC, Denny L, Kuhn L. Rapid rise in detection of human papillomavirus (HPV) infection soon after incident HIV infection among South African women. *J Infect Dis* 2011; **203**(4): 479-86.
32. Tanser F, Jones KG, Viljoen J, Imrie J, Grapsa E, Newell M-L. Human papillomavirus seropositivity and subsequent risk of HIV acquisition in rural South African women. *Sex Transm Dis* 2013; **40**(7): 601-6.
33. Liu G, Mugo NR, Brown ER, et al. Prevalent HPV infection increases the risk of HIV acquisition in African women: advancing the argument for HPV immunization. *AIDS* 2021.
34. Franco EL, Villa LL, Sobrinho JP, et al. Epidemiology of acquisition and clearance of cervical human papillomavirus infection in women from a high-risk area for cervical cancer. *J Infect Dis* 1999; **180**(5): 1415-23.
35. Lodi S, Phillips A, Touloumi G, et al. Time from human immunodeficiency virus seroconversion to reaching CD4+ cell count thresholds <200, <350, and <500 Cells/mm(3): assessment of need following changes in treatment guidelines. *Clinical infectious diseases : an official publication of the Infectious Diseases Society of America* 2011; **53**(8): 817-25.
36. Lyles RH, Munoz A, Yamashita TE, et al. Natural history of human immunodeficiency virus type 1 viremia after seroconversion and proximal to AIDS in a large cohort of homosexual men. Multicenter AIDS Cohort Study. *The Journal of infectious diseases* 2000; **181**(3): 872-80.
37. Pantazis N, Morrison C, Amornkul PN, et al. Differences in HIV natural history among African and non-African seroconverters in Europe and seroconverters in sub-Saharan Africa. *PLoS One* 2012; **7**(3): e32369.
38. Lewden C, Gabillard D, Minga A, et al. CD4-specific mortality rates among HIV-infected adults with high CD4 counts and no antiretroviral treatment in West Africa. *Journal of acquired immune deficiency syndromes (1999)* 2012; **59**(2): 213-9.
39. Maduna PH, Dolan M, Kondlo L, et al. Morbidity and mortality according to latest CD4+ cell count among HIV positive individuals in South Africa who enrolled in project Phidisa. *PLoS One* 2015; **10**(4): e0121843.
40. Adler WH, Baskar PV, Chrest FJ, Dorsey-Cooper B, Winchurch RA, Nagel JE. HIV infection and aging: mechanisms to explain the accelerated rate of progression in the older patient. *Mech Ageing Dev* 1997; **96**(1-3): 137-55.
41. Brinkhof MW, Boulle A, Weigel R, et al. Mortality of HIV-infected patients starting antiretroviral therapy in sub-Saharan Africa: comparison with HIV-unrelated mortality. *PLoS medicine* 2009; **6**(4): e1000066.
42. Cornell M, Johnson LF, Wood R, et al. Twelve-year mortality in adults initiating antiretroviral therapy in South Africa. *Journal of the International AIDS Society* 2017; **20**(1): 21902.
43. de Coninck Z, Hussain-Alkhateeb L, Bratt G, et al. Non-AIDS Mortality Is Higher Among Successfully Treated People Living with HIV Compared with Matched HIV-Negative Control Persons: A 15-Year Follow-Up Cohort Study in Sweden. *AIDS patient care and STDs* 2018; **32**(8): 297-305.

44. Bouvard V, Baan R, Straif K, et al. A review of human carcinogens--Part B: biological agents. *The Lancet Oncology* 2009; **10**(4): 321-2.
45. Burchell AN, Richardson H, Mahmud SM, et al. Modeling the sexual transmissibility of human papillomavirus infection using stochastic computer simulation and empirical data from a cohort study of young women in Montreal, Canada. *Am J Epidemiol* 2006; **163**(6): 534-43.
46. Denny L, Adewole I, Anorlu R, et al. Human papillomavirus prevalence and type distribution in invasive cervical cancer in sub-Saharan Africa. *International journal of cancer* 2014; **134**(6): 1389-98.
47. Dartell M, Rasch V, Kahesa C, et al. Human papillomavirus prevalence and type distribution in 3603 HIV-positive and HIV-negative women in the general population of Tanzania: the PROTECT study. *Sexually transmitted diseases* 2012; **39**(3): 201-8.
48. Beachler DC, Jenkins G, Safaeian M, Kreimer AR, Wentzensen N. Natural Acquired Immunity Against Subsequent Genital Human Papillomavirus Infection: A Systematic Review and Meta-analysis. *J Infect Dis* 2016; **213**(9): 1444-54.
49. Johnson HC, Elfstrom KM, Edmunds WJ. Inference of type-specific HPV transmissibility, progression and clearance rates: a mathematical modelling approach. *PLoS One* 2012; **7**(11): e49614.
50. Tan N, Sharma M, Winer R, Galloway D, Rees H, Barnabas RV. Model-estimated effectiveness of single dose 9-valent HPV vaccination for HIV-positive and HIV-negative females in South Africa. *Vaccine* 2018; **36**(32 Pt A): 4830-6.
51. Liu G, Sharma M, Tan N, Barnabas RV. HIV-positive women have higher risk of human papilloma virus infection, precancerous lesions, and cervical cancer. *AIDS (London, England)* 2018; **32**(6): 795-808.
52. Kelly H, Weiss HA, Benavente Y, de Sanjose S, Mayaud P. Association of antiretroviral therapy with high-risk human papillomavirus, cervical intraepithelial neoplasia, and invasive cervical cancer in women living with HIV: a systematic review and meta-analysis. *The lancet HIV* 2018; **5**(1): e45-e58.
53. Suehiro TT, Damke G, Damke E, et al. Cervical and oral human papillomavirus infection in women living with human immunodeficiency virus (HIV) and matched HIV-negative controls in Brazil. *Infectious agents and cancer* 2020; **15**: 31.
54. Rohner E, Bütikofer L, Schmidlin K, et al. Cervical cancer risk in women living with HIV across four continents: A multicohort study. *Int J Cancer* 2020; **146**(3): 601-9.
55. Rohner E, Sengayi M, Goeieman B, et al. Cervical cancer risk and impact of Pap-based screening in HIV-positive women on antiretroviral therapy in Johannesburg, South Africa. *International journal of cancer* 2017; **141**(3): 488-96.
56. Sankaranarayanan R, Swaminathan R, Brenner H, et al. Cancer survival in Africa, Asia, and Central America: a population-based study. *Lancet Oncol* 2010; **11**(2): 165-73.
57. Dryden-Peterson S, Bvochora-Nsingo M, Suneja G, et al. HIV Infection and Survival Among Women With Cervical Cancer. *Journal of clinical oncology : official journal of the American Society of Clinical Oncology* 2016; **34**(31): 3749-57.
58. Liu G, Sharma M, Tan N, Barnabas RV. HIV-positive women have higher risk of human papilloma virus infection, precancerous lesions, and cervical cancer. *AIDS* 2018; **32**(6): 795-808.
59. Campos NG, Burger EA, Sy S, et al. An updated natural history model of cervical cancer: derivation of model parameters. *Am J Epidemiol* 2014; **180**(5): 545-55.
60. Rosillon D, Baril L, Del Rosario-Raymundo MR, et al. Risk of newly detected infections and cervical abnormalities in adult women seropositive or seronegative for naturally acquired HPV-16/18 antibodies. *Cancer Med* 2019; **8**(10): 4938-53.
61. van Schalkwyk C, Moodley J, Welte A, Johnson LF. Modelling the impact of prevention strategies on cervical cancer incidence in South Africa. *Int J Cancer* 2021; **149**(8): 1564-75.
62. Myers ER, McCrory DC, Nanda K, Bastian L, Matchar DB. Mathematical model for the natural history of human papillomavirus infection and cervical carcinogenesis. *Am J Epidemiol* 2000; **151**(12): 1158-71.

63. Campos NG, Lince-Deroche N, Chibwesha CJ, et al. Cost-Effectiveness of Cervical Cancer Screening in Women Living With HIV in South Africa: A Mathematical Modeling Study. *J Acquir Immune Defic Syndr* 2018; **79**(2): 195-205.
64. Lilian RR, Rees K, Mabitsi M, McIntyre JA, Struthers HE, Peters RPH. Baseline CD4 and mortality trends in the South African human immunodeficiency virus programme: Analysis of routine data. *South Afr J HIV Med* 2019; **20**(1): 963.
65. Rodger AJ, Cambiano V, Bruun T, et al. Sexual Activity Without Condoms and Risk of HIV Transmission in Serodifferent Couples When the HIV-Positive Partner Is Using Suppressive Antiretroviral Therapy. *Jama* 2016; **316**(2): 171-81.
66. Eisinger RW, Dieffenbach CW, Fauci AS. HIV Viral Load and Transmissibility of HIV Infection: Undetectable Equals Untransmittable. *Jama* 2019; **321**(5): 451-2.
67. Kenya Ministry of Health National AIDS & STI Control Program. The Guidelines on Use of Antiretroviral Drugs for Treating and Preventing HIV Infection in Kenya. Nairobi: Kenya Ministry of Health 2018.
68. Kenya Ministry of Health. Kenya AIDS Response Progress Report 2016.
69. Kenya Ministry of Health National AIDS & STI Control Program. Kenya AIDS Response Progress Report, Progress Towards Zero. Nairobi, Kenya: Kenya Ministry of Health, 2014.
70. Kenya Ministry of Health National AIDS & STI Control Program. Kenya Population-based HIV Impact Assessment (KENPHIA) 2018. Nairobi, Kenya: National AIDS & STI Control Program, 2020.
71. Karcher H, Omondi A, Odera J, Kunz A, Harms G. Risk factors for treatment denial and loss to follow-up in an antiretroviral treatment cohort in Kenya. *Tropical medicine & international health : TM & IH* 2007; **12**(5): 687-94.
72. Zachariah R, Van Engelgem I, Massaquoi M, et al. Payment for antiretroviral drugs is associated with a higher rate of patients lost to follow-up than those offered free-of-charge therapy in Nairobi, Kenya. *Transactions of the Royal Society of Tropical Medicine and Hygiene* 2008; **102**(3): 288-93.
73. Kenya Ministry of Health National AIDS & STI Control Program. AIDS Response Progress Report Nairobi, Kenya: Kenya Ministry of Health 2016.
74. Waithaka M, Bessinger R. Sexual Behavior and Condom Use in the Context of HIV Prevention in Kenya: Population Services International, 2001.
75. Cherutich P, Brentlinger P, Nduati R, Kiarie JN, Farquhar C. Condom use among sexually active Kenyan female adolescents at risk for HIV-1 infection. *AIDS and behavior* 2008; **12**(6): 923-9.
76. Weller S, Davis K. Condom effectiveness in reducing heterosexual HIV transmission. *Cochrane Database Syst Rev* 2002; (1): CD003255.
77. Manhart LE, Koutsky LA. Do condoms prevent genital HPV infection, external genital warts, or cervical neoplasia? A meta-analysis. *Sex Transm Dis* 2002; **29**(11): 725-35.
78. Ho GY, Studentsov YY, Bierman R, Burk RD. Natural history of human papillomavirus type 16 virus-like particle antibodies in young women. *Cancer epidemiology, biomarkers & prevention : a publication of the American Association for Cancer Research, cosponsored by the American Society of Preventive Oncology* 2004; **13**(1): 110-6.
79. Moscicki AB, Hills N, Shiboski S, et al. Risks for incident human papillomavirus infection and low-grade squamous intraepithelial lesion development in young females. *JAMA* 2001; **285**(23): 2995-3002.
80. Sanchez-Aleman MA, Uribe-Salas FJ, Lazcano-Ponce EC, Conde-Glez CJ. Human papillomavirus incidence and risk factors among Mexican female college students. *Sex Transm Dis* 2011; **38**(4): 275-8.
81. Siegfried N, Muller M, Deeks JJ, Volmink J. Male circumcision for prevention of heterosexual acquisition of HIV in men. *Cochrane Database of Systematic Reviews* 2009; (2).
82. Albero G, Castellsagué X, Lin H-Y, et al. Male circumcision and the incidence and clearance of genital human papillomavirus (HPV) infection in men: the HPV Infection in men (HIM) cohort study. *BMC Infect Dis* 2014; **14**: 75-.

83. Albero G, Villa LL, Lazcano-Ponce E, et al. Male circumcision and prevalence of genital human papillomavirus infection in men: a multinational study. *BMC Infect Dis* 2013; **13**: 18-.
84. Vanbuskirk K, Winer RL, Hughes JP, et al. Circumcision and acquisition of human papillomavirus infection in young men. *Sex Transm Dis* 2011; **38**(11): 1074-81.
85. Tobian AAR, Kong X, Gravitt PE, et al. Male circumcision and anatomic sites of penile high-risk human papillomavirus in Rakai, Uganda. *Int J Cancer* 2011; **129**(12): 2970-5.
86. Tobian AA, Kong X, Wawer MJ, et al. Circumcision of HIV-infected men and transmission of human papillomavirus to female partners: analyses of data from a randomised trial in Rakai, Uganda. *The Lancet Infectious diseases* 2011; **11**(8): 604-12.
87. Weiss HA, Hankins CA, Dickson K. Male circumcision and risk of HIV infection in women: a systematic review and meta-analysis. *The Lancet Infectious diseases* 2009; **9**(11): 669-77.
88. Lei JH, Liu LR, Wei Q, et al. Circumcision Status and Risk of HIV Acquisition during Heterosexual Intercourse for Both Males and Females: A Meta-Analysis. *PLoS One* 2015; **10**(5): e0125436.
89. Wawer MJ, Tobian AAR, Kigozi G, et al. Effect of circumcision of HIV-negative men on transmission of human papillomavirus to HIV-negative women: a randomised trial in Rakai, Uganda. *Lancet (London, England)* 2011; **377**(9761): 209-18.
90. Shaffer DN, Bautista CT, Sateren WB, et al. The protective effect of circumcision on HIV incidence in rural low-risk men circumcised predominantly by traditional circumcisers in Kenya: two-year follow-up of the Kericho HIV Cohort Study. *J Acquir Immune Defic Syndr* 2007; **45**(4): 371-9.
91. Central Bureau of Statistics CBSK, Ministry of Health MOHK, Macro ORC. Kenya Demographic and Health Survey 2003. Calverton, Maryland, USA: CBS, MOH, and ORC Macro, 2004.
92. Fleming PJ, Doshi M, Harper GW, Otieno F, Bailey RC. Integration of voluntary male medical circumcision for HIV prevention into norms of masculinity: findings from Kisumu, Kenya. *Culture, health & sexuality* 2020: 1-13.
93. Kenya National Bureau of Statistics - KNBS, National AIDS Control Council/Kenya, National AIDS/STD Control Programme/Kenya, Health MoP, Sanitation/Kenya, Kenya Medical Research Institute. Kenya Demographic and Health Survey 2008-09. Calverton, Maryland, USA: KNBS and ICF Macro, 2010.
94. de Sanjose S, Quint WG, Alemany L, et al. Human papillomavirus genotype attribution in invasive cervical cancer: a retrospective cross-sectional worldwide study. *Lancet Oncol* 2010; **11**(11): 1048-56.
95. Daniels V, Saxena K, Patterson-Lomba O, et al. Modeling the health and economic implications of adopting a 1-dose 9-valent human papillomavirus vaccination regimen in a high-income country setting: An analysis in the United Kingdom. *Vaccine* 2022; **40**(14): 2173-83.
96. Kjaer SK, Sigurdsson K, Iversen OE, et al. A pooled analysis of continued prophylactic efficacy of quadrivalent human papillomavirus (Types 6/11/16/18) vaccine against high-grade cervical and external genital lesions. *Cancer Prev Res (Phila)* 2009; **2**(10): 868-78.
97. Barnabas RV, Brown ER, Onono MA, et al. Durability of single-dose HPV vaccination in young Kenyan women: randomized controlled trial 3-year results. *Nature Medicine* 2023.
98. Karanja-Chege CM. HPV Vaccination in Kenya: The Challenges Faced and Strategies to Increase Uptake. *Front Public Health* 2022; **10**: 802947.
99. Mwenda V. Country-Specific Example: Kenya's Efforts to Implement Cervical Screening and Treatment Following the WHO Guidelines. The 35th International Papillomavirus Conference. Washington D.C., United States; 2023.
100. Kenya Ministry of Health and Sanitation. National Guidelines for Prevention and Management of Cervical, Breast and Prostate Cancers. Nairobi, Kenya: Kenya Ministry of Health and Sanitation, 2012.
101. Gakidou E, Nordhagen S, Obermeyer Z. Coverage of cervical cancer screening in 57 countries: low average levels and large inequalities. *PLoS Med* 2008; **5**(6): e132-e.
102. Kenya, Human Papillomavirus and Related Cancers, Fact Sheet 2023. 2023-03-10 2023 (accessed October 4 2023).

103. Kemper KE, McGrath CJ, Eckert LO, et al. Correlates of cervical cancer screening among women living with HIV in Kenya: A cross-sectional study. *International Journal of Gynecology & Obstetrics* 2022; **156**(1): 151-8.
104. Chung MH, McKenzie KP, De Vuyst H, et al. Comparing Papanicolau smear, visual inspection with acetic acid and human papillomavirus cervical cancer screening methods among HIV-positive women by immune status and antiretroviral therapy. *AIDS* 2013; **27**(18): 2909-19.
105. Khozaim K, Orang'o E, Christoffersen-Deb A, et al. Successes and challenges of establishing a cervical cancer screening and treatment program in western Kenya. *Int J Gynaecol Obstet* 2014; **124**(1): 12-8.
106. Greene SA, De Vuyst H, John-Stewart GC, et al. Effect of Cryotherapy vs Loop Electrosurgical Excision Procedure on Cervical Disease Recurrence Among Women With HIV and High-Grade Cervical Lesions in Kenya: A Randomized Clinical Trial. *JAMA* 2019; **322**(16): 1570-9.
107. Kuhn L, Wang C, Tsai WY, Wright TC, Denny L. Efficacy of human papillomavirus-based screen-and-treat for cervical cancer prevention among HIV-infected women. *AIDS* 2010; **24**(16): 2553-61.
108. Msyamboza KP, Phiri T, Sichali W, Kwenda W, Kachale F. Cervical cancer screening uptake and challenges in Malawi from 2011 to 2015: retrospective cohort study. *BMC Public Health* 2016; **16**(1): 806.
109. Ouedraogo Y, Furlane G, Fruhauf T, et al. Expanding the Single-Visit Approach for Cervical Cancer Prevention: Successes and Lessons From Burkina Faso. *Glob Health Sci Pract* 2018; **6**(2): 288-98.
110. Catarino R, Petignat P, Dongui G, Vassilakos P. Cervical cancer screening in developing countries at a crossroad: Emerging technologies and policy choices. *World J Clin Oncol* 2015; **6**(6): 281-90.
111. Sherris J, Wittet S, Kleine A, et al. Evidence-based, alternative cervical cancer screening approaches in low-resource settings. *Int Perspect Sex Reprod Health* 2009; **35**(3): 147-54.
112. Hoffman SR, Le T, Lockhart A, et al. Patterns of persistent HPV infection after treatment for cervical intraepithelial neoplasia (CIN): A systematic review. *Int J Cancer* 2017; **141**(1): 8-23.
113. Mungo C, Randa M, Shauri A, et al. Characteristics, stage at presentation, and status of women with cervical cancer at a major referral center in western Kenya. *International Journal of Gynecology & Obstetrics* 2022; **156**(1): 173-4.
114. Ferlay J, Soerjomataram I, Dikshit R, et al. Cancer incidence and mortality worldwide: sources, methods and major patterns in GLOBOCAN 2012. *Int J Cancer* 2015; **136**(5): E359-86.
115. Jedy-Agba E, Joko WY, Liu B, et al. Trends in cervical cancer incidence in sub-Saharan Africa. *British Journal of Cancer* 2020; **123**(1): 148-54.
116. Kenya Ministry of Health and Sanitation. 2007 Kenya AIDS Indicator Survey (KAIS). Nairobi, Kenya: Kenya Ministry of Health and Sanitation,, 2009.
117. Kenya Ministry of Health. 2012 Kenya AIDS Indicator Survey (KAIS). Nairobi, Kenya: Ministry of Health 2014.
118. Luchters SMF, Broeck DV, Chersich MF, et al. Association of HIV infection with distribution and viral load of HPV types in Kenya: a survey with 820 female sex workers. *BMC Infect Dis* 2010; **10**(1): 18.
119. Yamada R, Sasagawa T, Kirumbi LW, et al. Human papillomavirus infection and cervical abnormalities in Nairobi, Kenya, an area with a high prevalence of human immunodeficiency virus infection. *J Med Virol* 2008; **80**(5): 847-55.
120. Bruni L AG, Serrano B, Mena M, Gómez D, Muñoz J, Bosch FX, de Sanjosé S. Human Papillomavirus and Related Diseases in South Africa: Summary Report 17 June 2019.
121. Van Aardt MC, Dreyer G, Richter KL, Becker P. Human papillomavirus-type distribution in South African women without cytological abnormalities: a peri-urban study. *Southern African Journal of Gynaecological Oncology* 2013; **5**(sup1): S21-S7.
122. Clifford GM, Rana RK, Franceschi S, Smith JS, Gough G, Pimenta JM. Human papillomavirus genotype distribution in low-grade cervical lesions: comparison by geographic region and with cervical cancer. *Cancer epidemiology, biomarkers & prevention : a publication of the American Association for Cancer Research, cosponsored by the American Society of Preventive Oncology* 2005; **14**(5): 1157-64.

123. Van Aardt MC, Dreyer G, Snyman LC, Richter KL, Becker P, Mojaki SM. Oncogenic and incidental HPV types associated with histologically confirmed cervical intraepithelial neoplasia in HIV-positive and HIV-negative South African women. *S Afr Med J* 2016; **106**(6).
124. van Aardt MC, Dreyer G, Pienaar HF, et al. Unique human papillomavirus-type distribution in South African women with invasive cervical cancer and the effect of human immunodeficiency virus infection. *International journal of gynecological cancer : official journal of the International Gynecological Cancer Society* 2015; **25**(5): 919-25.
125. Sung H, Ferlay J, Siegel RL, et al. Global cancer statistics 2020: GLOBOCAN estimates of incidence and mortality worldwide for 36 cancers in 185 countries. *CA: a cancer journal for clinicians* 2021.
126. Rao DW, Bayer CJ, Liu G, et al. Modelling cervical cancer elimination using single-visit screening and treatment strategies in the context of high HIV prevalence: estimates for KwaZulu-Natal, South Africa. *J Int AIDS Soc* 2022; **25**(10): e26021.
127. Kelly H, Mayaud P, Segondy M, Pant Pai N, Peeling RW. A systematic review and meta-analysis of studies evaluating the performance of point-of-care tests for human papillomavirus screening. *Sex Transm Infect* 2017; **93**(S4): S36-s45.
128. Canfell K, Kim JJ, Kulasingam S, et al. HPV-FRAME: A consensus statement and quality framework for modelled evaluations of HPV-related cancer control. *Papillomavirus Res* 2019; **8**: 100184.
129. Husereau D, Drummond M, Augustovski F, et al. Consolidated Health Economic Evaluation Reporting Standards 2022 (CHEERS 2022) Statement: Updated Reporting Guidance for Health Economic Evaluations. *Value in Health* 2022; **25**(1): 3-9.
